# Aminopyrimidine–galactose hybrids are highly selective galectin-3 inhibitors†
†Electronic supplementary information (ESI) available: ^1^H NMR and ^13^C NMR spectra for all new compounds. See DOI: 10.1039/c9md00183b


**DOI:** 10.1039/c9md00183b

**Published:** 2019-05-13

**Authors:** Alexander Dahlqvist, Fredrik R. Zetterberg, Hakon Leffler, Ulf J. Nilsson

**Affiliations:** a Centre for Analysis and Synthesis , Department of Chemistry , Lund University , Box 124 , SE-221 00 Lund , Sweden . Email: ulf.nilsson@chem.lu.se; b Galecto Biotech AB , Sahlgrenska Science Park, Medicinaregatan 8A , SE-413 46 Gothenburg , Sweden; c Department of Laboratory Medicine , Section MIG , Lund University BMC-C1228b , Klinikgatan 28 , SE-221 84 Lund , Sweden

## Abstract

Galectins are a family of carbohydrate recognition proteins involved in, among other things, modulating cell signalling and cell–environment interactions, giving them roles in several pathologies like cancer and idiopathic lung fibrosis. Hence, developing new galectin inhibitors with high affinity and high selectivity is important to be able to target such diseases. Most existing galectin inhibitors have a disaccharide scaffold, but there has been success as of late in developing monogalactoside inhibitors such as α-arylthioglycosides. Here, we report aminopyrimidine-derivatised galactosides as good galectin-3 inhibitors with affinities down to 1.7 μM and a more than 300-fold selectivity over galectin-1. Mutant studies replacing Arg144 in galectin-3 with lysine and serine support the hypothesis that the binding of the derivatives involves interactions with Arg144. Molecular dynamics simulations converged to stable poses of the inhibitor aminopyrimidine moiety with polar interactions with Asp148 and Ser237, while the aryl-aminopyrimidine ring stacked onto the side chain of Arg144. Hence, combining an aminopyrimidine motif with a phenyl α-thiogalactoside motif offers an attractive route towards highly selective galectin-3 inhibitors.

## Abstract 2

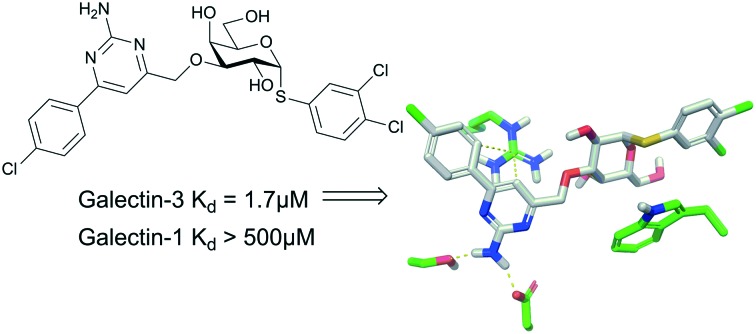
Galactopyranosides with aryl-aminopyrimidine moieties at O3 inhibit the tumor and immunity-related galectin-3 with high selectivity over other galectins.

## Introduction

**Fig. 1:**
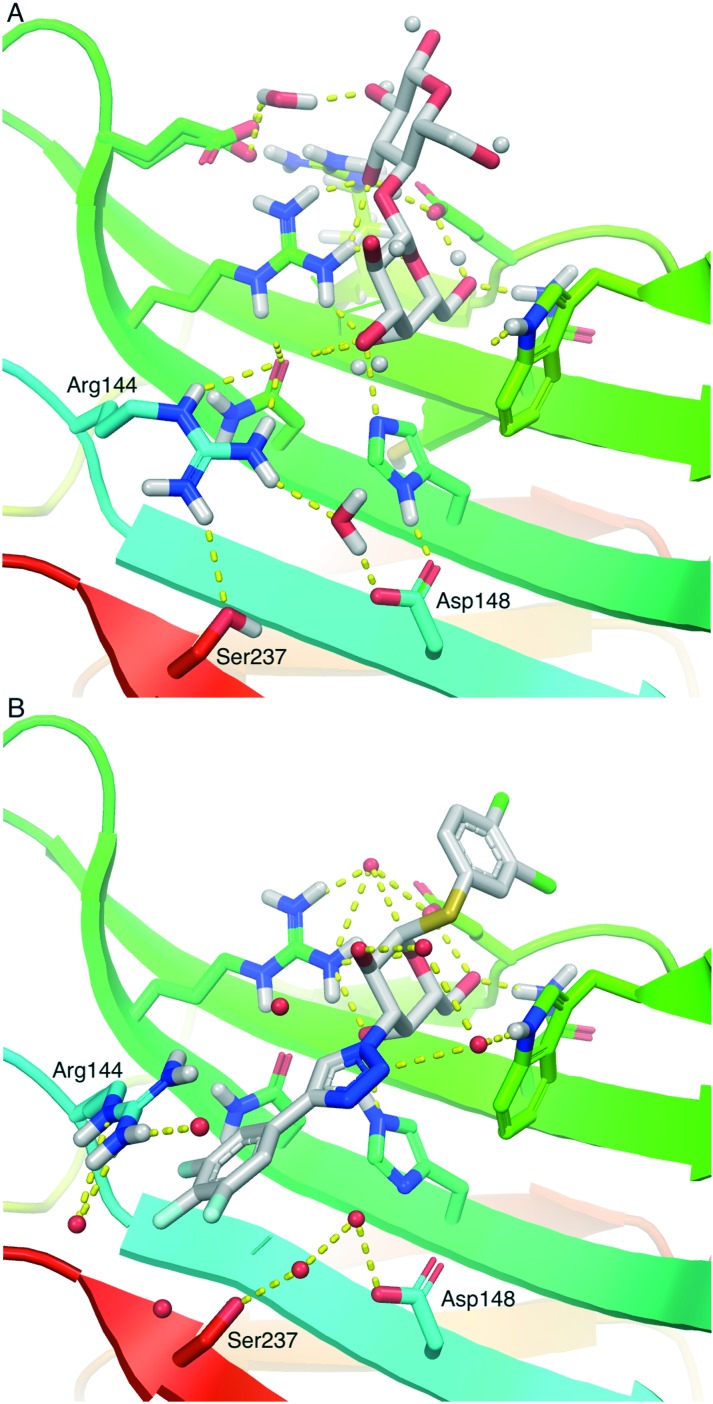
(A) Neutron structure (6EYM) of lactose in complex with galectin-3C in which the water and its directionality in bridging Asp148 and Arg144 are depicted. Lactose hydroxyl hydrogens are shown as white spheres.^14^ (B) X-ray structure (; 6EOL) of a 3C-triazolyl α-thiogalactoside in complex with galectin-3 in which a trifluorophenyltriazole inserts between Asp148 and Arg144.^17^ Images were generated using PyMOL v1.7 (Schrodinger LLC).

Galectins are a family of galactoside-binding proteins that are involved in a variety of molecular processes, such as binding cell surface glycoproteins to form lattices. This influences, among other things, membrane residence time and trafficking of glycoproteins, which can have a marked effect on glycoprotein cellular function.^1^,^2^ Glycoproteins that are ligands to galectins include vascular endothelial growth factor receptor,^3^,^4^ epidermal growth factor receptor, and transforming growth factor beta receptor.^5^ Interaction with glycoproteins can give galectins roles in regulating cell signalling and cell adhesion, which in turn is reflected in their role in, for example, angiogenesis,^6^ pathological lymphangiogenesis,^4^ idiopathic lung fibrosis,^7^ and a variety of cancers.^8^ Galectin-3 inhibition is currently being evaluated as a treatment for idiopathic lung fibrosis.^9^ The galectins feature a conserved carbohydrate binding domain that is a shallow groove on top of two curved beta sheets large enough to accommodate approximately a tetrasaccharide and display a few differences between the different galectins. The galectins come in three major types: prototype galectins, which include galectin-1 and -7, feature a single carbohydrate recognition domain (CRD) with the ability to form homodimers. Tandem repeat galectins have two different CRDs bound by a linker and include galectin-4, -8 and -9. Galectin-3 is the sole member of the chimera galectins, a single CRD with a collagen-like tail and the ability to oligomerize. Galectin inhibitors have evolved from the natural binding motif lactose to synthetic derivatives, such as thiodigalactosides decorated with different non-carbohydrate structural elements.^10^–^13^ In complexes of galectin-3 with natural ligand fragments, such as lactose,^14^ the side chain of Arg144 forms a water-mediated interaction with Asp148 (Fig. 1A), while synthetic high-affinity inhibitors insert a benzamido or phenyltriazole aromatic ring between the Arg144 side chain and the water molecule (Fig. 1B).^13^,^15^,^16^ Hence, the galectin-3 Arg144–Asp148 water-mediated interaction is adaptable to accommodate different inhibitor structures and is thus an interesting target for novel affinity- and selectivity-enhancing structural elements. In this context, we hypothesized that aryl-aminopyrimidylmethyl substituents at galactose O3, synthesized from 3-*O*-propargyl galactoside derivatives, may be capable of engaging in polar interactions with Asp148 and Ser237, which in turn may lead to highly selective compounds towards galectin-3. Furthermore, high-affinity inhibitors based on α-arylthiogalactosides were recently reported,^17^ and based on this a viable hypothesis was that combining α-aryl aglycons with the above proposed aminopyrimidine structures at galactose O3 could lead to novel high-affinity galectin-3 inhibitors with improved selectivity.

## Results and discussion

### Chemistry

**Scheme 1:**
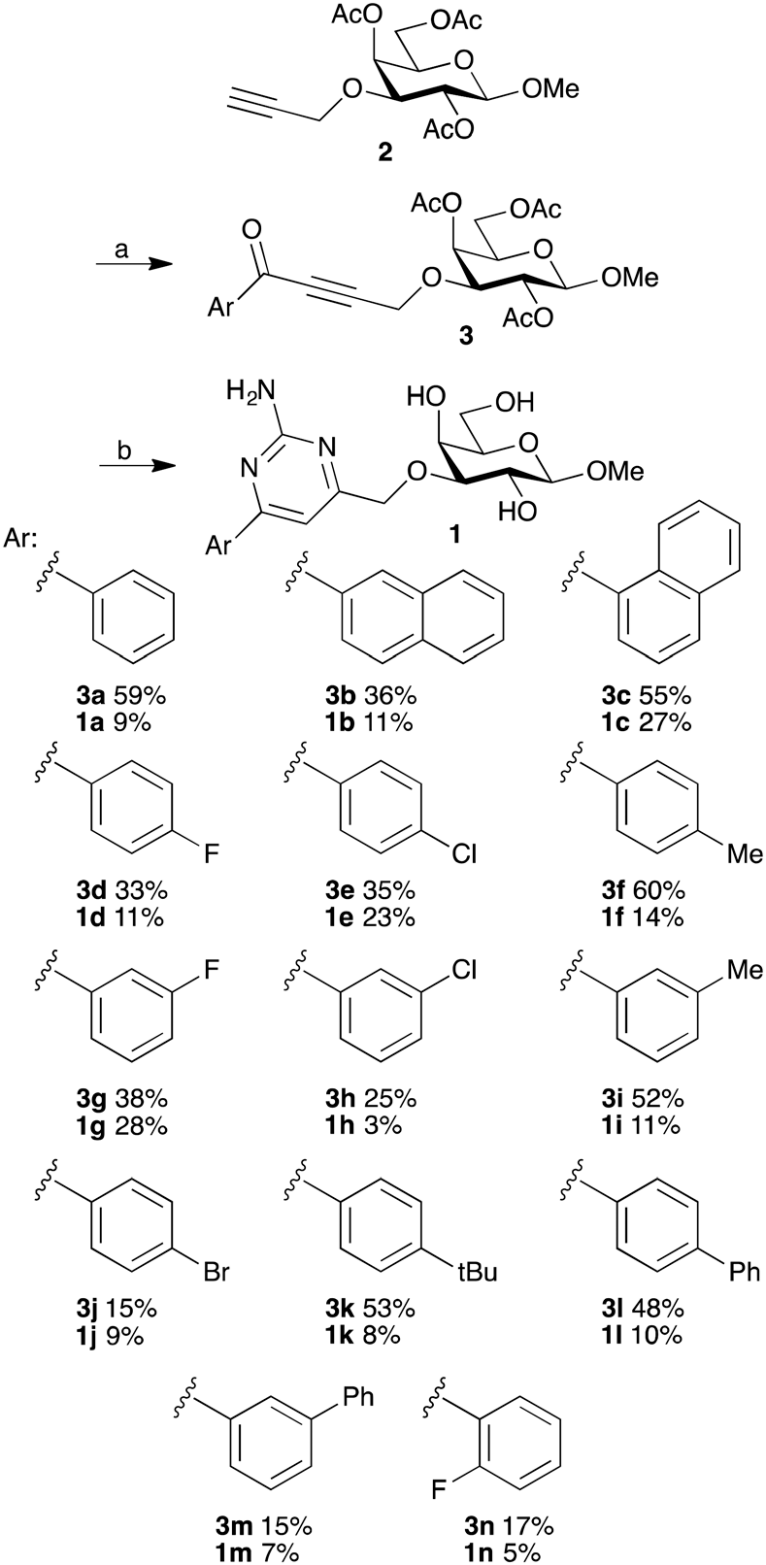
Synthesis of 3-*O*-(2-amino-4-aryl-pyrimidin-6-ylmethyl)-β-d-galactopyranosides **1a–n**. (a) Aroyl chloride, bis(triphenylphosphine)-palladium(ii) dichloride, copper(i) iodide, triethylamine, THF, rt, 18 h, 15–60%. (b) Guanidine hydrochloride, potassium carbonate, THF, reflux, 18 h, then sodium methoxide, methanol, 2 h, 5–28%.

The synthesis of methyl 3-(aryl)aminopyrimidinemethylene-β-d-galactopyranosides **1a–n** started with Sonogashira couplings of the 3-*O*-propargyl-galactoside **2** (ref. 18) to give the alkynones **3a–n** (Scheme 1). One-pot cyclizations of **3a–n** with guanidine hydrochloride and de-*O*-acetylation gave the aryl-aminopyrimidines **1a–n**.

### Galectin binding

The binding of **1a–n** and the reference ligand methyl β-d-galactopyranoside^19^ towards galectin-1, -3, -4N, -4C, -7, -8N, -8C, -9N, and -9C was measured using a competitive fluorescence polarization assay (Table 1).^20^ Affinities towards galectin-1 and galectin-3 are reported, while affinities towards other galectins were all in the millimolar range and are not shown. The aminopyrimidines **1a–n** generally display good galectin-3 affinities and good to excellent selectivities. The *para*-halogen series fluoro–chloro–bromo (**1d**, **1e** and **1j**) all have similar galectin-1 affinities close to 300 μM, while the selectivities differ. The 4-fluoro and 4-chloro **1d–e** have good selectivity for galectin-3 over galectin-1, while 4-bromo **1j** has only a slightly higher than twofold selectivity for galectin-3.

**Table 1:** Affinities (*K*_d_ in μM) of aminopyrimidines **1a–n** and the reference methyl β-d-galactopyranoside for galectin-1 and galectin-3

	Galectin-1	Galectin-3
**1a**	1400 ± 62^*a*^	260 ± 5
**1b**	>900^*b*^	83 ± 11
**1c**	1300 ± 160	340 ± 52
**1d**	1100 ± 230	320 ± 39
**1e**	>900	260 ± 15
**1f**	>900	270 ± 27
**1g**	1800 ± 330	230 ± 10
**1h**	1900 ± 130	190 ± 23
**1i**	200 ± 33	200 ± 19
**1j**	770 ± 28	310 ± 10
**1k**	1400 ± 360	300 ± 37
**1l**	>900	191 ± 25
**1m**	1000 ± 440	280 ± 41
**1n**	430 ± 45	260 ± 25
Me β-gal	>10 000	4400

^*a*^Compound solubilities allowed evaluation up to a concentration of 900 μM of **1a–1n**. *K*_d_ ± SEM values were calculated from 4 to 6 single point measurements at different concentrations for compounds that showed at least 20% inhibition at 900 μM inhibitor concentration.

^*b*^Inhibitors showing less than 20% inhibition at 900 μM concentration are given *K*_d_ values of >900 μM.

The 3-chloro and 3-fluoro derivatives **1g–h** have slightly higher affinities for galectin-3, but poorer selectivities over galectin-1 than the 4-substituted halides. The 4-methyl **1f** has the same galectin-3 affinity and selectivity as that of 4-chloro **1e**, while the 3-methyl **1i** has the same galectin-3 affinity as that of 3-chloro **1h** but no selectivity over galectin-1. The 2-fluoro **1n** displays poor selectivity, while having the same 200–300 μM affinity towards galectin-3 as most of the benzoylaminopyrimidines **1** have. The extended aryl system in 1-naphthyl **1c** has lower galectin-3 affinity and has poorer selectivity than most of the corresponding phenyl derivatives, while 2-naphthyl **1b** has the highest affinity of all the inhibitors, with a *K*_d_ of 83 μM and no measurable galectin-1 affinity. The slightly larger biphenyls **1l–m** have good galectin-3 affinities and higher than that of the 1-naphthyl **1c**, but lower than that of the 2-naphthyl **1b**. The 4-biphenyl **1l** has the second highest affinity of all the aminopyrimidines and an unmeasurably low galectin-1 affinity, while the 3-biphenyl **1m** is considerably less selective. Overall, it is possible to group the inhibitors into three groups: those with no selectivity (**1i**, **1j** and **1n**), those with a three- to fivefold selectivity (**1a**, **1c–d**, **1k–m**) and those with higher selectivities (**1b**, **1e–g** and **1h**). However, no clear preference for a position or type of substituent within any of the groups can be seen. In order to investigate if an interaction with the conformationally flexible galectin-3 Arg144 (ref. 15) near bound galactose O3 is important for the binding, the affinities of aminopyrimidines **1a–b** and **1e** towards two different galectin-3 mutants, R144K and R144S, were determined (Table 2). In R144K the Arg144 is replaced with a still positively charged lysine, and in R144S the Arg144 is replaced with a smaller, yet polar, side chain.

**Table 2:** Affinities (*K*_d_ in μM) of galectin-3 mutants for aminopyrimidines **1a–b** and **1e**

	**1a**	**1b**	**1e**
wt	260 ± 5^*a*^	83 ± 11	260 ± 15
R144K	840 ± 230	280 ± 20	1400 ± 90
R144S	1200 ± 580	1000 ± 44	1200 ± 450

^*a*^ *K*_d_ ± SEM values were calculated from 4 to 6 single point measurements at different concentrations.

**Scheme 2:**
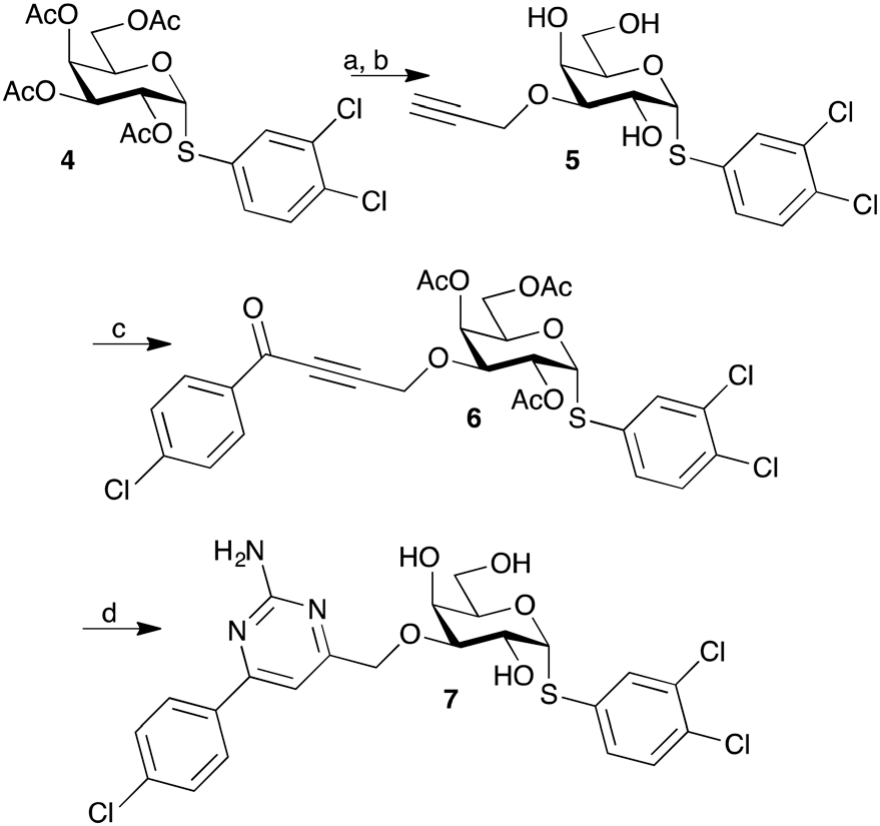
Synthesis of 3,4-dichlorophenyl 3-*O*-[(2-amino-4-(4-chlorophenyl)-pyrimidin-6-yl)methyl]-1-thio-α-d-galactopyranoside **7**. (a) NaOMe, MeOH; (b) dibutyltin oxide, dry methanol, reflux, 2 h, then propargyl bromide, tetrabutylammonium bromide, 1,4-dioxane, 80 °C, 52% from **4**; (c) 4-chlorobenzoyl chloride, bis(triphenylphosphine)palladium(ii) dichloride, copper(i) iodide, triethylamine, THF, 10 °C, 20 h, 63%; (d) guanidine hydrochloride, potassium carbonate, THF, reflux, 18 h, 18%.

The affinity of all of the inhibitors **1a–b** and **1e** towards the serine mutant R144S are in the millimolar range, which is to be expected if interactions with the Arg144 side chain are important. The lysine mutant R144K shows lower affinities towards the phenyl aminopyrimidines **1a** and **1e** than to the wild type and practically as low as that of the serine mutant, while the 2-naphthyl **1b** with its extended aromatic system has an affinity that is lower than that for the wild type, but still almost four times as good as the R144S affinity. The recovery of affinity by **1b** may be due to the 2-naphthyl **1b** having the ability to interact with the lysine side chain and possibly form a cation–π interaction, albeit less effectively than the interaction with the wild-type Arg144 side chain. Taken together, the mutant data suggest that Arg144–π interactions between galectin-3 and aminopyrimidine inhibitors **1a–b** and **1e** are important. Based on the high affinity, high selectivity, and comparatively good synthesis yields, the 4-chlorophenyl aminopyrimidine substituent motif of **1e** was selected for combination with a 3,4-dichlorophenyl α-thioglycosidic aglycon in the anomeric position known to increase affinity towards galectin-3.^17^ De-*O*-acetylation followed by regioselective 3-*O*-propargylation of the α-thiogalactoside **4** (ref. 17) gave **5**, which was subsequently benzoylated to give the alkynone **6**. One-pot cyclization and de-*O*-acetylation of **6** with guanidine hydrochloride gave the α-thio derivative **7** (Scheme 2).

Affinities of the aminopyrimidinyl α-thiogalactoside **7** towards galectin-1, -3, -4N, -4C, -7, -8N, -8C, -9N, and -9C (Table 3) shows that the affinity towards galectin-3 is greatly enhanced by the thio-linked aglycon, reaching a *K*_d_ of 1.7 μM. The best affinity towards any other galectin is 58 μM for galectin-7, which translates to a thirty-four-fold selectivity. Importantly, the selectivity between galectin-3 and galectin-1 is about 300-fold, which is a significant improvement over the reported 100-fold selectivity of the 3C-triazolyl-galactosides.^17^ Hence, although the affinity of **7** for galectin-3 is not as high as that of the corresponding 3C-triazole derivatives,^17^ the much higher selectivity of **7** is a significant advantage in experiments aiming at elucidating relative roles of galectin-3 *versus* other galectins, most notably galectin-1, and may be an advantage en route towards the development of more selective galectin-3 inhibitors.

**Table 3:** Galectin affinities (*K*_d_ in μM) of **7** towards galectin-1, -3, -4N, -4C, 7, 8N, 8C, 9N, and 9C

Galectin	*K*_d_
1	>500
3	1.7 ± 0.21^*a*^
4N	>500
4C	380 ± 90
7	58 ± 32
8N	170 ± 25
8C	>500
9N	73 ± 11
9C	>100

^*a*^ *K*_d_ ± SEM values were calculated from 4 to 6 single point measurements at different concentrations.

### Molecular modelling

**Fig. 2:**
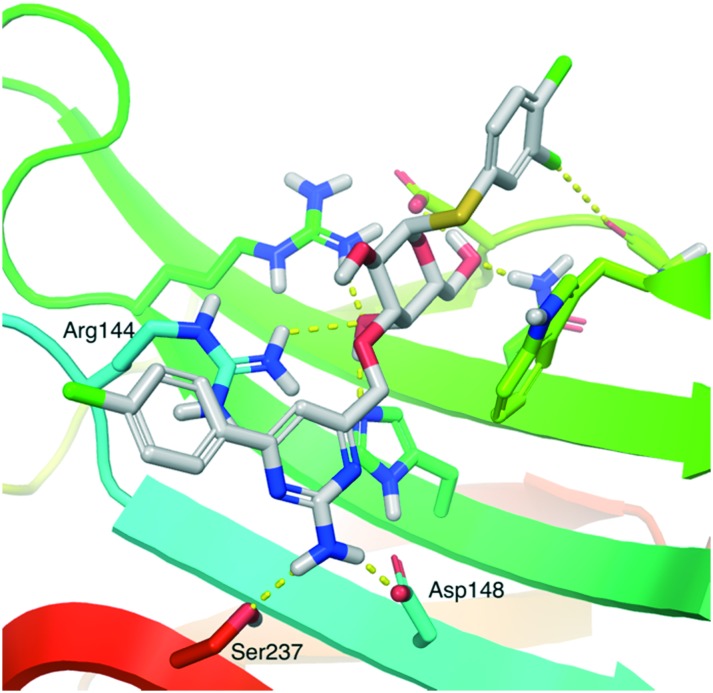
A representative MD snapshot of **7** in complex with galectin-3 where the amino group engages in direct hydrogen bonds to Asp148 and Ser237. The image was generated using PyMOL v1.7 (Schrodinger LLC).

In order to reach a plausible hypothesis on the affinity-enhancing effect of the aryl-aminopyrimidine moieties, we performed molecular dynamics simulations of **7** in complex with galectin-3. Briefly, **7** was placed in the lacNAc binding site of galectin-3 (pdb id ; 1KJL 15) in an orientation where the galactose residue of **7** overlapped with that of lacNAc. The 3,4-dichlorophenyl aglycon of **7** was oriented with the 3-chloro atom towards the Gly182 carbonyl oxygen as observed in a published structure (pdb id ; 6EOL) with a 3,4-dichlorophenyl 3-triazolyl-α-thiogalactoside in complex with galectin-3 (Fig. 1B).^17^ The 4-(4-chlorophenyl)-2-aminopyrimidine group was placed in different conformations and 200 ns molecular dynamics simulations were performed. All starting conformations of **7** converged towards similar stable complex geometries with the aminopyrimidine moiety hydrogen binding to the Asp148 carboxylate and transiently so to Ser237 (Fig. 2). The Arg144 side chain was flexible and sampled conformations interacting with the aminopyrimidine ring, the chlorophenyl ring, or galactose O3. Hence, the aminopyrimidine moiety adopts an interaction mode not seen in published galectin-3 ligand X-ray and neutron complexes (*e.g.* as in Fig. 1A and B). Instead, the aminopyrimidine moiety can replace the water and shortcut the water-mediated Asp148–Arg144 interaction observed in X-ray and neutron diffraction complexes with natural ligand fragments, such as lactose (*e.g.*; 3ZSJ and ; 6EYM, Fig. 1A) or lacNAc (*e.g.*; 1KJL), with direct polar interactions with Asp148 and Ser237. In X-ray complexes with earlier aromatic inhibitor molecules, a benzamide (*e.g.*; 1KJR or ; 4BM8) or a phenyltriazole (*e.g.*; 5E89, ; 6EOL (see Fig. 1B), ; 6QGF and ; 6I74) intersects and bridges a water–Arg144 interaction. The bridging of the Asp148–water–Arg144 interaction by a phenyl triazole (; 6EOL) appears significantly more efficient^17^ with low-nM affinities for galectin-3 (*K*_d_ 37 nM), as compared to that of **7**, but the phenyltriazole also forms more favorable interactions with other galectins which thus leads to lower selectivities. The amino acid motif made up by Arg144, Asp148, and Ser237 in galectin-3 in the proximity of bound galactose O3 and the aminopyrimidine moieties of **1** and **7** is not present in other galectins, which may explain the high selectivity of compounds **1** and **7** for galectin-3.

## Conclusions

Aminopyrimidines **1a–n** are galectin-3 inhibitors with excellent selectivities and affinities down to 83 μM. Evaluation of aminopyrimidines **1a–b** and **1e** affinities for galectin-3 Arg144 mutants suggested that interactions with Arg144 are important. Combining a 3-*O*-aminopyrimidinylmethyl substituent with a 3,4-dichlorophenyl α-thioglycosidic aglycon resulted in a galectin-3 inhibitor **7** with low micromolar affinity and at least 34-fold or better selectivity for galectin-3 over other galectins, including an important about 300-fold selectivity over galectin-1. Molecular dynamics simulations all converged to a geometry of **7** in complex with galectin-3 in which the 2-aminopyrimidine moiety formed stable hydrogen bonds with Asp148 and, to a lesser extent, to Ser237, which can provide an explanation for the affinity enhancements observed. While the affinity of **7** for galectin-3 is lower than those of the recently reported 3,4-dichlorophenyl α-thiogalactosides,^17^ the selectivity over galectin-1 is much more pronounced, which hence would be an advantage in the use of **7** as a galectin-3 inhibitor in biological experiments.

## Experimental

### General procedures

Chemicals were obtained from Sigma-Aldrich unless otherwise stated and used without further purification unless stated in the procedure. NMR spectra were collected on a Bruker Ultrashield Plus/Avance II 400 MHz spectrometer. ^1^H spectra were recorded at 400 MHz and ^13^C spectra at 100 MHz with residual solvent signal as references. All final compounds were purified using preparative HPLC on an Agilent 1260 Infinity system with a SymmetryPrep C18 5 μM 19 × 100 mm column using a gradient (water with 0.1% formic acid and acetonitrile): 0–20 minutes 10–100% acetonitrile, 20–23 minutes 100% acetonitrile. Monitoring and collection was based on UV/VIS absorbance at 210 and 254 nm. Purity analysis was performed using UPLC/MS with UV/VIS detection on a Waters Acquity UPLC + Waters XEVO-G2 system using a Waters Acquity CSH C18, 1.7 μm, 2.1 × 100 mm column. Samples were run using a gradient with water (0.1% formic acid) and acetonitrile and a flow rate of 0.50 mL min^–1^ and a column temperature of 60 °C. Gradient parameters: 0–0.7 min 40% acetonitrile, 0.7–10.0 min 40–99% acetonitrile, 10.0–11.0 min 99% acetonitrile, 11.0–11.1 min 99–40% acetonitrile, 11.1–13 min 40% acetonitrile, 3 or 6 μL injection, detection 190–300 nm. MS parameters: cap voltage 3.0 kV, cone voltage 40 kV, Ext 4, source temp 120 °C, desolvation temp 500 °C, cone gas 50 L h^–1^, desolvation gas (nitrogen) 800 L h^–1^, centroid resolution mode, *m*/*z* interval 50–1200, Lockspray. Calibration: Leu-enkephalin *m*/*z* 556.2771, 0.25 s every 30 s, average 3. For optical rotation measurements, samples were dissolved in an appropriate solvent to a concentration of 2–10 mg mL^–1^. Polarimetry was performed on a PerkinElmer model 341 polarimeter using a sodium lamp and measuring at 589 nM with a 90 mm long 1 mL cell at 20 °C.

### Synthetic procedures

#### Methyl 2,4,6-tri-*O*-acetyl-3-*O*-(4-phenyl-4-oxo-but-2-ynyl)-β-d-galactopyranoside **3a**

Compound **2** (ref. 18) (150 mg, 0.418 mmol), bis(triphenylphosphine)palladium(ii) dichloride (15 mg 0.021 mmol), and copper(i) iodide (12 mg, 0.063 mmol) were dissolved in dry tetrahydrofuran (10 mL) under nitrogen. Triethylamine (78 μL, 0.837 mmol) and benzoyl chloride (73 μL, 0.625 mmol) were added and the mixture was left overnight at room temperature under nitrogen. The mixture was poured into brine (25 mL), which was extracted twice with dichloromethane (25 mL). The extracts were pooled, dried with anhydrous sodium sulfate, filtered and evaporated. The crude was purified using column chromatography (3 : 2 heptane/ethyl acetate) to give **3a** (68 mg, 34%) as a clear and viscous oil. [*α*]20D: 19° (*c* = 0.3861) in acetonitrile. ^1^H NMR (400 MHz in CDCl_3_): 8.20–8.15 (m, 2H), 7.67 (tt. *J* = 7.4 Hz, 1.3 Hz, 1H), 7.57–7.50 (m, 2H), 5.47 (dd, *J* = 3.6 Hz, 0.7 Hz, 1H, H4), 5.14 (dd, *J* = 9.2 Hz, 8.1 Hz, 1H, H2), 4.52 (s, 2H), 4.43 (d, *J* = 8.3 Hz, 1H, H1), 4.26–4.15 (m, 2H), 3.92–3.84 (m, 2H), 3.54 (s, 3H), 2.19 (s, 3H), 2.13 (s, 3H), 2.09 (s, 3H). ^13^C NMR (100 MHz in CDCl_3_): 134.53, 129.64, 128.73, 102.06, 89.11, 70.66, 69.82, 65.28, 61.62, 56.88, 56.69, 20.95, 20.80, 20.72. HRMS: M + NH_4_^+^: 480.1879 found, 480.1870 calculated.

#### Methyl 2,4,6-tri-*O*-acetyl-3-*O*-(4-naphth-2-yl-4-oxo-but-2-ynyl)-β-d-galactopyranoside **3b**

Compound **2** (100 mg, 0.279 mmol), bis(triphenylphosphine)palladium(ii) dichloride (10 mg, 0.014 mmol), copper(i) iodide (8 mg, 0.042 mmol), and 2-naphthoyl chloride (91 mg 0.419 mmol) were dissolved in dry tetrahydrofuran (8 mL) under nitrogen. Triethylamine (78 μL, 0.558 mmol) was added and the mixture was left overnight at room temperature. The mixture was poured into brine (25 mL), which was extracted twice with dichloromethane (25 mL). The extracts were pooled, dried with anhydrous sodium sulfate, filtered, and evaporated. The crude was purified using column chromatography (3 : 2 heptane/ethyl acetate) to give **3b** (52 mg, 36%) as a clear and viscous slightly yellow oil. [*α*]20D: 20° (*c* = 0.3988) in acetonitrile. ^1^H NMR (400 MHz in CDCl_3_): 8.78 (s, 1H), 8.15 (dd, *J* = 8.7 Hz, 1.9 Hz, 1H), 8.08 (d, *J* = 8.4 Hz, 1H), 7.96–7.91 (m, 2H), 7.68 (td, *J* = 7.6 Hz, 1.4 Hz, 1H), 7.62 (td, *J* = 7.6 Hz, 1.3 Hz, 1H), 5.50 (dd, *J* = 3.4 Hz, 0.9 Hz, 1H, H4), 5.19 (dd, *J* = 9.7 Hz, 8.2 Hz, 1H, H2), 4.57 (s, 2H), 4.48 (d, *J* = 7.9 Hz, 1H, H1), 4.26–4.16 (m, 2H), 3.96 (dd, *J* = 10.4 Hz, 3.6 Hz, 1H, H3), 3.91 (td, *J* = 6.8 Hz, 1.1 Hz, 1H, H5), 3.57 (s, 3H), 2.20 (s, 3H), 2.12 (s, 3H), 2.06 (s, 3H). ^13^C NMR (100 MHz in CDCl_3_): 177.12, 170.60, 170.49, 169.76, 136.33, 133.89, 133.10, 132.45, 129.76, 129.34, 128.75, 128.02, 127.19, 123.60, 102.11, 88.97, 84.74, 70.71, 69.89, 65.38, 61.62, 56.92, 56.88, 20.98, 20.83, 20.69. HRMS: M + NH_4_^+^: 530.2026 found, 530.2026 calculated.

#### Methyl 2,4,6-tri-*O*-acetyl-3-*O*-(4-naphth-1-yl-4-oxo-but-2-ynyl)-β-d-galactopyranoside **3c**

Compound **2** (150 mg, 0.418 mmol), bis(triphenylphosphine)palladium(ii) dichloride (15 mg 0.021 mmol), and copper(i) iodide (12 mg, 0.063 mmol) were dissolved in dry tetrahydrofuran (10 mL) under nitrogen. Triethylamine (78 μL, 0.837 mmol) and 1-naphthoyl chloride (94 μL, 0.625 mmol) were added and the mixture was left overnight at room temperature under nitrogen. The mixture was poured into brine (25 mL), which was extracted twice with dichloromethane (25 mL). The extracts were pooled, dried with anhydrous sodium sulfate, filtered and evaporated. The crude was purified using column chromatography (3 : 2 heptane/ethyl acetate) to give **3c** (117 mg, 55%) as a clear and yellow viscous oil. [*α*]20D: 19° (*c* = 0.9426) in acetonitrile. ^1^H NMR (400 MHz, CDCl_3_): 9.22 (dd, *J* = 8.6 Hz, 0.9 Hz, 1H), 8.59 (dd, *J* = 7.3 Hz, 1.2 Hz, 1H), 8.13 (d, *J* = 8.3 Hz, 1H), 7.94 (dt, *J* = 8.2 Hz, 0.7 Hz, 1H), 7.71 (m, 1H), 7.64–7.55 (m, 2H), 5.48 (dd, *J* = 3.5 Hz, 0.9 Hz, 1H, H4), 5.14 (dd, *J* = 9.8 Hz, 8.1 Hz, 1H, H2), 4.53 (s, 2H), 4.43 (d, *J* = 8.0 Hz, 1H, H1), 4.28–4.11 (m, 2H), 3.95–3.86 (m, 2H), 3.54 (s, 3H), 2.19 (s, 3H), 2.12 (s, 3H), 2.07 (s, 3H). ^13^C NMR (100 MHz, CDCl_3_): 178.77, 170.59, 170.50, 169.77, 135.67, 135.15, 133.89, 132.13, 130.70, 129.28, 128.70, 126.97, 124.40, 102.09, 87.70, 85.94, 76.65, 70.67, 69.87, 65.38, 61.65, 56.87, 56.82, 20.97, 20.81, 20.72. HRMS: M + NH_4_^+^: 530.2020 found, 530.2026 calculated.

#### Methyl 2,4,6-tri-*O*-acetyl-3-*O*-[4-(4-fluorophenyl)-4-oxo-but-2-ynyl]-β-d-galactopyranoside **3d**

Compound **2** (150 mg, 0.418 mmol), bis(triphenylphosphine)palladium(ii) dichloride (15 mg 0.021 mmol), and copper(i) iodide (12 mg, 0.063 mmol) were dissolved in dry tetrahydrofuran (10 mL) under nitrogen. Triethylamine (78 μL, 0.837 mmol) and 4-fluorobenzoyl chloride (73 μL, 0.625 mmol) were added and the mixture was left overnight at room temperature under nitrogen. Upon completion, the mixture was poured into brine (25 mL), which was extracted twice with dichloromethane (25 mL). The extracts were pooled, dried with anhydrous sodium sulfate, filtered, and evaporated. The crude was purified using column chromatography (3 : 2 heptane/ethyl acetate) to give **3d** (67 mg, 33%) as a clear and viscous oil. [*α*]20D: 20° (*c* = 0.5217) in acetonitrile. ^1^H NMR (400 MHz in CDCl_3_): 8.22–8.16 (m, 2H), 7.24–7.17 (m, 2H), 5.47 (dd, *J* = 3.6 Hz, 1.0 Hz, 1H, H4), 5.13 (dd, *J* = 9.6 Hz, 8.0 Hz, 1H, H2), 4.50 (s, 2H), 4.42 (d, *J* = 8.1 Hz, 1H, H1), 4.26–4.13 (m, 2H), 3.91–3.82 (m, 2H), 3.54 (s, 3H), 2.18 (s, 3H), 2.12 (s, 3H), 2.08 (s, 3H). ^13^C NMR (100 MHz in CDCl_3_): 175.57, 170.59, 170.49, 169.68, 132.40, 132.30, 116.14, 115.92, 102.03, 89.38, 84.17, 76.81, 70.64, 69.87, 65.34, 61.63, 56.88, 56.77, 20.95, 20.79, 20.71. HRMS: M + NH_4_^+^: 498.1778 found, 498.1775 calculated.

#### Methyl 2,4,6-tri-*O*-acetyl-3-*O*-[4-(4-chlorophenyl)-4-oxo-but-2-ynyl]-β-d-galactopyranoside **3e**

Compound **2** (150 mg, 0.418 mmol), bis(triphenylphosphine)palladium(ii) dichloride (15 mg 0.021 mmol), and copper(i) iodide (12 mg, 0.063 mmol) were dissolved in dry tetrahydrofuran (10 mL) under nitrogen. Triethylamine (78 μL, 0.837 mmol) and 4-chlorobenzoyl chloride (80 μL, 0.625 mmol) were added and the mixture was left overnight at room temperature under nitrogen. Upon completion, the mixture was poured into brine (25 mL), which was extracted twice with dichloromethane (25 mL). The extracts were pooled, dried with anhydrous sodium sulfate, filtered and evaporated. The crude was purified using column chromatography (3 : 2 heptane/ethyl acetate) to give **3e** (48 mg, 23%) as a clear and viscous oil. [*α*]20D: 31° (*c* = 0.1947) in acetonitrile. ^1^H NMR (400 MHz in CDCl_3_): 8.13–8.08 (m, 2H), 7.53–7.49 (m, 2H), 5.46 (dd, *J* = 3.5 Hz, 0.9 Hz, 1H, H4), 5.13 (dd, *J* = 9.4 Hz, 8.0 Hz, 1H, H2), 4.51 (s, 2H), 4.43 (d, *J* = 7.9 Hz, 1H, H1), 4.27–4.16 (m, 2H), 3.91–3.82 (m, 2H), 3.55 (s, 3H), 2.19 (s, 3H), 2.13 (s, 3H), 2.09 (s, 3H). ^13^C NMR (100 MHz in CDCl_3_): 175.89, 170.59, 170.49, 169.70, 141.23, 134.68, 130.93, 129.13, 102.03, 89.65, 76.83, 70.64, 69.87, 65.33, 61.62, 56.89, 56.77, 20.97, 20.80, 20.72. HRMS: M + NH_4_^+^: 514.1470 found, 514.1480 calculated.

#### Methyl 2,4,6-tri-*O*-acetyl-3-*O*-[4-(4-methylphenyl)-4-oxo-but-2-ynyl]-β-d-galactopyranoside **3f**

Compound **2** (150 mg, 0.418 mmol), bis(triphenylphosphine)palladium(ii) dichloride (15 mg 0.021 mmol), and copper(i) iodide (12 mg, 0.063 mmol) were dissolved in dry tetrahydrofuran (10 mL) under nitrogen. Triethylamine (78 μL, 0.837 mmol) and *p*-toluoyl chloride (83 μL, 0.625 mmol) were added and the mixture was left overnight at room temperature under nitrogen. Upon completion, the mixture was poured into brine (25 mL), which was extracted twice with dichloromethane (25 mL). The extracts were pooled, dried with anhydrous sodium sulfate, filtered and evaporated. The crude was purified using column chromatography (3 : 2 heptane/ethyl acetate) to give **3f** (120 mg, 60%) as a clear and viscous oil. [*α*]20D: 29° (*c* = 0.2113) in acetonitrile. ^1^H NMR (400 MHz in CDCl_3_): 8.09–8.04 (m, 2H), 7.35–7.30 (m, 2H), 5.46 (dd, *J* = 3.5 Hz, 1.1 Hz, 1H, H4), 5.14 (dd, *J* = 9.6 Hz, 8.0 Hz, 1H, H2), 4.51 (s, 2H), 4.43 (d, *J* = 8.1 Hz, 1H, H1), 4.27–4.16 (m, 2H), 3.91–3.85 (m, 2H), 3.55 (s, 3H), 2.47 (s, 3H), 2.19 (s, 3H), 2.13 (s, 3H), 2.09 (s, 3H). ^13^C NMR (100 MHz in CDCl_3_): 176.89, 170.57, 170.48, 169.76, 145.76, 134.03, 129.78, 129.44, 102.06, 88.56, 84.61, 76.59, 70.66, 69.82, 65.29, 61.62, 56.86, 56.70, 21.87, 20.97, 20.81, 20.72. HRMS: M + NH_4_^+^: 494.2026 found, 494.2026 calculated.

#### Methyl 2,4,6-tri-*O*-acetyl-3-*O*-[4-(3-fluorophenyl)-4-oxo-but-2-ynyl]-β-d-galactopyranoside **3g**

Compound **2** (150 mg, 0.418 mmol), bis(triphenylphosphine)palladium(ii) dichloride (15 mg 0.021 mmol), and copper(i) iodide (12 mg, 0.063 mmol) were dissolved in dry tetrahydrofuran (10 mL) under nitrogen. Triethylamine (78 μL, 0.837 mmol) and 3-fluorobenzoyl chloride (76 μL, 0.625 mmol) were added and the mixture was left overnight at room temperature under nitrogen. The mixture was poured into brine (25 mL), which was extracted twice with dichloromethane (25 mL). The extracts were pooled, dried with anhydrous sodium sulfate, filtered and evaporated. The crude was purified using column chromatography (3 : 2 heptane/ethyl acetate) to give **3g** (22 mg, 38%) as a clear and viscous oil. [*α*]20D: 18° (*c* = 0.7391) in acetonitrile. ^1^H NMR (400 MHz in CDCl_3_): 7.97 (dt, 7.8 Hz, 1.6 Hz, 1.2 Hz, 1H), 7.84 (ddd, *J* = 9.0 Hz, 2.6 Hz, 1.6 Hz, 1H), 7.52 (m, 1H), 7.37 (tdd, *J* = 8.2 Hz, 2.7 Hz, 1.0 Hz, 1H), 5.46 (dd, *J* = 3.6 Hz, 0.9 Hz, 1H, H4), 5.14 (dd, *J* = 9.4 Hz, 8.1 Hz, 1H, H2), 4.52 (s, 2H), 4.43 (d, *J* = 8.1 Hz, 1H, H1), 4.27–4.12 (m, 2H), 3.93–3.84 (m, 2H), 3.54 (s, 3H), 2.19 (s, 3H), 2.12 (s, 3H), 2.09 (s, 3H). ^13^C NMR (100 MHz, CDCl_3_): 175.82, 170.61, 170.51, 169.71, 164.01, 161.55, 130.55, 125.35, 121.59, 116.18, 102.25, 89.87, 84.34, 70.67, 69.82, 65.25, 61.67, 56.93, 56.66, 20.90, 20.79, 20.72. HRMS: M + NH_4_^+^: 498.1780 found, 498.1775 calculated.

#### Methyl 2,4,6-tri-*O*-acetyl-3-*O*-[4-(3-chlorophenyl)-4-oxo-but-2-ynyl]-β-d-galactopyranoside **3h**

Compound **2** (150 mg, 0.418 mmol), bis(triphenylphosphine)palladium(ii) dichloride (15 mg 0.021 mmol), and copper(i) iodide (12 mg, 0.063 mmol) were dissolved in dry tetrahydrofuran (10 mL) under nitrogen. Triethylamine (78 μL, 0.837 mmol) and 3-chlorobenzoyl chloride (80 μL, 0.625 mmol) were added and the mixture was left overnight at room temperature under nitrogen. The mixture was poured into brine (25 mL), which was extracted twice with dichloromethane (25 mL). The extracts were pooled, dried with anhydrous sodium sulfate, filtered and evaporated. The crude was purified using column chromatography (3 : 2 heptane/ethyl acetate) to give **3h** (52 mg, 25%) as a clear and viscous oil. [*α*]20D: 21° (*c* = 0.3344) in acetonitrile. ^1^H NMR (400 MHz, CDCl_3_): 8.14 (t, *J* = 1.8 Hz, 1H), 8.05 (dt, *J* = 7.6 Hz, 1.3 Hz, 1H, 1H), 7.64 (dq, *J* = 8.0 Hz, 1.0 Hz, 1H), 7.49 (t, *J* = 7.8 Hz, 1H), 5.46 (dd, *J* = 3.5 Hz, 0.9 Hz, 1H, H4), 5.14 (dd, *J* = 10.1 Hz, 8.0 Hz, 1H, H2), 4.52 (s, 2H), 4.45 (d, *J* = 8.0 Hz, 1H, H1), 4.28–4.12 (m, 2H), 3.93–3.85 (m, 2H), 3.55 (s, 3H), 2.19 (s, 3H), 2.12 (s, 3H), 2.09 (s, 3H). ^13^C NMR (100 MHz, CDCl_3_): 175.75, 170.59, 170.49, 169.70, 137.78, 135.07, 134.43, 130.13, 129.59, 127.55, 102.09, 89.99, 84.11, 70.64, 69.77, 65.21, 61.62, 56.92, 56.66, 20.92, 20.80, 20.73. HRMS: M + NH_4_^+^: 514.1486 found, 514.1480 calculated.

#### Methyl 2,4,6-tri-*O*-acetyl-3-*O*-[4-(3-methylphenyl)-4-oxo-but-2-ynyl]-β-d-galactopyranoside **3i**

Compound **2** (150 mg, 0.418 mmol), bis(triphenylphosphine)palladium(ii) dichloride (15 mg 0.021 mmol), and copper(i) iodide (12 mg, 0.063 mmol) were dissolved in dry tetrahydrofuran (10 mL) under nitrogen. Triethylamine (78 μL, 0.837 mmol) and *m*-toluoyl chloride (83 μL, 0.625 mmol) were added and the mixture was left overnight at room temperature under nitrogen. Upon completion, the mixture was poured into brine (25 mL), which was extracted twice with dichloromethane (25 mL). The extracts were pooled, dried with anhydrous sodium sulfate, filtered and evaporated. The crude was purified using column chromatography (3 : 2 heptane/ethyl acetate) to give **3i** (104 mg, 525) as a clear and viscous oil. [*α*]20D: 21° (*c* = 0.8791) in acetonitrile. ^1^H NMR (400 MHz, CDCl_3_): 8.00–7.95 (m, 2H), 7.51–7.38 (m, 2H), 5.46 (dd, *J* = 3.6 Hz, 1.0 Hz, 1H, H4), 5.14 (dd, *J* = 9.1 Hz, 8.1 Hz, 1H, H2), 4.51 (s, 2H), 4.44 (d, *J* = 8.1 Hz, 1H, H1), 4.28–4.12 (m, 2H), 3.93–3.85 (m, 2H), 3.54 (s, 3H), 2.46 (s, 3H), 2.19 (s, 3H), 2.11 (s, 3H), 2.09 (s, 3H). ^13^C NMR (100 MHz, CDCl_3_): 177.40, 170.58, 170.49, 169.75, 138.66, 136.32, 135.38, 129.86, 128.62, 127.10, 102.07, 88.84, 84.65, 77.23, 76.61, 70.61, 69.81, 65.28, 61.63, 56.88, 56.70, 21.25, 20.93, 20.80, 20.72. HRMS: M + NH_4_^+^: 494.2035 found, 494.2026 calculated.

#### Methyl 2,4,6-tri-*O*-acetyl-3-*O*-[4-(4-bromophenyl)-4-oxo-but-2-ynyl]-β-d-galactopyranoside **3j**

Compound **2** (100 mg, 0.279 mmol), bis(triphenylphosphine)palladium(ii) dichloride (10 mg, 0.014 mmol), copper(i) iodide (8 mg, 0.042 mmol) and 4-bromobenzoyl chloride (91 mg, 0.419 mmol) were dissolved in dry tetrahydrofuran (8 mL) under nitrogen. Triethylamine (78 μL, 0.558 mmol) was added and the mixture was left overnight. Upon completion, the mixture was poured into brine (25 mL), which was extracted twice with dichloromethane (25 mL). The extracts were pooled, dried with anhydrous sodium sulfate, filtered and evaporated. The crude was purified using column chromatography (3 : 2 heptane/ethyl acetate) to give **3j** (22 mg, 15%) as a clear and viscous slightly yellow oil. [*α*]20D: 24° (*c* = 0.2037) in acetonitrile. ^1^H NMR (400 MHz, CDCl_3_): 8.04–8.00 (m, 2H), 7.70–7.66 (m, 2H), 5.46 (dd, *J* = 3.5 Hz, 1.0 Hz, 1H, H4), 5.13 (dd, *J* = 9.9 Hz, 7.9 Hz, 1H, H2), 4.51 (s, 2H), 4.42 (d, *J* = 8.0 Hz, 1H, H1), 4.27–4.13 (m, 2H), 3.92–3.82 (m, 2H), 3.55 (s, 3H), 2.19 (s, 3H), 2.13 (s, 3H), 2.09 (s, 3H). ^13^C NMR (100 MHz, CDCl_3_): 176.07, 170.58, 170.50, 169.66, 135.07, 132.13, 130.97, 130.09, 102.02, 89.70, 84.12, 76.84, 70.64, 69.87, 65.33, 61.61, 56.89, 56.78, 20.97, 20.80, 20.72. HRMS: M + NH_4_^+^: 558.0961 found, 558.0975 calculated.

#### Methyl 2,4,6-tri-*O*-acetyl-3-*O*-[4-(4-tertbutylphenyl)-4-oxo-but-2-ynyl]-β-d-galactopyranoside **3k**

Compound **2** (150 mg, 0.418 mmol), bis(triphenylphosphine)palladium(ii) dichloride (15 mg 0.021 mmol), and copper(i) iodide (12 mg, 0.063 mmol) were dissolved in dry tetrahydrofuran (10 mL) under nitrogen. Triethylamine (78 μL, 0.837 mmol) and *p*-toluoyl chloride (122 μL, 0.625 mmol) were added and the mixture was left overnight at room temperature under nitrogen. Upon completion, the mixture was poured into brine (25 mL), which was extracted twice with dichloromethane (25 mL). The extracts were pooled, dried with anhydrous sodium sulfate, filtered and evaporated. The crude was purified using column chromatography (3 : 2 heptane/ethyl acetate) to give **3k** (115 mg, 53%) as a clear and viscous oil. [*α*]20D: 24° (*c* = 0.3964) in acetonitrile. ^1^H NMR (400 MHz, CDCl_3_): 8.12–8.07 (m, 2H), 7.57–7.51 (m, 2H), 5.46 (dd, *J* = 3.5 Hz, 0.9 Hz, 1H, H4), 5.14 (dd, *J* = 9.9 Hz, 8.0 Hz, 1H, H2), 4.51 (s, 2H), 4.44 (d, *J* = 8.0 Hz, 1H, H1), 4.28–4.15 (m, 2H), 3.93–3.85 (m, 2H), 3.55 (s, 3H), 2.19 (s, 3H), 2.13 (s, 3H), 2.09 (s, 3H), 1.38 (s, 9H). ^13^C NMR (100 MHz, CDCl_3_): 170.57, 170.48, 169.77, 158.65, 133.91, 129.63, 125.71, 102.06, 88.53, 84.64, 76.60, 70.66, 69.82, 65.30, 61.63, 56.87, 56.70, 31.05, 20.96, 20.81, 20.73. HRMS: M + H^+^: 541.0692 found, 514.0709 calculated.

#### Methyl 2,4,6-tri-*O*-acetyl-3-*O*-[4-(4-biphenyl)-4-oxo-but-2-ynyl]-β-d-galactopyranoside **3l**

Compound **2** (100 mg, 0.279 mmol), bis(triphenylphosphine)palladium(ii) dichloride (10 mg, 0.014 mmol), copper(i) iodide (8 mg, 0.042 mmol) and biphenyl-4-carbonyl chloride (91 mg, 0.419 mmol) was dissolved in dry tetrahydrofuran (8 mL) under nitrogen. Triethylamine (78 μL, 0.558 mmol) was added and the mixture was left overnight at room temperature. Upon completion, the mixture was poured into brine (25 mL), which was extracted twice with dichloromethane (25 mL). The extracts were pooled, dried with anhydrous sodium sulfate, filtered and evaporated. The crude was purified using column chromatography (3 : 2 heptane/ethyl acetate) to give **3l** (72 mg, 48%) as a clear, slightly yellow viscous oil. [*α*]20D: 26° (*c* = 0.2963) in acetonitrile. ^1^H NMR (400 MHz, CDCl_3_): 8.26–8.21 (m, 2H), 7.78–7.72 (m, 2H), 7.69–7.64 (m, 2H), 7.55–7.49 (m, 2H), 7.45 (tt, *J* = 7.3 Hz, 2.4 Hz, 1H), 5.48 (dd, *J* = 3.6 Hz, 1.0 Hz, 1H, H4), 5.15 (dd, *J* = 9.4 Hz, 8.1 Hz, 1H, H2), 4.54 (s, 2H), 4.44 (d, *J* = 8.4 Hz, 1H, H1), 4.28–4.16 (m, 2H), 3.94–3.85 (m, 2H), 3.55 (s, 3H), 2.20 (s, 3H), 2.15 (s, 3H), 2.09 (s, 3H). ^13^C NMR (100 MHz, CDCl_3_): 176.76, 170.59, 170.48, 169.75, 147.29, 139.56, 135.13, 130.24, 129.08, 128.62, 127.36, 102.06, 89.03, 84.59, 70.67, 69.85, 65.32, 61.63, 56.88, 56.75, 21.00, 20.82, 20.72. HRMS: M + H^+^: 539.1922 found, 539.1917 calculated.

#### Methyl 2,4,6-tri-*O*-acetyl-3-*O*-[4-(3-biphenyl)-4-oxo-but-2-ynyl]-β-d-galactopyranoside **3m**

Compound **2** (100 mg, 0.279 mmol), bis(triphenylphosphine)palladium(ii) dichloride (10 mg, 0.014 mmol), copper(i) iodide (8 mg, 0.042 mmol) and biphenyl-3-carbonyl chloride (78 μL, 0.419 mmol) was dissolved in dry tetrahydrofuran (8 mL) under nitrogen. Triethylamine (78 μL, 0.558 mmol) was added and the mixture was left overnight at room temperature. Upon completion, the mixture was poured into brine (25 mL), which was extracted twice with dichloromethane (25 mL). The extracts were pooled, dried with anhydrous sodium sulfate, filtered and evaporated. The crude was purified using column chromatography (3 : 2 heptane/ethyl acetate) to give **3m** (23 mg, 15%) as a clear and viscous oil. [*α*]20D: 17° (*c* = 0.0784) in acetonitrile. ^1^H NMR (400 MHz, CDCl_3_): 8.36 (t, *J* = 1.7 Hz, 1H), 8.16 (ddd, *J* = 7.7 Hz, 1.8 Hz, 1.2 Hz, 1H), 7.89 (ddd, *J* = 7.7 Hz, 2.0 Hz, 1.2 Hz, 1H), 7.68–7.58 (m, 3H), 7.55–7.48 (m, 2H), 7.43 (tt, *J* = 7.4 Hz, 1.3 Hz, 1H), 5.47 (dd, *J* = 3.4 Hz, 1.0 Hz, 1H, H4), 5.13 (dd, *J* = 9.3 Hz, 8.1 Hz, 1H, H2), 4.53 (s, 2H), 4.39 (d, *J* = 8.1 Hz, 1H, H1), 4.27–4.15 (m, 2H), 3.92–3.85 (m, 2H), 3.55 (s, 3H), 2.18 (s, 3H), 2.09 (s, 3H), 2.08 (s, 3H). ^13^C NMR (100 MHz, CDCl_3_): 177.20, 170.58, 170.48, 169.75, 136.79, 133.17, 129.22, 129.05, 128.58, 128.09, 127.96, 127.21, 102.01, 89.28, 84.57, 70.64, 69.77, 65.23, 61.62, 56.83, 56.66, 20.88, 20.79, 20.73. HRMS: M + H^+^: 539.1926 found, 539.1917 calculated.

#### Methyl 2,4,6-tri-*O*-acetyl-3-*O*-[4-(2-fluorophenyl)-4-oxo-but-2-ynyl]-β-d-galactopyranoside **3n**

Compound **2** (100 mg, 0.279 mmol), bis(triphenylphosphine)palladium(ii) dichloride (10 mg, 0.014 mmol), copper(i) iodide (8 mg, 0.042 mmol) and biphenyl-4-carbonyl chloride (91 mg, 0.419 mmol) was dissolved in dry tetrahydrofuran (8 mL) under nitrogen. Triethylamine (78 μL, 0.558 mmol) was added and the mixture was left overnight at room temperature. Upon completion, the mixture was poured into brine (25 mL), which was extracted twice with dichloromethane (25 mL). The extracts were pooled, dried with anhydrous sodium sulfate, filtered and evaporated. The crude was purified using column chromatography (3 : 2 heptane/ethyl acetate) to give **3n** (23 mg, 17%) as a clear and viscous oil. [*α*]20D: 30° (*c* = 0.2001) in acetonitrile. ^1^H NMR (400 MHz, CDCl_3_): 8.05 (td, *J* = 7.7 Hz, 1.8 Hz, 1H), 7.66–7.59 (m, 1H), 7.30 (td, *J* = 7.6 Hz, 1.0 Hz, 1H), 7.20 (ddd, *J* = 11.1 Hz, 8.3 Hz, 0.9 Hz, 1H), 5.45 (dd, *J* = 3.4 Hz, 1.0 Hz, 1H, H4), 5.12 (dd, *J* = 10.3 Hz, 8.0 Hz, 1H, H2), 4.49 (d, *J* = 1.5 Hz, 2H), 4.43 (d, *J* = 8.0 Hz, 1H, H1), 4.28–4.15 (m, 2H), 3.93–3.85 (m, 2H), 3.54 (s, 3H), 2.17 (s, 3H), 2.10 (s, 3H), 2.09 (s, 3H). ^13^C NMR (100 MHz, CDCl_3_): 173.45, 170.55, 170.49, 169.77, 163.43, 160.82, 136.06, 135.97, 131.91, 125.08, 124.39, 124.35, 117.24, 117.03, 102.04, 89.04, 85.90, 76.47, 70.65, 69.78, 65.33, 61.65, 56.85, 56.64, 20.89, 20.80, 20.74. HRMS: M + NH_4_^+^: 498.1774 found, 498.1775 calculated.

#### Methyl 3-((2-amino-6-phenyl-pyrimidin-4-yl)methyl)-β-d-galactopyranoside **1a**

Compound **3a** (68 mg, 0.148 mmol) was dissolved in dry tetrahydrofuran (5 mL). Guanidinium hydrochloride (21 mg, 0.221 mmol) and cesium carbonate (72 mg, 0.221 mmol) were added and the reaction mixture was refluxed overnight under nitrogen. The mixture was filtered through a short silica plug using ethyl acetate and then evaporated to give 53 mg of crude. The crude was dissolved in dry methanol (5 mL) with sodium methoxide (23 mg, 0.417 mmol) under nitrogen, and the reaction was left at room temperature for three hours. Amberlite IR120 (H^+^) ion exchange resin was added to bring the mixture to pH 7, after which the mixture was filtered and evaporated. The crude was purified using column chromatography (7 : 1 DCM/methanol) followed by preparative HPLC (gradient from 90% water with 0.1% formic acid/10% acetonitrile to 100% acetonitrile for 10 minutes) to give **1a** (5 mg, 9%) as a white solid. [*α*]20D: 31° (*c* = 0.2619) in methanol. ^1^H NMR (400 MHz, MeOD-d4): 8.13–8.08 (m, 2H), 7.53–7.46 (m, 4H), 4.74 (d, *J* = 15.2 Hz, 1H), 4.67 (d, *J* = 15.2 Hz, 1H), 4.22 (d, *J* = 7.8 Hz, 1H, H1), 4.15 (dd, *J* = 3.1 Hz, 0.8 Hz, 1H, H4), 3.85–3.72 (m, 3H), 3.59–3.52 (m, 4H), 3.47 (dd, *J* = 9.6 Hz, 3.4 Hz, 1H, H3). ^13^C NMR (100 MHz, deuterated acetone): 163.14, 161.44, 137.33, 130.58, 128.56, 127.07, 104.75, 103.13, 83.04, 75.01, 70.31, 70.21, 65.74, 61.48, 55.67. HRMS: M + H^+^: 378.1662 found, 378.1665 calculated. Purity HPLC-MS, UV/VIS detection: 99.1%.

#### Methyl 3-((2-amino-6-(2-naphthyl)-pyrimidin-4-yl)methyl)-β-d-galactopyranoside **1b**

Compound **3b** (44 mg, 0.087 mmol) was dissolved in dry tetrahydrofuran (5 mL). Guanidinium hydrochloride (12 mg, 0.130 mmol) and cesium carbonate (42 mg, 0.130 mmol) were added and the reaction mixture refluxed overnight under nitrogen. The mixture was filtered through a short silica plug using ethyl acetate and then evaporated to give 13 mg of crude. The crude was dissolved in dry methanol (5 mL) with sodium methoxide (6 mg, 0.106 mmol) under nitrogen, and the reaction was left at room temperature for three hours. Amberlite IR120 (H^+^) ion exchange resin was added to bring the mixture to pH 7, after which the mixture was filtered and evaporated. The crude was purified using column chromatography (7 : 1 DCM/methanol) followed by preparative HPLC (gradient from 90% water with 0.1% formic acid/10% acetonitrile to 100% acetonitrile for 10 minutes) to give **1b** (4 mg, 11%) as a white solid. [*α*]20D: 23° (*c* = 0.4195) in methanol. ^1^H NMR (400 MHz, MeOD-d4): 8.65 (s, 1H), 8.21 (dd, *J* = 8.6 Hz, 1.7 Hz, 1H), 8.04–7.90 (m, 3H), 7.63 (s, 1H), 7.61–7.53 (m, 2H), 4.77 (d, *J* = 15.0 Hz, 1H), 4.69 (d, *J* = 15.0 Hz, 1H), 4.23 (d, *J* = 7.7 Hz, 1H, H1), 4.17 (d, *J* = 3.1 Hz, 1H, H4), 3.87–3.75 (m, 3H), 3.60–3.53 (m, 4H), 3.50 (dd, *J* = 9.8 Hz, 3.6 Hz, 1H, H3). ^13^C NMR (100 MHz, MeOD-d4): 166.05, 134.68, 134.39, 133.24, 128.62, 128.00, 127.32, 127.20, 127.03, 126.20, 123.86, 104.52, 103.88, 82.63, 75.00, 70.23, 70.09, 65.41, 61.07, 55.87. HRMS: M + H^+^: 428.1827 found, 428.1822 calculated. Purity HPLC-MS, UV/VIS detection: 99.1%.

#### Methyl 3-((2-amino-6-(1-naphthyl)-pyrimidin-4-yl)methyl)-β-d-galactopyranoside **1c**

Compound **3c** (108 mg, 0.211 mmol) was dissolved in dry tetrahydrofuran (6 mL). Guanidinium hydrochloride (30 mg, 0.316 mmol) and cesium carbonate (103 mg, 0.316 mmol) were added and the reaction mixture refluxed overnight under nitrogen. The mixture was filtered through a short silica plug using ethyl acetate and then evaporated to give 41 mg of crude. The crude was dissolved in dry methanol (5 mL) with sodium methoxide (18 mg, 0.333 mmol) under nitrogen, and the reaction was left at room temperature for three hours. Amberlite IR120 (H^+^) ion exchange resin was added to bring the mixture to pH 7, after which the mixture was filtered and evaporated. The crude was purified using column chromatography (7 : 1 DCM/methanol) followed by preparative HPLC (gradient from 90% water with 0.1% formic acid/10% acetonitrile to 100% acetonitrile for 10 minutes) to give **1c** (24 mg, 27%) as a yellow solid. [*α*]20D: 18° (*c* = 2.033) in methanol. ^1^H NMR (400 MHz, MeOD-d4): 8.14–8.09 (m, 1H), 7.98–7.89 (m, 2H), 7.61 (dd, *J* = 7.1 Hz, 1.4 Hz, 1H), 7.57–7.45 (m, 3H), 7.16 (s, 1H), 4.74 (d, *J* = 15.8 Hz, 1H), 4.66 (d, *J* = 15.8 Hz, 1H), 4.16 (d, *J* = 7.9 Hz, 1H, H1), 4.09 (dd, *J* = 3.2 Hz, 0.8 Hz, 1H, H4), 3.80–3.66 (m, 3H), 3.54–3.47 (m, 4H), 3.43 (dd, *J* = 9.8 Hz, 3.2 Hz, 1H, H3). ^13^C NMR (100 MHz, MeOD-d4): 167.75, 166.82, 161.71, 161.22, 134.82, 132.38, 128.93, 128.06, 126.56, 125.34, 124.86, 124.31, 123.49, 123.31, 106.98, 102.94, 81.12, 73.44, 68.75, 68.66, 63.97, 59.51, 54.31. HRMS: M + H^+^: 428.1816 found, 428.1822 calculated. Purity HPLC-MS, UV/VIS detection: 98.6%.

#### Methyl 3-((2-amino-6-(4-fluorophenyl)-pyrimidin-4-yl)methyl)-β-d-galactopyranoside **1d**

Compound **3d** (65 mg, 0.135 mmol) was dissolved in dry tetrahydrofuran (5 mL). Guanidinium hydrochloride (19 mg, 0.203 mmol) and cesium carbonate (66 mg, 0.203 mmol) were added and the reaction mixture was refluxed overnight under nitrogen. The mixture was filtered through a short silica plug using ethyl acetate and then evaporated to give 14 mg of crude. The crude was dissolved in dry methanol (5 mL) with sodium methoxide (7 mg, 0.131 mmol) under nitrogen, and the reaction was left at room temperature for three hours. Amberlite IR120 (H^+^) ion exchange resin was added to bring the mixture to pH 7, after which the mixture was filtered and evaporated. The crude was purified using column chromatography (7 : 1 DCM/methanol) followed by preparative HPLC (gradient from 90% water with 0.1% formic acid/10% acetonitrile to 100% acetonitrile for 10 minutes) to give **1d** (6 mg, 11%) as a white solid. [*α*]20D: 26° (*c* = 0.3267) in methanol. ^1^H NMR (400 MHz, MeOD-d4): 8.21–8.14 (m, 2H), 7.47 (s, 1H), 7.27–7.19 (m, 2H), 4.73 (d, *J* = 15.3 Hz, 1H), 4.66 (d, *J* = 15.3 Hz, 1H), 4.22 (d, *J* = 8.1 Hz, 1H, H1), 4.14 (dd, *J* = 3.2 Hz, 0.7 Hz, 1H, H4), 3.85–3.72 (m, 3H), 3.59–3.51 (m, 4H), 3.46 (dd, *J* = 9.7 Hz, 3.2 Hz, 1H, H3). ^13^C NMR (100 MHz, deuterated acetone): 170.06, 163.75, 163.62, 129.29, 129.20, 115.43, 115.21, 104.75, 102.88, 82.93, 75.00, 70.35, 70.29, 65.70, 61.50, 55.68. HRMS: M + H^+^: 396.1575 found, 396.1571 calculated. Purity HPLC-MS, UV/VIS detection: 99.2%.

#### Methyl 3-((2-amino-6-(4-chlorophenyl)-pyrimidin-4-yl)methyl)-β-d-galactopyranoside **1e**

Compound **3e** (48 mg, 0.097 mmol) was dissolved in dry tetrahydrofuran (5 mL). Guanidinium hydrochloride (14 mg, 0.145 mmol), and cesium carbonate (47 mg, 0.145 mmol) were added and the reaction mixture refluxed overnight under nitrogen. The mixture was filtered through a short silica plug using ethyl acetate and then evaporated to give 8 mg of crude. The crude was dissolved in dry methanol (5 mL) with sodium methoxide (3 mg, 0.056 mmol) under nitrogen, and the reaction was left at room temperature for three hours. Amberlite IR120 (H^+^) ion exchange resin was added to bring the mixture to pH 7, after which the mixture was filtered and evaporated. The crude was purified using column chromatography (7 : 1 DCM/methanol) followed by preparative HPLC (gradient from 90% water with 0.1% formic acid/10% acetonitrile to 100% acetonitrile for 10 minutes) to give **1e** (9 mg, 23%) as a white solid. [*α*]20D: 28° (*c* = 0.3064) in methanol. ^1^H NMR (400 MHz, MeOD-d4): 8.14–8.10 (m, 2H), 7.53–7.47 (m, 3H), 4.73 (d, *J* = 15.2 Hz, 1H), 4.66 (d, *J* = 15.2 Hz, 1H), 4.21 (d, *J* = 8.4 Hz, 1H, H1), 4.14 (dd, *J* = 3.2 Hz, 0.8 Hz, 1H, H4), 3.84–3.72 (m, 3H), 3.59–3.51 (m, 4H), 3.46 (dd, *J* = 9.4 Hz, 3.2 Hz, 1H, H3), 2.42 (s, 3H). ^13^C NMR (100 MHz, deuterated acetone): 170.28, 163.57, 136.36, 135.86, 128.66, 128.62, 104.75, 103.02, 82.90, 74.99, 70.38, 70.28, 65.66, 61.43, 55.69. HRMS: M + H^+^: 412.1271 found, 412.1275 calculated. Purity HPLC-MS, UV/VIS detection: 98.4%.

#### Methyl 3-((2-amino-6-(4-methylphenyl)-pyrimidin-4-yl)methyl)-β-d-galactopyranoside **1f**

Compound **3f** (120 mg, 0.252 mmol) was dissolved in dry tetrahydrofuran (8 mL). Guanidinium hydrochloride (36 mg, 0.378 mmol), and cesium carbonate (123 mg, 0.378 mmol) were added and the reaction mixture was refluxed overnight under nitrogen. The mixture was filtered through a short silica plug using ethyl acetate and then evaporated to give 50 mg of crude. The crude was dissolved in dry methanol (5 mL) with sodium methoxide (12 mg, 0.217 mmol) under nitrogen, and the reaction was left at room temperature for three hours. Amberlite IR120 (H^+^) ion exchange resin was added to bring the mixture to pH 7, after which the mixture was filtered and evaporated. The crude was purified using column chromatography (7 : 1 DCM/methanol) followed by preparative HPLC (gradient from 90% water with 0.1% formic acid/10% acetonitrile to 100% acetonitrile for 10 minutes) to give **1f** (14 mg, 14%) as a white solid. [*α*]20D: 33° (*c* = 0.2051) in methanol. ^1^H NMR (400 MHz, MeOD-d4): 8.02–7.98 (m, 2H), 7.44 (s, 1H), 7.34–7.29 (m, 2H), 4.72 (d, *J* = 15.0 Hz, 1H), 4.65 (d, *J* = 15.0 Hz, 1H), 4.22 (d, *J* = 8.2 Hz, 1H, H1), 4.14 (dd, *J* = 3.2 Hz, 0.8 Hz, 1H, H4), 3.85–3.72 (m, 3H), 3.59–3.51 (m, 4H), 3.46 (dd, *J* = 9.6 Hz, 3.2 Hz, 1H, H3), 2.42 (s, 3H). ^13^C NMR (100 MHz, MeOD-d4): 168.80, 141.01, 134.31, 129.00, 126.93, 104.51, 103.35, 82.61, 74.99, 70.20, 70.07, 65.39, 61.07, 55.83, 19.99. HRMS: M + H^+^: 392.1817 found, 392.1822 calculated. Purity HPLC-MS, UV/VIS detection: 97.9%.

#### Methyl 3-((2-amino-6-(3-fluorophenyl)-pyrimidin-4-yl)methyl)-β-d-galactopyranoside **1g**

Compound **3g** (68 mg, 0.141 mol) was dissolved in dry tetrahydrofuran (5 mL). Guanidinium hydrochloride (68 mg, 0.142 mmol) and cesium carbonate (69 mg, 0.142 mmol) were added and the reaction mixture was refluxed overnight under nitrogen. The mixture was filtered through a short silica plug using ethyl acetate and then evaporated to give 22 mg of crude. The crude was dissolved in dry methanol (3 mL) with sodium methoxide (10 mg, 0.186 mmol) under nitrogen, and the reaction was left at room temperature for three hours. Amberlite IR120 (H^+^) ion exchange resin was added to bring the mixture to pH 7, after which the mixture was filtered and evaporated. The crude was purified using column chromatography (5 : 1 DCM/methanol) followed by preparative HPLC (gradient from 90% water with 0.1% formic acid/10% acetonitrile to 100% acetonitrile for 10 minutes) to give **1g** (16 mg, 29%) as a white solid. [*α*]20D: 32° (*c* = 0.2381) in methanol. ^1^H NMR (400 MHz, MeOD-d4): 7.94 (ddd, *J* = 7.9 Hz, 1.7 Hz, 0.9 Hz, 1H), 7.88 (ddd, *J* = 10.4 Hz, 2.7 Hz, 1.6 Hz, 1H), 7.55–7.47 (m, 2H), 7.24 (tdd, *J* = 8.4 Hz, 2.7 Hz, 0.8 Hz, 1H), 4.73 (d, *J* = 15.2 Hz, 1H), 4.66 (d, *J* = 15.2 Hz, 1H), 4.22 (d, *J* = 7.8 Hz, 1H, H1), 4.15 (dd, *J* = 3.2 Hz, 0.8 Hz, 1H, H4), 3.85–3.72 (m, 3H), 3.59–3.51 (m, 4H), 3.46 (dd, *J* = 9.7 Hz, 3.3 Hz, 1H, H3). ^13^C NMR (100 MHz, MeOD-d4): 169.59, 130.17, 130.08, 122.72, 117.05, 116.84, 113.61, 113.38, 104.51, 103.53, 82.57, 74.99, 70.20, 70.10, 65.38, 61.06, 55.85. HRMS: M + H^+^: 396.1570 found, 396.1571 calculated. Purity HPLC-MS, UV/VIS detection: 98.1%.

#### Methyl 3-((2-amino-6-(3-chlorophenyl)-pyrimidin-4-yl)methyl)-β-d-galactopyranoside **1h**

Compound **3h** (47 mg, 0.094 mmol) was dissolved in dry tetrahydrofuran (6 mL). Guanidinium hydrochloride (14 mg, 0.141 mmol) and cesium carbonate (46 mg, 0.141 mmol) were added and the reaction mixture was refluxed overnight under nitrogen. The mixture was filtered through a short silica plug using ethyl acetate and then evaporated to give 27 mg of crude. The crude was dissolved in dry methanol (3 mL) with sodium methoxide (12 mg, 0.227 mmol) under nitrogen, and the reaction was left at room temperature for three hours. Amberlite IR120 (H^+^) ion exchange resin was added to bring the mixture to pH 7, after which the mixture was filtered and evaporated. The crude was purified using column chromatography (7 : 1 DCM/methanol) followed by preparative HPLC (gradient from 90% water with 0.1% formic acid/10% acetonitrile to 100% acetonitrile for 10 minutes) to give **1h** (3 mg, 9%) as a white solid. [*α*]20D: 38° (*c* = 0.0799) in methanol. ^1^H NMR (400 MHz, MeOD-d4): 8.16 (t, *J* = 3.0 Hz, 1H), 8.04 (dt, *J* = 10.9 Hz, 3.0 Hz, 1H), 7.54–7.46 (m, 3H), 4.73 (d, *J* = 15.3 Hz, 1H), 4.67 (d, *J* = 15.3 Hz, 1H), 4.22 (d, *J* = 7.9 Hz, 1H, H1), 4.15 (d, *J* = 3.0 Hz, 1H, H4), 3.85–3.72 (m, 3H), 3.59–3.51 (m, 4H), 3.47 (dd, *J* = 9.4 Hz, 3.3 Hz, 1H, H3). ^13^C NMR (100 MHz, MeOD-d4): 164.36, 139.30, 130.13, 129.88, 126.83, 125.25, 104.51, 103.52, 82.58, 75.00, 70.20, 65.39, 61.06, 55.84. HRMS: M + H^+^: 412.1266 found, 412.1275 calculated. Purity HPLC-MS, UV/VIS detection: 98.4%.

#### Methyl 3-((2-amino-6-(3-methylphenyl)-pyrimidin-4-yl)methyl)-β-d-galactopyranoside **1i**

Compound **3i** (78 mg, 0.164 mmol) was dissolved in dry tetrahydrofuran (6 mL). Guanidinium hydrochloride (23 mg, 0.246 mmol) and cesium carbonate (80 mg, 0.246 mmol) were added and the reaction mixture refluxed overnight under nitrogen. The mixture was filtered through a short silica plug using ethyl acetate and then evaporated to give a crude product. The crude was dissolved in dry methanol (5 mL) with sodium methoxide (40 mg, 0.738 mmol) under nitrogen, and the reaction was left at room temperature for three hours. Amberlite IR120 (H^+^) ion exchange resin was added to bring the mixture to pH 7, after which the mixture was filtered and evaporated. The crude was purified using column chromatography (7 : 1 DCM/methanol) followed by preparative HPLC (gradient from 90% water with 0.1% formic acid/10% acetonitrile to 100% acetonitrile for 10 minutes) to give **4i** (7 mg, 11%) as a white solid. [*α*]20D: 28° (*c* = 0.4886) in methanol. ^1^H NMR (400 MHz, MeOD-d4): 7.92 (s, 1H), 7.88 (d, *J* = 7.5, 1H), 7.44 (s, 1H), 7.41–7.31 (m, 2H), 4.73 (d, *J* = 14.9 Hz, 1H), 4.66 (d, *J* = 14.9 Hz, 1H), 4.22 (d, *J* = 7.8 Hz, 1H, H1), 4.15 (dd, *J* = 3.0 Hz, 0.6 Hz, 1H, H4), 3.85–3.71 (m, 3H), 3.60–3.51 (m, 4H), 3.46 (dd, *J* = 9.7 Hz, 3.2 Hz, 1H, H3), 2.44 (s, 3H). ^13^C NMR (100 MHz, MeOD-d4): 168.98, 166.31, 138.19, 137.14, 131.08, 128.26, 127.48, 124.14, 104.52, 103.73, 82.61, 75.00, 70.20, 70.12, 65.39, 61.07, 55.84. HRMS: M + H^+^: 392.1820 found, 392.1822 calculated. Purity HPLC-MS, UV/VIS detection: 97.6%.

#### Methyl 3-((2-amino-6-(4-bromophenyl)-pyrimidin-4-yl)methyl)-β-d-galactopyranoside **1j**

Compound **3j** (14 mg, 0.025 mmol) was dissolved in dry tetrahydrofuran (3 mL). Guanidinium hydrochloride (4 mg, 0.038 mmol) and cesium carbonate (12 mg, 0.038 mmol) were added and the reaction mixture was refluxed overnight under nitrogen. The mixture was filtered through a short silica plug using ethyl acetate and then evaporated to give 7 mg of crude. The crude was dissolved in dry methanol (3 mL) with sodium methoxide (6 mg, 0.133 mmol) under nitrogen, and the reaction was left at room temperature for three hours. Amberlite IR120 (H^+^) ion exchange resin was added to bring the mixture to pH 7, after which the mixture was filtered and evaporated. The crude was purified using column chromatography (7 : 1 DCM/methanol) followed by preparative HPLC (gradient from 90% water with 0.1% formic acid/10% acetonitrile to 100% acetonitrile for 10 minutes) to give **1j** (1 mg, 9%).[*α*]20D: 41° (*c* = 0.0637) in methanol. ^1^H NMR (400 MHz, MeOD-d4): 8.09–8.02 (m, 2H), 7.69–7.62 (m, 2H), 7.49 (s, 1H), 4.73 (d, *J* = 15.3 Hz, 1H), 4.66 (d, *J* = 15.3 Hz, 1H), 4.21 (d, *J* = 8.1 Hz, 1H, H1), 4.14 (dd, *J* = 3.2 Hz, 0.6 Hz, 1H, H4), 3.85–3.72 (m, 3H), 3.58–3.51 (m, 4H), 3.46 (dd, *J* = 9.7 Hz, 3.2 Hz, 1H, H3). ^13^C NMR (100 MHz, MeOD-d4): 169.52, 164.74, 136.32, 131.51, 128.68, 124.70, 104.51, 103.27, 82.59, 74.99, 70.21, 70.10, 65.39, 61.06, 55.84. HRMS: M + H^+^: 456.0765 found, 456.0770 calculated. Purity HPLC-MS, UV/VIS detection: 99.0%.

#### Methyl 3-((2-amino-6-(4-(tertbutyl)phenyl)-pyrimidin-4-yl)methyl)-β-d-galactopyranoside **1k**

Compound **3k** (99 mg, 0.191 mmol) was dissolved in dry tetrahydrofuran (6 mL). Guanidinium hydrochloride (29 mg, 0.287 mmol) and cesium carbonate (102 mg, 0.287 mmol) were added and the reaction mixture was refluxed overnight under nitrogen. The mixture was filtered through a short silica plug using ethyl acetate and then evaporated to give 26 mg of crude. The crude was dissolved in dry methanol (6 mL) with sodium methoxide (11 mg, 0.209 mmol) under nitrogen, and the reaction was left at room temperature for three hours. Amberlite IR120 (H^+^) ion exchange resin was added to bring the mixture to pH 7, after which the mixture was filtered and evaporated. The crude was purified using column chromatography (7 : 1 DCM/methanol) followed by preparative HPLC (gradient from 90% water with 0.1% formic acid/10% acetonitrile to 100% acetonitrile for 10 minutes) to give **1k** (7 mg, 8%) as a slightly yellow solid. [*α*]20D: 26° (*c* = 0.3977) in methanol. ^1^H NMR (400 MHz, MeOD-d4): 8.05–7.99 (m, 2H), 7.55–7.49 (m, 2H), 7.45 (s, 1H), 4.72 (d, *J* = 15.0 Hz, 1H), 4.64 (d, *J* = 15.0 Hz, 1H), 4.20 (d, *J* = 7.9 Hz, 1H, H1), 4.13 (dd, *J* = 3.0 Hz, 0.7 Hz, 1H, H4), 3.84–3.70 (m, 3H), 3.57–3.49 (m, 4H), 3.45 (dd, *J* = 9.5 Hz, 3.0 Hz, 1H, H3), 1.36 (s, 9H). ^13^C NMR (100 MHz, MeOD-d4): 164.58, 152.56, 132.69, 125.25, 123.76, 102.99, 101.95, 81.08, 73.47, 68.68, 68.51, 63.87, 59.54, 54.32, 32.78, 28.68. HRMS: M + H^+^: 434.2294 found, 434.2291 calculated. Purity HPLC-MS, UV/VIS detection: 97.1%.

#### Methyl 3-((2-amino-6-(4-(phenyl)phenyl)-pyrimidin-4-yl)methyl)-β-d-galactopyranoside **1l**

Compound **3l** (61 mg, 0.113 mmol) was dissolved in dry tetrahydrofuran (8 mL). Guanidinium hydrochloride (16 mg, 0.170 mmol) and cesium carbonate (55 mg, 0.170 mmol) were added and the reaction mixture was refluxed overnight under nitrogen. The mixture was filtered through a short silica plug using ethyl acetate and then evaporated to give 22 mg of crude. The crude was dissolved in dry methanol (3 mL) with sodium methoxide (9 mg, 0.171 mmol) under nitrogen, and the reaction was left at room temperature for three hours. Amberlite IR120 (H^+^) ion exchange resin was added to bring the mixture to pH 7, after which the mixture was filtered and evaporated. The crude was purified using column chromatography (7 : 1 DCM/methanol) followed by preparative HPLC (gradient from 90% water with 0.1% formic acid/10% acetonitrile to 100% acetonitrile for 10 minutes) to give **1l** (5 mg, 10%).[*α*]20D: 28° (*c* = 0.2893) in methanol. ^1^H NMR (400 MHz, MeOD-d4): 8.22–8.17 (m, 2H), 7.78–7.73 (m, 2H), 7.72–7.67 (m, 2H), 7.52 (s, 1H), 7.51–7.45 (m, 2H), 7.39 (tt, *J* = 7.3 Hz, 2.0 Hz, 1H), 4.74 (d, *J* = 14.9 Hz, 1H), 4.67 (d, *J* = 14.9 Hz, 1H), 4.22 (d, *J* = 7.9 Hz, 1H, H1), 4.16 (dd, *J* = 3.2 Hz, 0.8 Hz, 1H, H4), 3.86–3.73 (m, 3H), 3.60–3.52 (m, 4H), 3.48 (dd, *J* = 9.6 Hz, 3.0 Hz, 1H, H3). ^13^C NMR (100 MHz, MeOD-d4): 169.07, 165.63, 163.30, 143.40, 140.12, 135.96, 128.59, 127.49, 126.82, 126.62, 104.52, 103.53, 82.63, 75.00, 70.22, 70.10, 65.40, 61.08, 55.85. HRMS: M + H^+^: 454.1982 found, 454.1978 calculated. Purity HPLC-MS, UV/VIS detection: 95.6%.

#### Methyl 3-((2-amino-6-(3-(phenyl)phenyl)-pyrimidin-4-yl)methyl)-β-d-galactopyranoside **1m**

Compound **3m** (17 mg, 0.032 mmol) was dissolved in dry tetrahydrofuran (3 mL). Guanidinium hydrochloride (5 mg, 0.047 mmol) and cesium carbonate (15 mg, 0.047 mmol) were added and the reaction mixture was refluxed overnight under nitrogen. The mixture was filtered through a short silica plug using ethyl acetate and then evaporated to give 8 mg of crude. The crude was dissolved in dry methanol (3 mL) with sodium methoxide (3 mg, 0.062 mmol) under nitrogen, and the reaction was left at room temperature for three hours. Amberlite IR120 (H^+^) ion exchange resin was added to bring the mixture to pH 7, after which the mixture was filtered and evaporated. The crude was purified using column chromatography (7 : 1 DCM/methanol) followed by preparative HPLC (gradient from 90% water with 0.1% formic acid/10% acetonitrile to 100% acetonitrile for 10 minutes) to give **1m** (2 mg, 14%).[*α*]20D: 44° (*c* = 0.0476) in methanol. ^1^H NMR (400 MHz, MeOD-d4): 8.37 (t, *J* = 1.7 Hz, 1H), 8.08 (dt, *J* = 7.9 Hz, 1.3 Hz, 1H), 7.78 (ddd, *J* = 7.8 Hz, 2.0 Hz, 1.2 Hz, 1H), 7.75–7.70 (m, 2H), 7.62–7.54 (m, 2H), 7.53–7.46 (m, 2H), 7.39 (tt, *J* = 5.6 Hz, 1.0 Hz, 1H), 4.75 (d, *J* = 15.4 Hz, 1H, partially obscured by water), 4.69 (d, *J* = 15.4 Hz, 1H), 4.22 (d, *J* = 7.8 Hz, 1H, H1), 4.16 (d, *J* = 3.1 Hz, 1H, H4), 3.85–3.72 (m, 3H), 3.59–3.52 (m, 4H), 3.48 (dd, *J* = 9.6 Hz, 3.1 Hz, 1H, H3). ^13^C NMR (100 MHz, MeOD-d4): 128.93, 128.55, 127.26, 126.74, 125.87, 125.51, 104.53, 103.79, 82.56, 75.00, 70.19, 70.12, 65.38, 61.08, 55.86. HRMS: M + H^+^: 454.1988 found, 454.1978 calculated. Purity HPLC-MS, UV/VIS detection: 96.1%.

#### Methyl 3-((2-amino-6-(2-fluorophenyl)-pyrimidin-4-yl)methyl)-β-d-galactopyranoside **1n**

Compound **3n** (23 mg, 0.048 mmol) was dissolved in dry tetrahydrofuran (3 mL). Guanidinium hydrochloride (7 mg, 0.072 mmol) and cesium carbonate (23 mg, 0.072 mmol) were added and the reaction mixture was refluxed overnight under nitrogen. The mixture was filtered through a short silica plug using ethyl acetate and then evaporated to give 18 mg of crude. The crude was dissolved in dry methanol (3 mL) with sodium methoxide (8 mg, 0.156 mmol) under nitrogen, and the reaction was left at room temperature for three hours. Amberlite IR120 (H^+^) ion exchange resin was added to bring the mixture to pH 7, after which the mixture was filtered and evaporated. The crude was purified using column chromatography (7 : 1 DCM/methanol) followed by preparative HPLC (gradient from 90% water with 0.1% formic acid/10% acetonitrile to 100% acetonitrile for 10 minutes) to give **1n** (2 mg, 10%) as a white solid. [*α*]20D: 42° (*c* = 0.0601) in methanol. ^1^H NMR (400 MHz, MeOD-d4): 7.97 (td, *J* = 7.8 Hz, 1.8 Hz, 1H), 7.52 (m, 1H), 7.34–7.29 (m, 2H), 7.24 (ddd, *J* = 11.0 Hz, 8.3 Hz, 1.0 Hz, 1H), 4.75 (d, *J* = 15.7 Hz, 1H, partially obscured by water), 4.68 (d, *J* = 15.7 Hz, 1H), 4.20 (d, *J* = 7.4 Hz, 1H, H1), 4.13 (dd, *J* = 3.0 Hz, 0.6 Hz, 1H, H4), 3.85–3.70 (m, 3H), 3.59–3.51 (m, 4H), 3.46 (dd, *J* = 9.6 Hz, 3.3 Hz, 1H, H3). ^13^C NMR (100 MHz, MeOD-d4): 131.82, 130.46, 124.24, 104.50, 82.71, 75.00, 70.31, 70.21, 65.52, 61.07, 55.82. HRMS: M + H^+^: 396.1567 found, 396.1571 calculated. Purity HPLC-MS, UV/VIS detection: 96.4%.

#### 3,4-Dichlorophenyl 3-*O*-propargyl-1-thio-α-d-galactopyranoside **5**

Methanol (75 mL) and sodium methoxide (12.5 mL, 1 M) were added to the tetraacetate **4** (ref. 17) (2.40 g, 4.81 mmol), stirred overnight at rt, neutralized with Dowex 50W-X8 (H^+^), filtered, and concentrated. The residue and dibutyltin oxide (1.446 g, 5.810 mmol) were dissolved in dry methanol (30 mL) and refluxed for 5 h to turn into a clear solution. The solvent was evaporated and the remaining sample was dissolved in 1,4-dioxane (50 mL) together with propargyl bromide (80 wt% in toluene, 1.1 mL, 9.68 mmol) and tetrabutylammonium iodide (1.79 g, 4.84 mmol). The reaction mixture was refluxed overnight, concentrated, and purified by flash column chromatography twice (heptane : EtOAc = 4 : 1 and 3 : 1) to give **5** (0.97 g, 52%) as a yellow syrup. ^1^H NMR (400 MHz in MeOD-d4): *δ* 7.73 (d, *J* = 2.0 Hz, 1H, Ph), 7.48 (dd, *J* = 8.4, 2.0 Hz, 1H, Ph), 7.43 (d, *J* = 8.5 Hz, 1H, Ph), 5.65 (d, *J* = 5.6 Hz, 1H, H-1), 4.39 (dd, *J* = 6.7, 2.4 Hz, 2H, CH_2_), 4.32–4.24 (m, 2H, H-2 and H-5), 4.19 (d, *J* = 3.0 Hz, H-4), 3.76–3.64 (m, 3H, H-3 and H-6), 2.89 (t, *J* = 2.4 Hz, 1H, CH). ^13^C NMR (100 MHz in MeOD-d4): *δ* 136.9, 134.4, 132.7, 132.1, 131.7, 91.2, 80.8, 79.1, 76.1, 73.5, 68.9, 67.7, 62.3, 57.7. [*α*]20D = 100° (*c* = 3.1064 in acetonitrile). HRMS: M + NH_4_^+^: 346.0439 found, 346.0439 calculated.

#### 3,4-Dichlorophenyl 3-*O*-[(2-amino-4-(4-chlorobenzoyl)-pyrimidin-6-yl)methyl]-1-thio-α-d-galactopyranoside **7**

To a solution of **5** (200 mg, 0.4 mmol) in THF (15 mL) were added 4-chlorobenzoyl chloride (69 mg, 0.4 mmol), copper(i) iodide (15 mg, 0.08 mmol), and PdCl_2_(PPh_3_)_2_Cl_2_ (29 mg, 0.04 mmol). The mixture was purged three times with N_2_, stirred at 10 °C for 20 min, and then Et_3_N (40 mg, 0.4 mmol) was added. After 2 h at 10 °C, the reaction was quenched with water (20 mL) and extracted with dichloromethane (3 × 50 mL). The extract was washed with brine (20 mL), dried over Na_2_SO_4_, filtered, and concentrated. The crude product was purified using Combiflash (EtOAc : PE = 1 : 20 to 1 : 5, ISCO, 40 g, 40 mL min^–1^, normal phase, silica, UV 254 nm) to yield **6** (160 mg, 63%) as a yellow oil. ESI-MS *m*/*z* calcd for [C_28_H_29_Cl_3_NO_9_S] + [M + NH_4_]^+^: 660.1; found: 660.0. To a portion of **6** (140 mg, 0.22 mmol) in tetrahydrofuran (10 mL) were added guanidine hydrochloride (52 mg, 0.54 mmol) and K_2_CO_3_ (90 mg, 0.65 mmol); the reaction vessel was purged 3 times with nitrogen, the mixture was heated to reflux with stirring for 20 h, and finally concentrated. The residue was suspended in MeOH (5 mL), filtered, and the filtrate was purified by prep-HPLC to give **7** (22 mg, 18%) as a white solid. [*α*]20D = 2° (*c* = 0.33212 in MeOD). ^1^H NMR (400 MHz in MeOD-d4) δ 8.13 (d, *J* = 8.6 Hz, 2H), 7.76 (d, *J* = 2.0 Hz, 1H), 7.56–7.42 (m, 5H), 5.74 (d, *J* = 5.6 Hz, 1H), 4.65–4.75 (dd, 2H), 4.42–4.48 (m, 1H), 4.33–4.22 (m, 2H), 3.76 (dd, *J* = 15.0, 6.1 Hz, 2H), 3.65 (dd, *J* = 10.1, 3.0 Hz, 1H). ^13^C NMR (100 MHz in MeOD-d4): 169.3, 164.7, 163.4, 136.4, 135.9, 135.5, 133.0, 132.1, 131.3, 130.7, 130.2, 128.49, 128.47, 103.3, 89.6, 79.9, 72.1, 70.0, 67.5, 65.9, 61.0. HRMS: M + H^+^: 558.0422 found, 558.0424 calculated. Purity HPLC-MS, UV/VIS detection: 98.5%.

### Fluorescence polarization experiments

Human galectin-1,^21^ galectin-3,^22^ galectin-4N,^23^ galectin-4C,^23^ galectin-7,^24^ galectin-8N,^25^ galectin-8C,^25^ galectin-9N,^26^ and galectin-9C^26^ were expressed and purified as described earlier. Fluorescence polarization experiments were performed on a PheraStarFS plate reader with the software PHERAstar Mars version 2.10 R3 (BMG, Offenburg, Germany). Specific experimental conditions were a galectin-1 concentration of 0.5 μM together with the fluorescent probe 3,3′-dideoxy-3-[4-(fluorescein-5-yl-carbonylaminomethyl)-1*H*-1,2,3-triazol-1-yl]-3′-(3,5-di-methoxybenzamido)-1,1′-sulfanediyl-di-β-d-galactopyranoside^21^ concentration of 20 nM and a galectin-3 concentration of 0.2 μM together with the fluorescent probe (3,3′-dideoxy-3-[4-(fluorescein-5-yl-carbonylaminomethyl)-1*H*-1,2,3-triazol-1-yl]-3′-(3,5-di-methoxybenzamido)-1,1′-sulfanediyl-di-β-d-galactopyranoside) concentration of 20 nM. Inhibitors were dissolved in dimethyl sulfoxide (analytical grade) to a concentration of 20 mM, diluted with PBS to 3–6 different concentrations, and tested in duplicate twice for galectin affinity using a competitive fluorescence polarization assay as described earlier.^20^ The highest inhibitor concentration tested was 900 μM due to solubility limitations at higher concentration. Dissociation constant average and SEM were calculated from four to six single point measurements showing between 20% and 80% inhibition. In all experiments, the anisotropy of the fluorescent ligand in the presence of the protein and in the absence of the protein were measured as controls to confirm that these anisotropy values were reproducible and there were no experimental errors due to erroneous protein/probe concentrations or instrument failures.

### Molecular dynamics simulations

Molecular dynamics simulations were performed with the OPLS3 force field in Desmond (Schrödinger Release 2018-4: Desmond Molecular Dynamics System, D. E. Shaw Research, New York, NY, 2017; Maestro-Desmond Interoperability Tools, Schrödinger, New York, NY, 2017) using default settings except for the length of the simulation and the use of light harmonic constraints (1 kcal mol^–1^ Å^–1^) on all stranded backbone atoms and on the galactose O4 atom. Starting conformations of **7** in complex with galectin-3 were built by manually placing **7** with its galactose ring in an identical position to that of the galactose ring of lactose in the binding site of galectin-3 (pdb id ; 1KJL). The 3,4-dichlorophenyl aglycon of **7** was oriented with the 3-chloro atom towards the Gly182 carbonyl oxygen as seen for a 3,4-dichlorophenyl 3-triazolyl-α-thiogalactoside in complex with galectin-3 (pdb id ; 6EOL). The 4-(4-chlorophenyl)-2-aminopyrimidine group was placed in different conformations. The complexes were then subjected to 200 ns molecular dynamics simulations. Molecular images were generated using PyMOL v1.7 (Schrodinger LLC).

## Conflicts of interest

HL and UJN are shareholders in and FZ is an employee of Galecto Biotech AB, Sweden, a company developing galectin inhibitors.

## Supplementary Material

Supplementary information
