# Supplementary material for: Aminopyrimidine–galactose hybrids are highly selective galectin-3 inhibitors
Source: Medchemcomm. 2019 May 13;10(6):913–25. doi: 10.1039/c9md00183b (PMC6596385; doi:10.1039/c9md00183b)

Electronic Supplementary Material (ESI) for MedChemComm.
This journal is © The Royal Society of Chemistry 2019

Supplementary Information

Aminopyrimidine-galactose hybrids are highly selective galectin-3 inhibitors

Alexander Dahlqvist[1], Fredrik R. Zetterberg[2], Hakon Leffler[3], Ulf J. Nilsson[1*]

[1]Centre for Analysis and Synthesis, Department of Chemistry, Lund University, Box 124, 221 00 Lund (Sweden) E-mail: ulf.nilsson@chem.lu.se

[2]Galecto Biotech AB, Sahlgrenska Science Park, Medicinaregatan 8A, 413 46 Gothenburg (Sweden) Email: fz@galecto.com

[3]Department of Laboratory Medicine, Section MIG, Lund University BMC-C1228b, Klinikgatan 28, 221 84 Lund (Sweden)

^{1}H- and ^{13}C-NMR spectra of compounds 1a-n, 3a-n, 5, and 7

1a:

^{1}H-NMR:

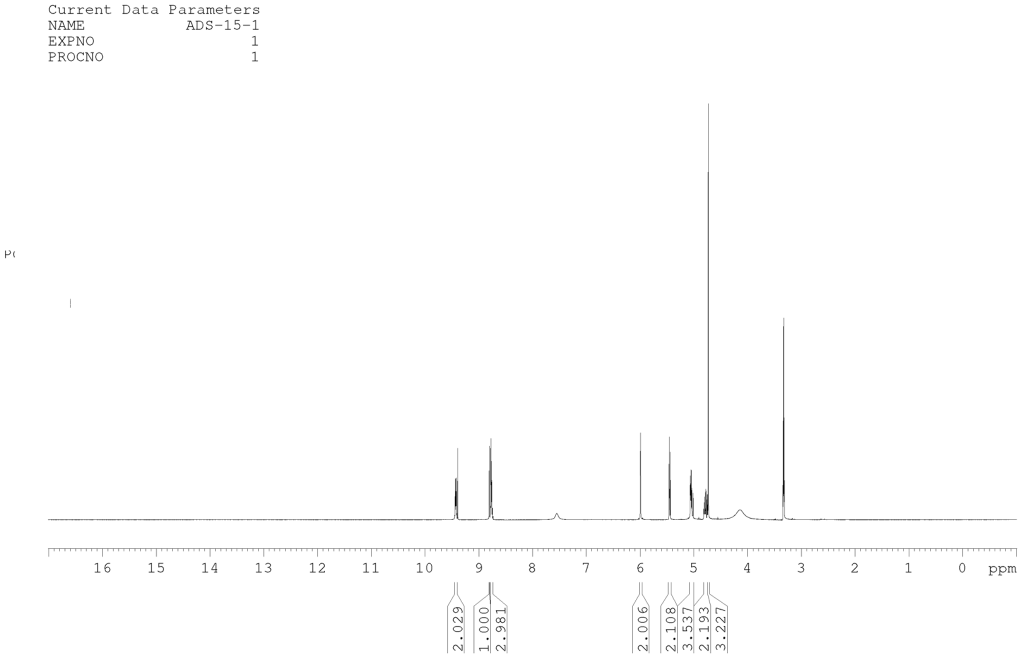

^{13}C-NMR:

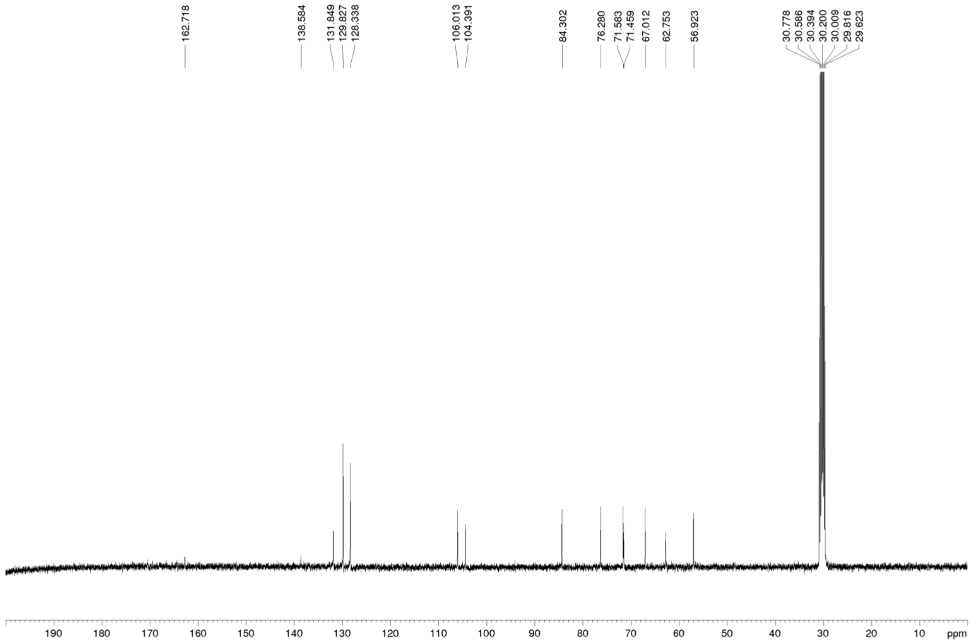

1b:

^{1}H-NMR:

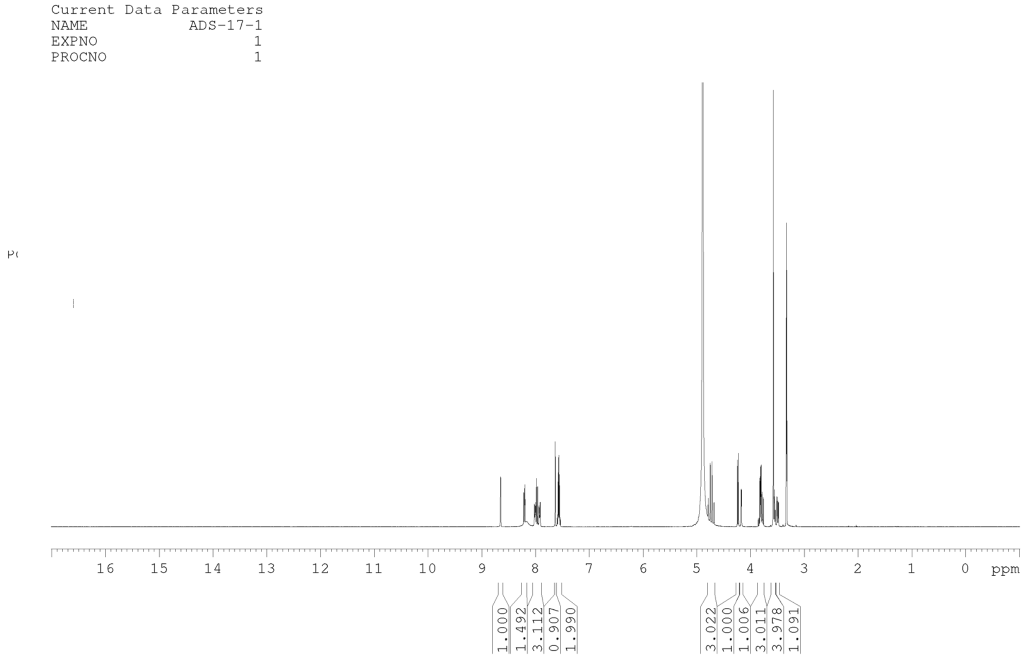

^{13}C-NMR:

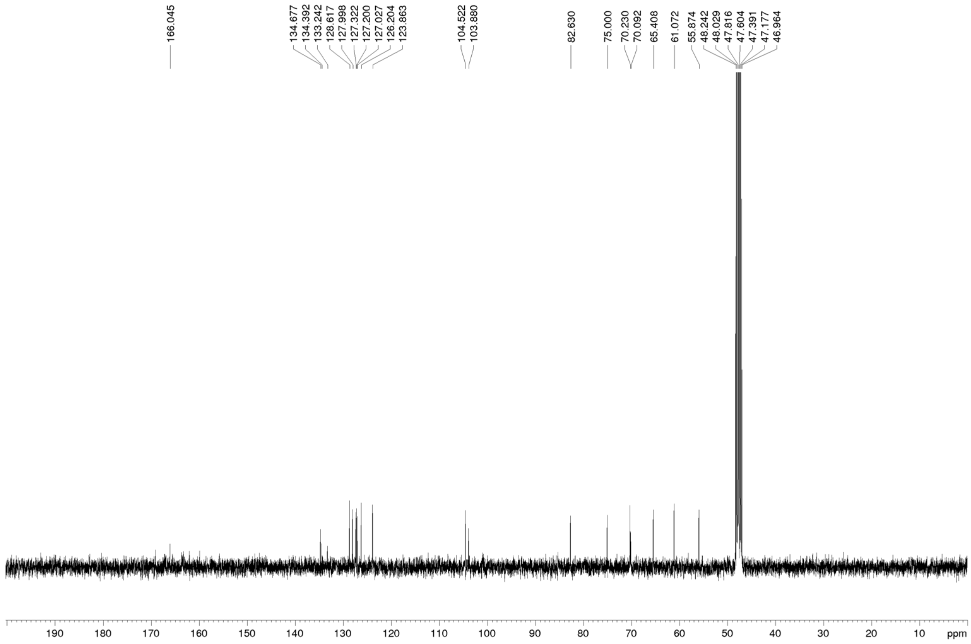

1c:

¹H-NMR:

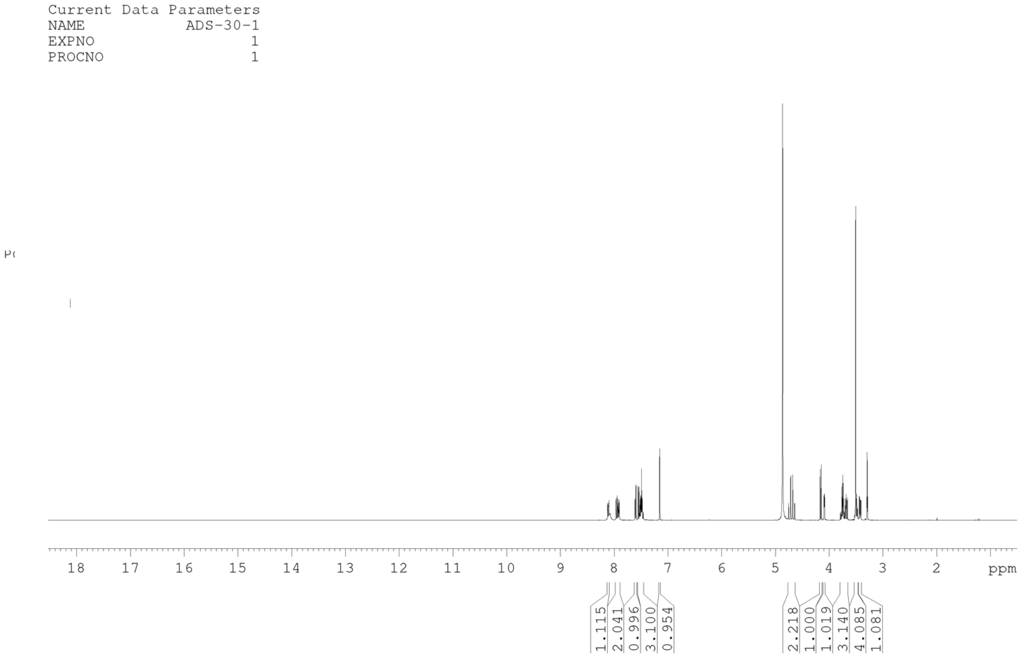

¹³C-NMR:

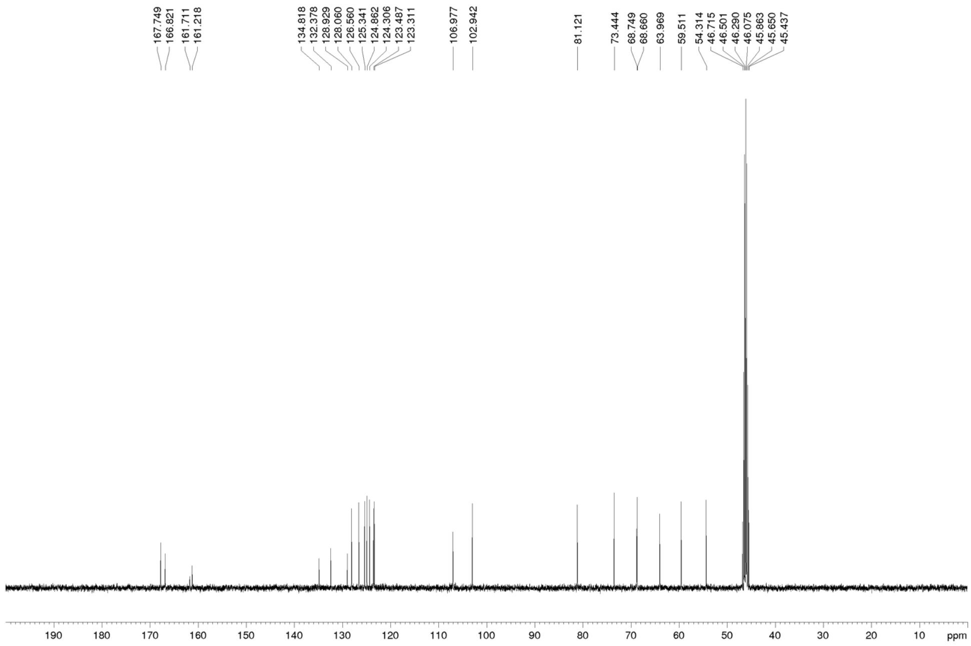

1d:

^{1}H-NMR:

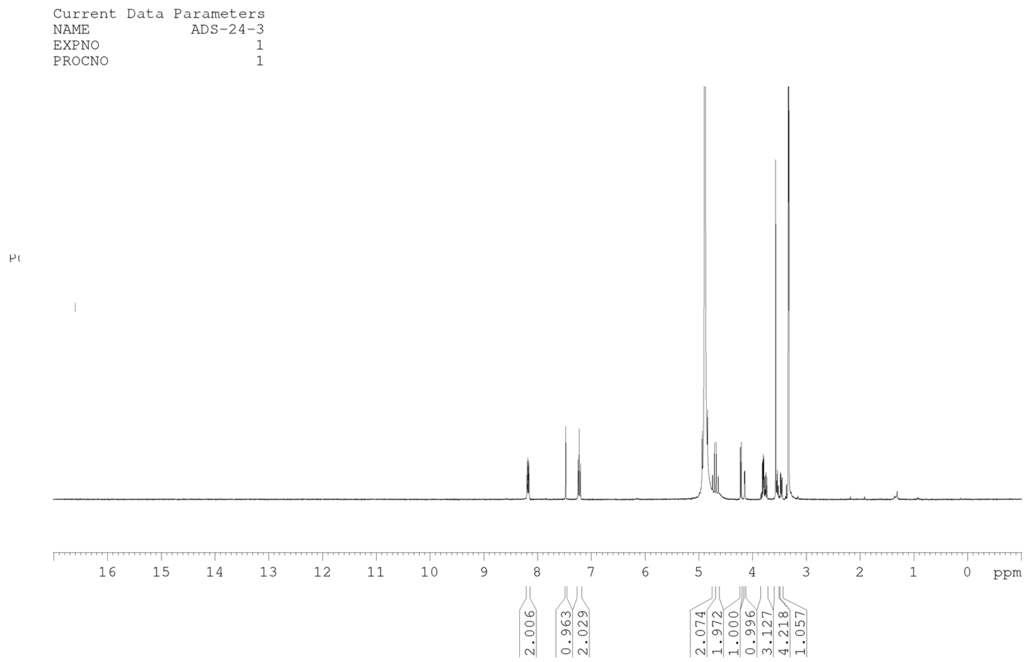

^{13}C-NMR:

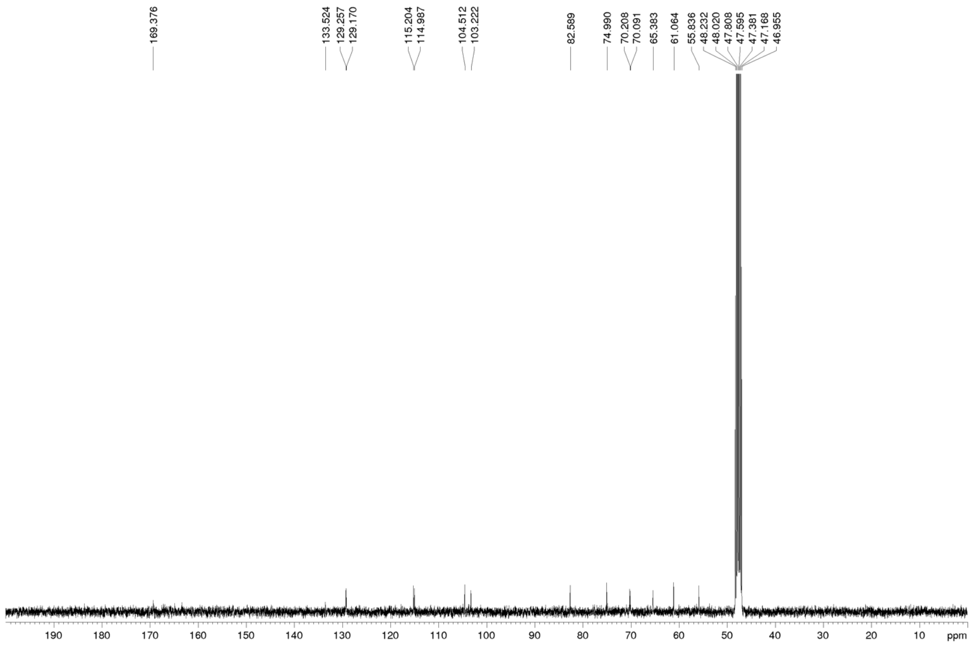

1e:

^{1}H-NMR:

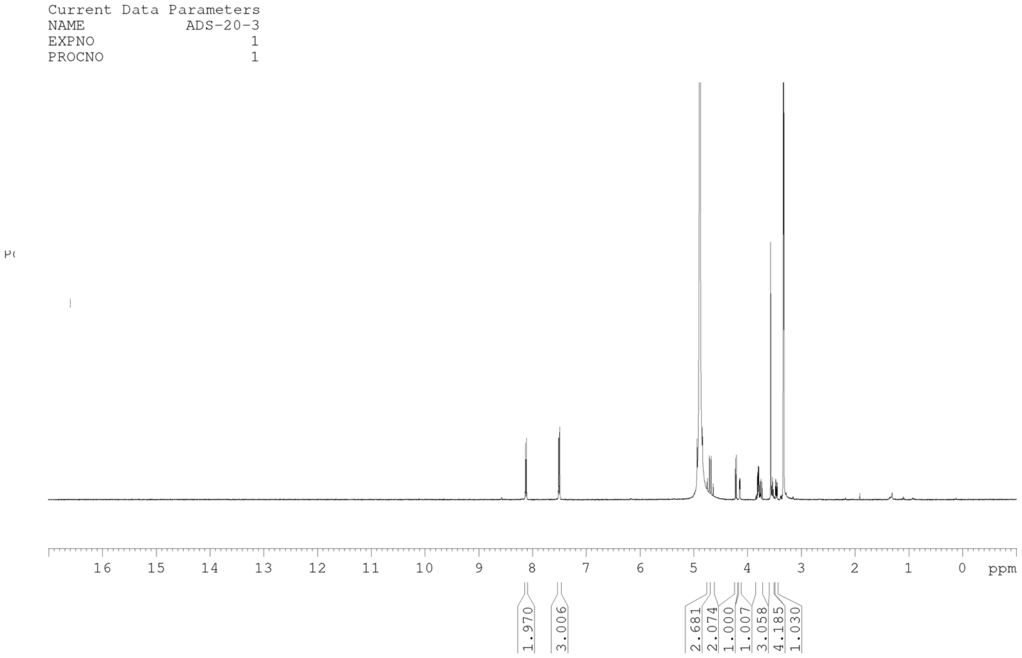

^{13}C-NMR:

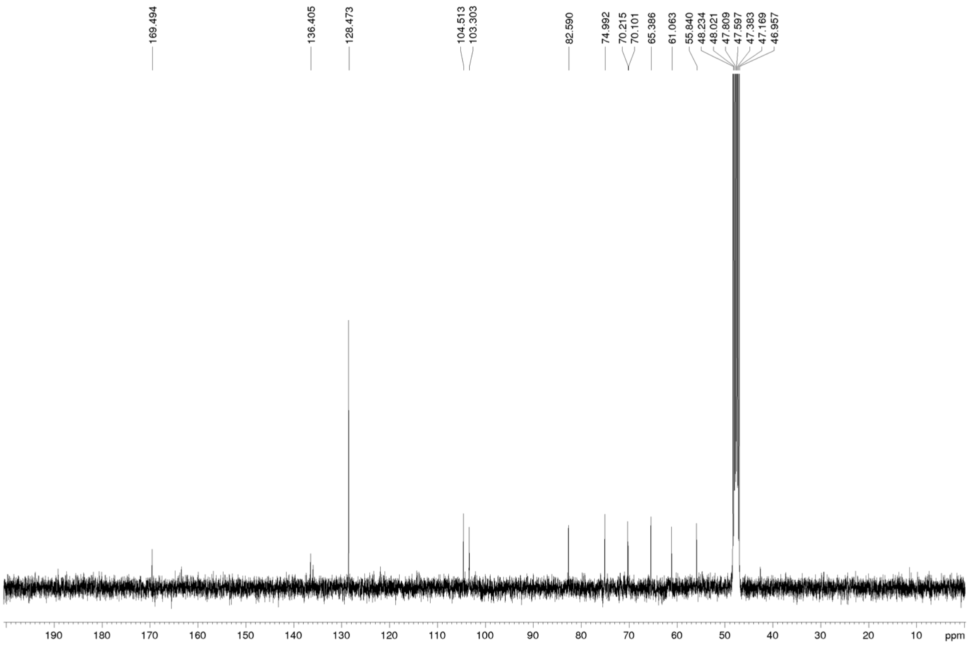

1f:

^{1}H-NMR:

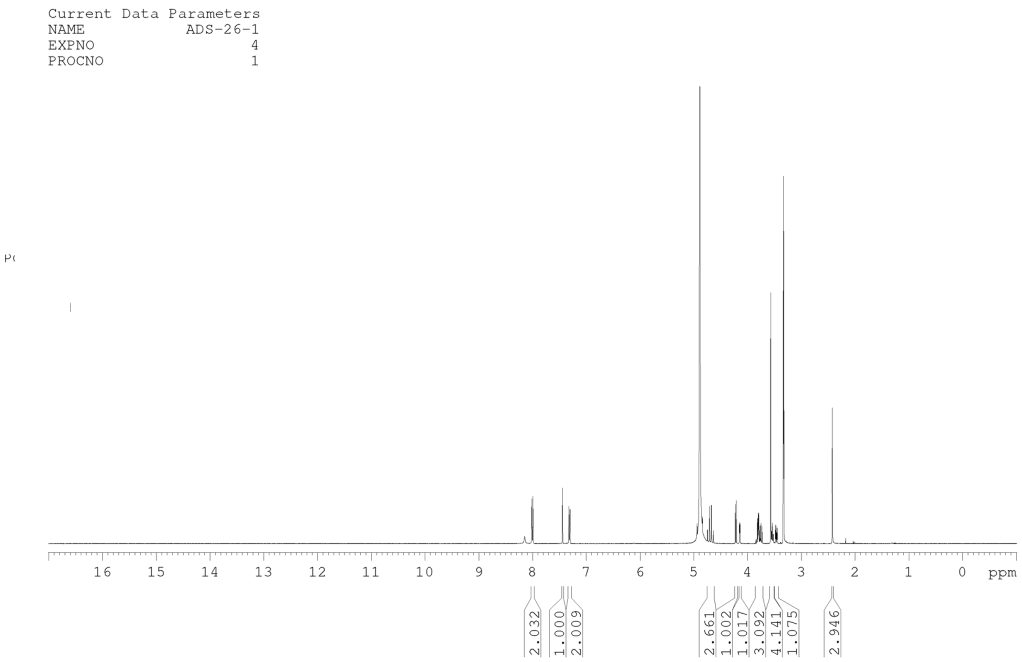

^{13}C-NMR:

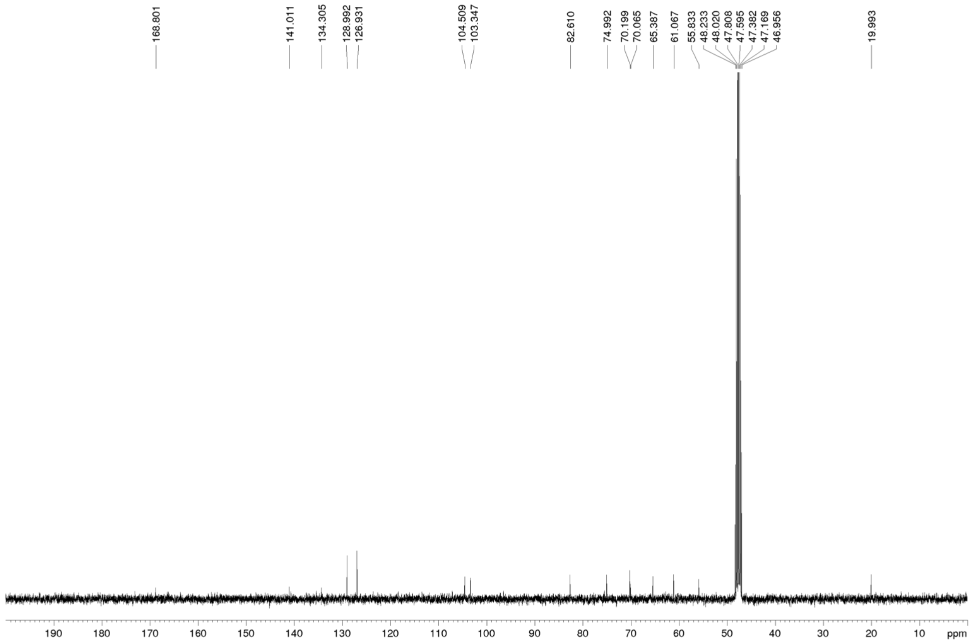

1g:

^{1}H-NMR:

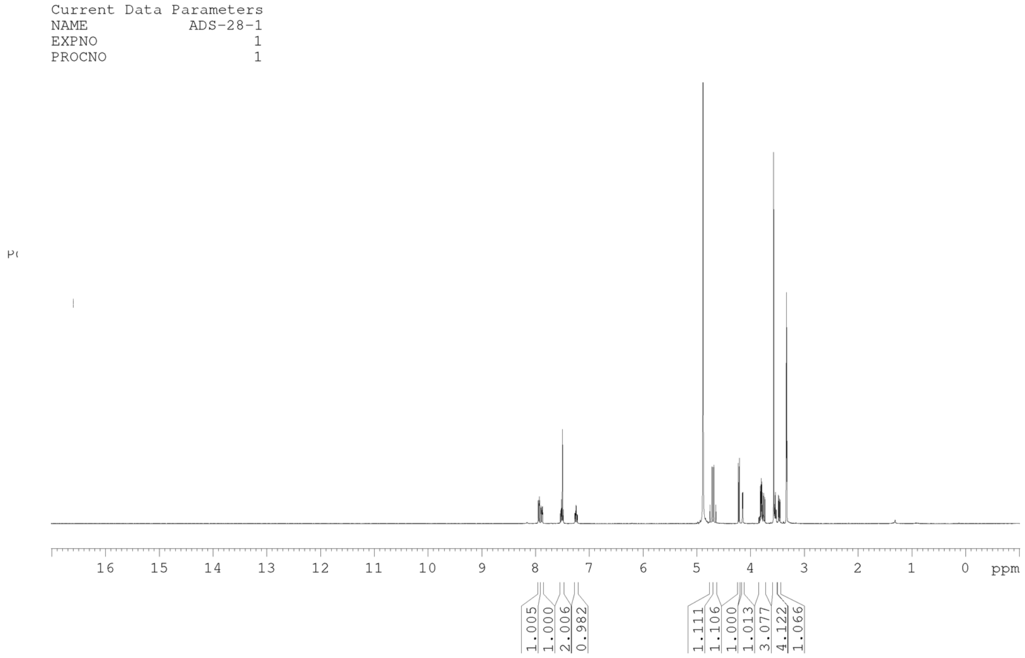

^{13}C-NMR:

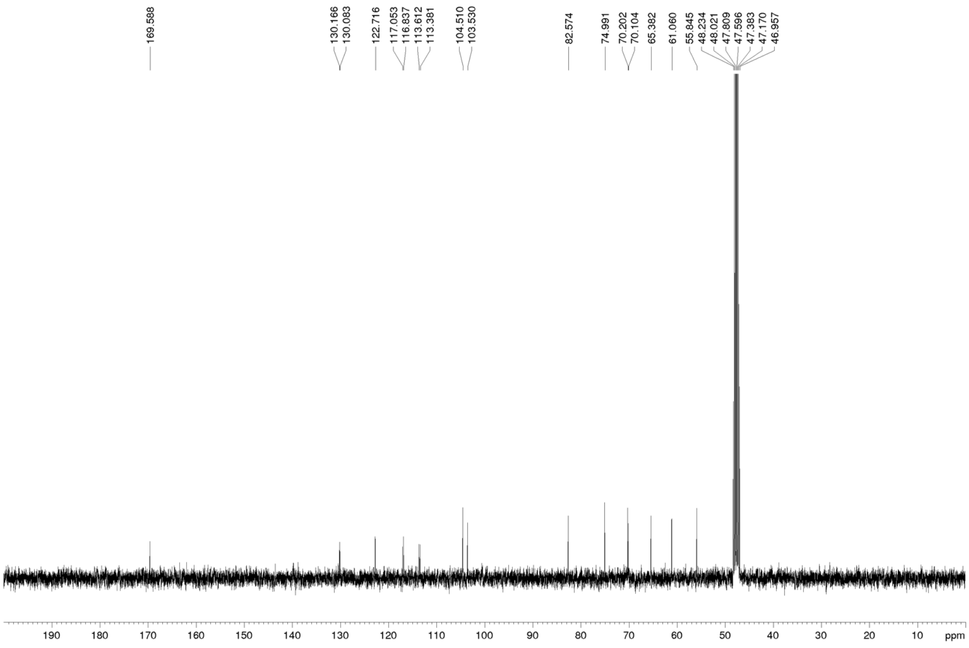

^{1}H-NMR:

^{1}H-NMR:

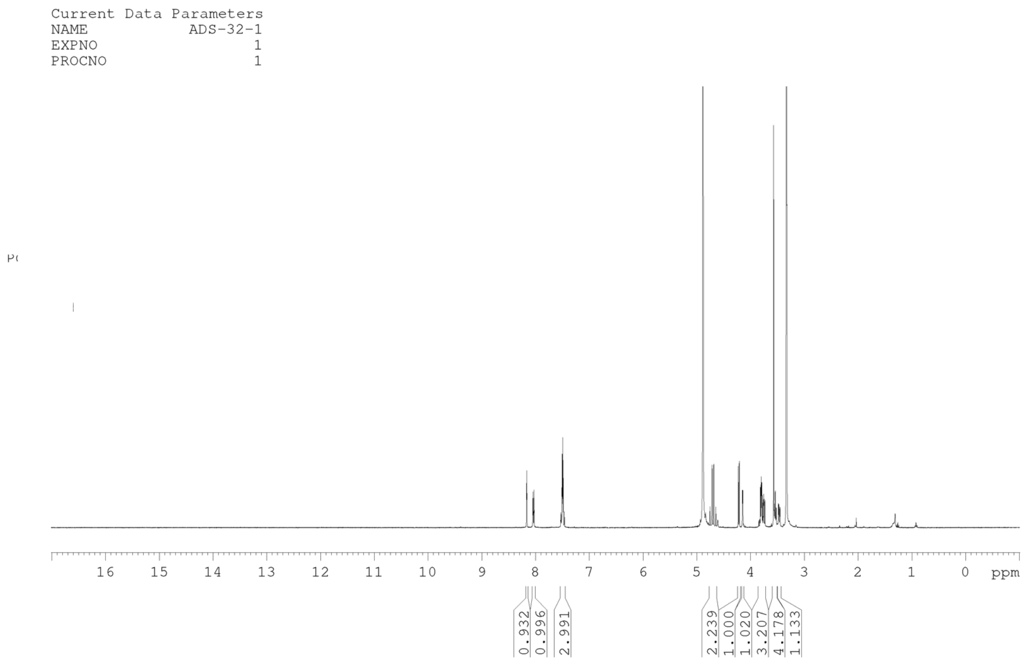

^{13}C-NMR:

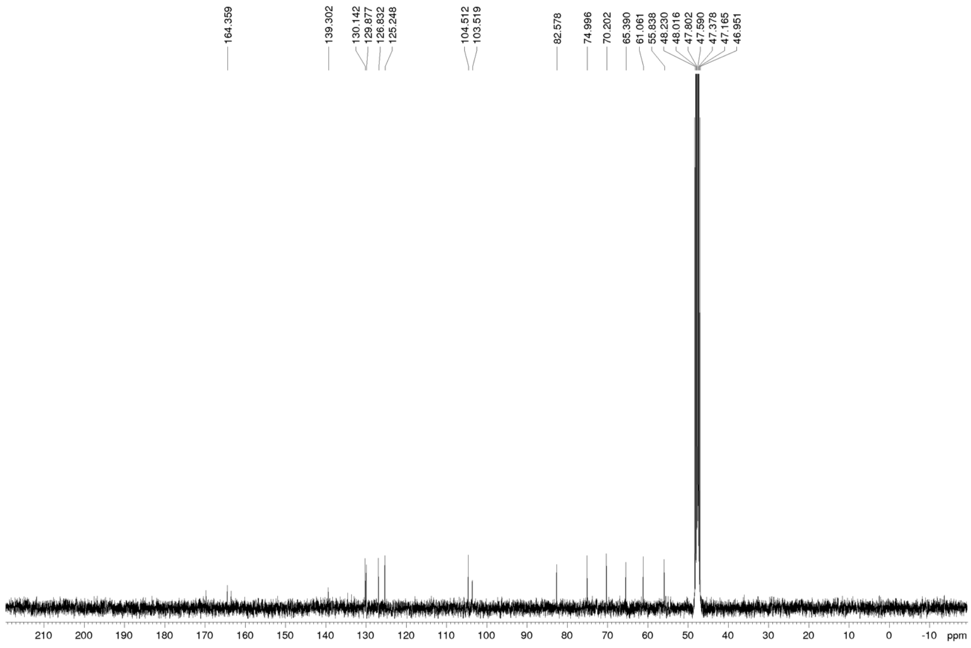

1i:

^{1}H-NMR:

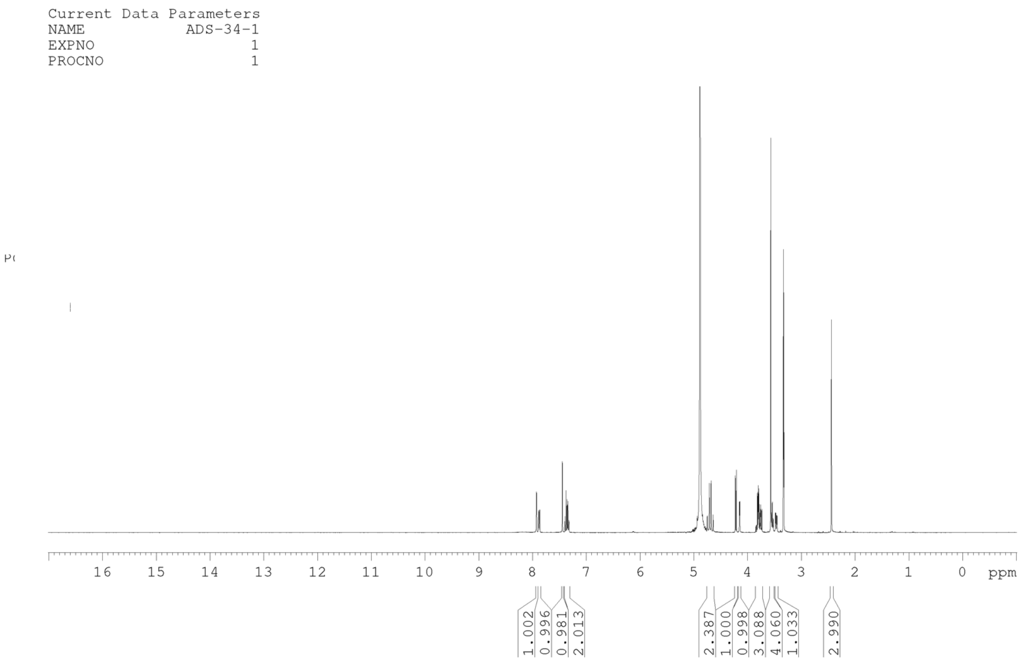

^{13}C-NMR:

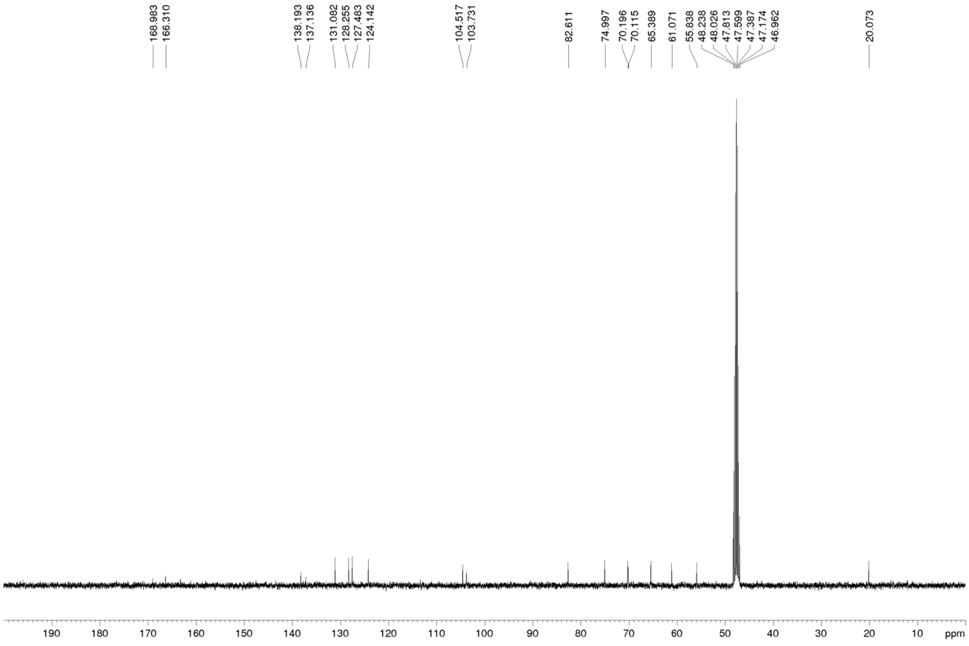

1j:

^{1}H-NMR:

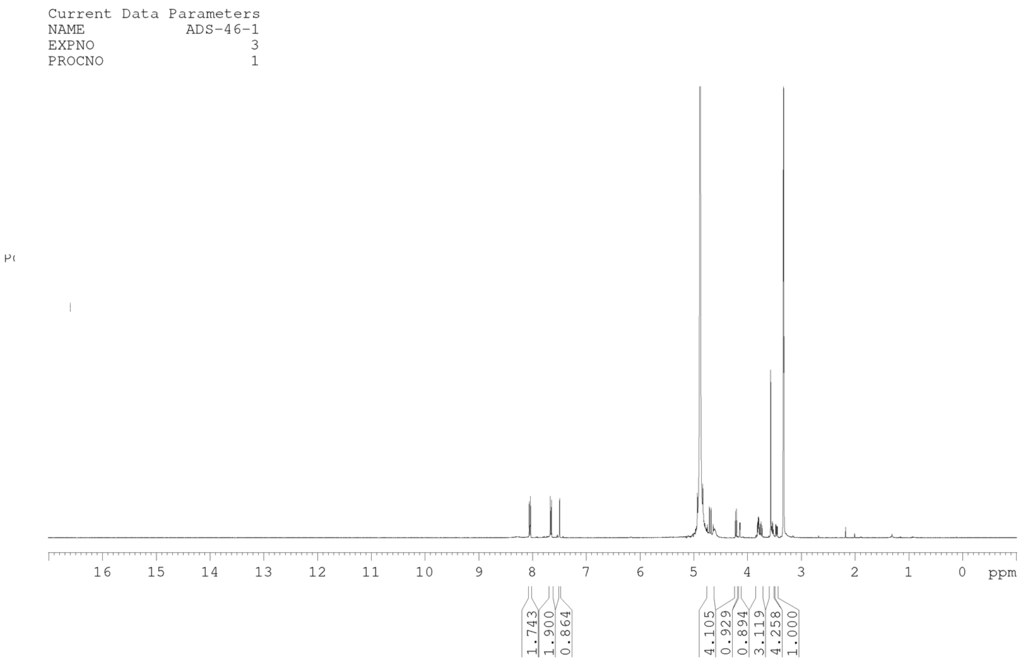

^{13}C-NMR:

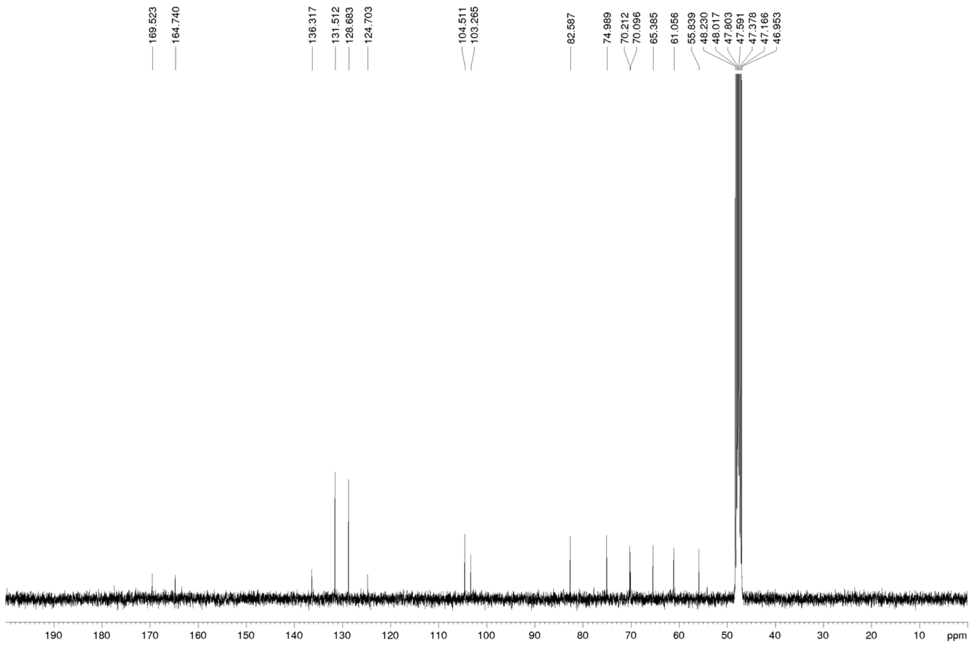

1k:

^{1}H-NMR:

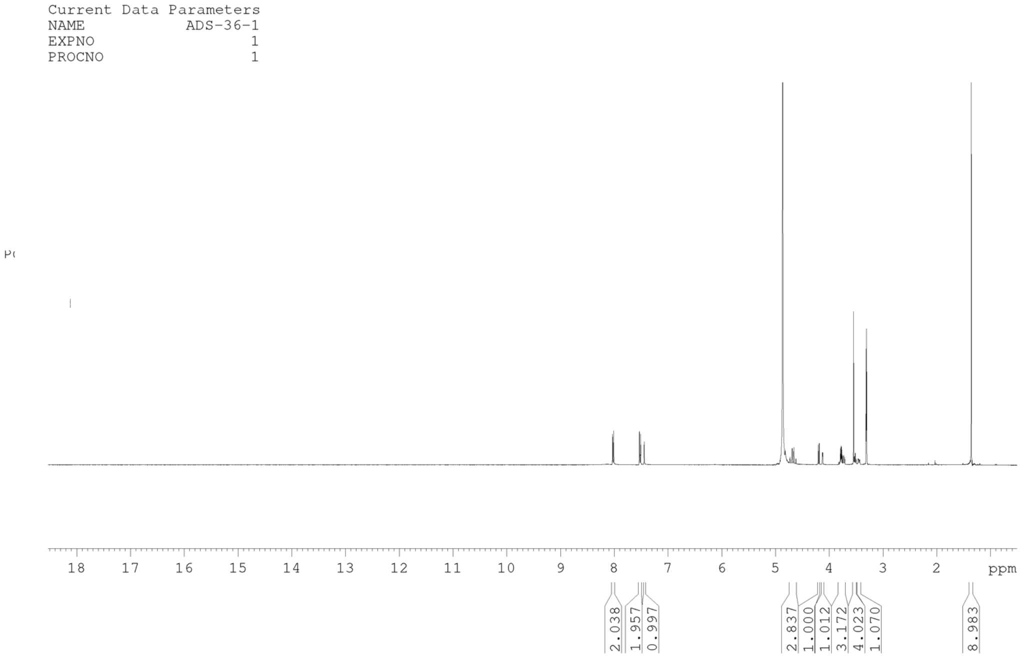

^{13}C-NMR:

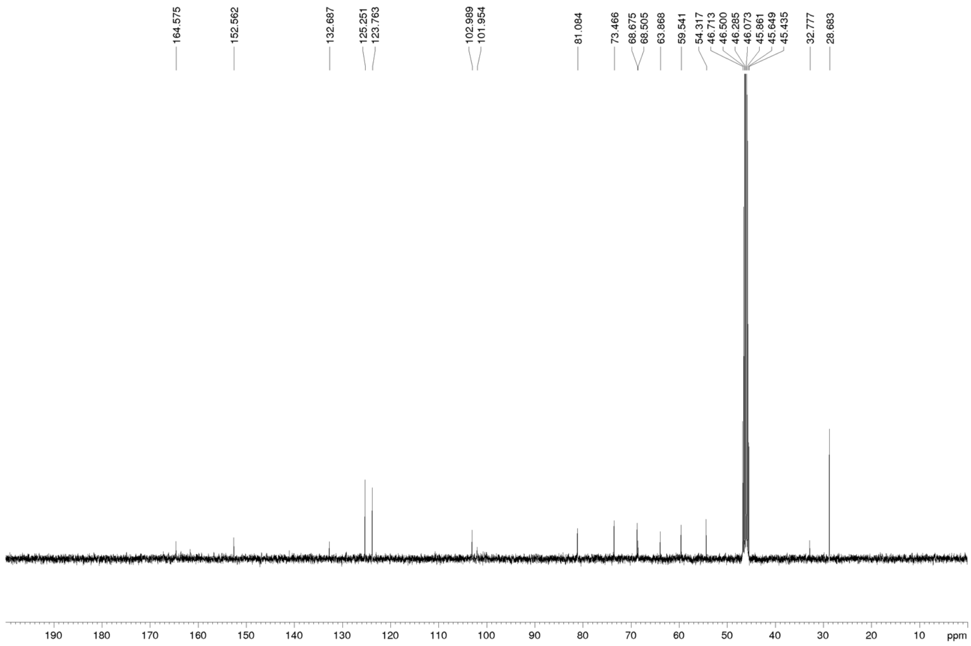

1l:

^{1}H-NMR:

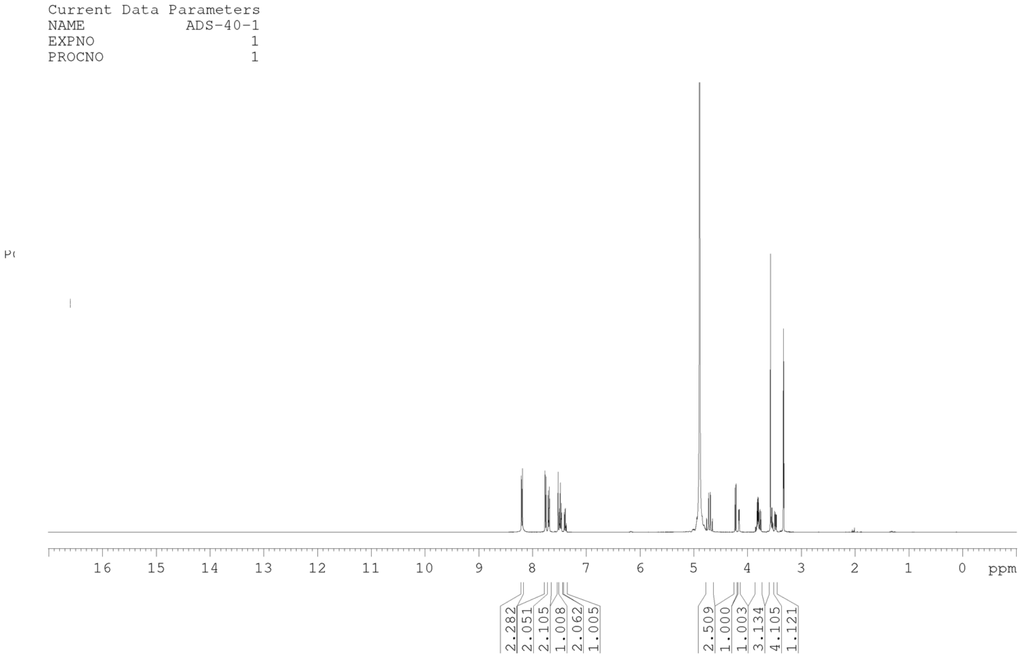

^{13}C-NMR:

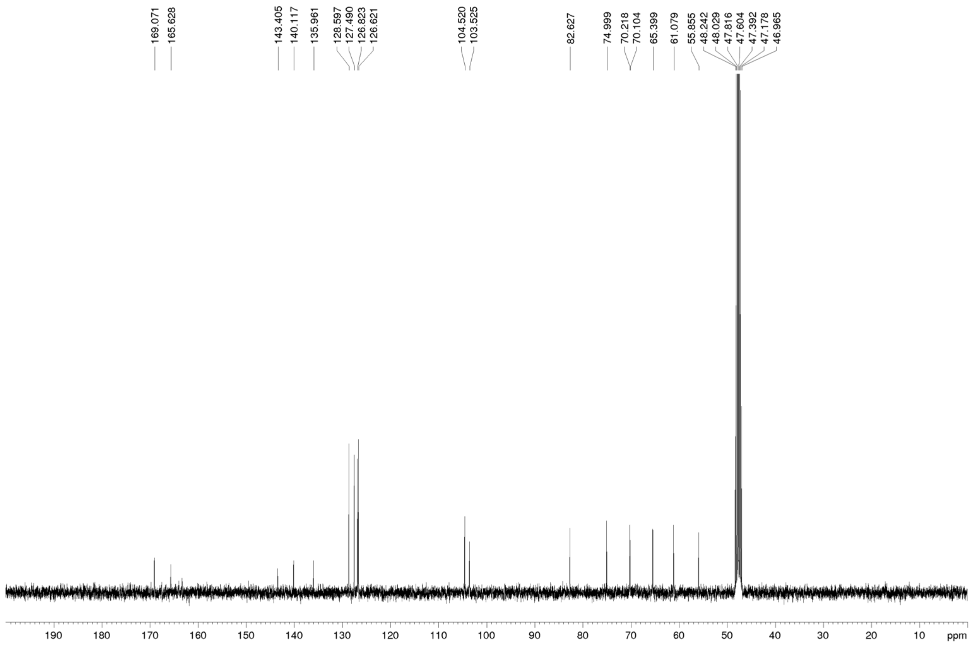

1m:

^{1}H-NMR:

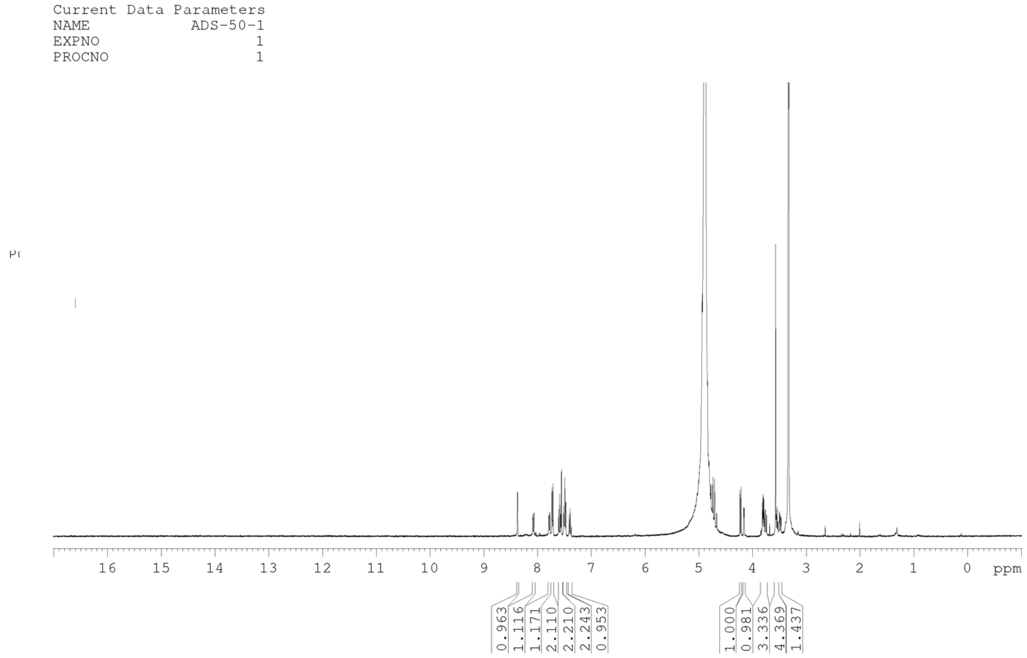

^{13}C-NMR:

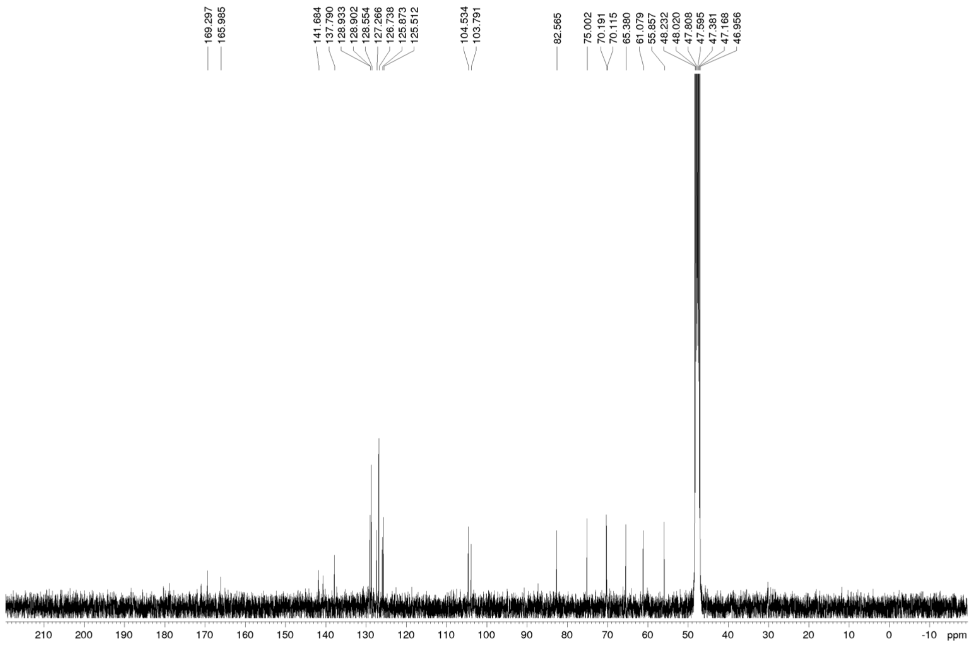

1n:

^{1}H-NMR:

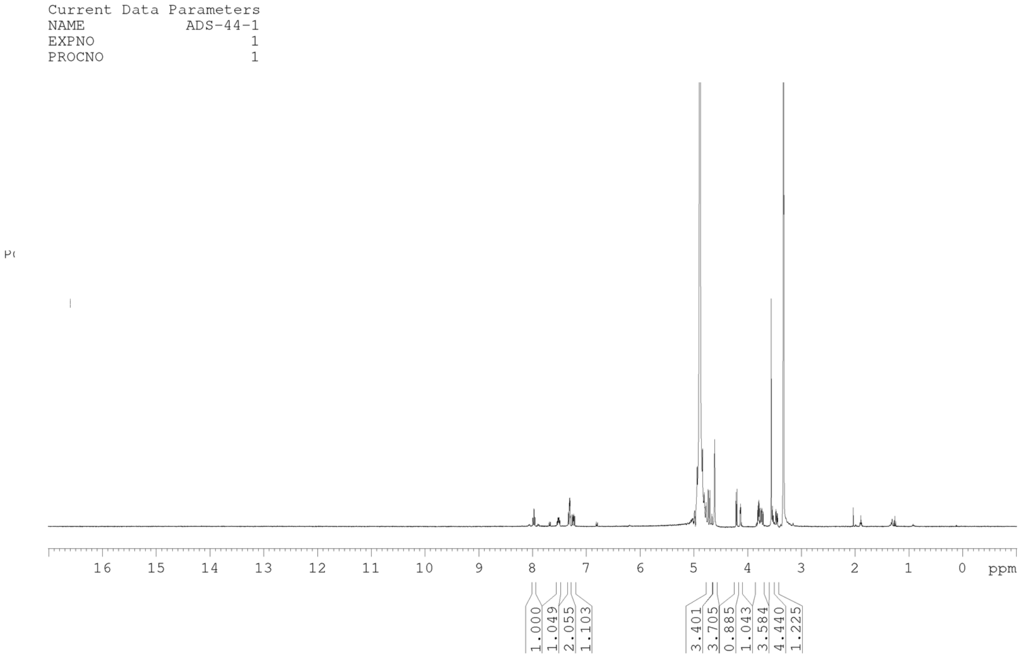

^{13}C-NMR:

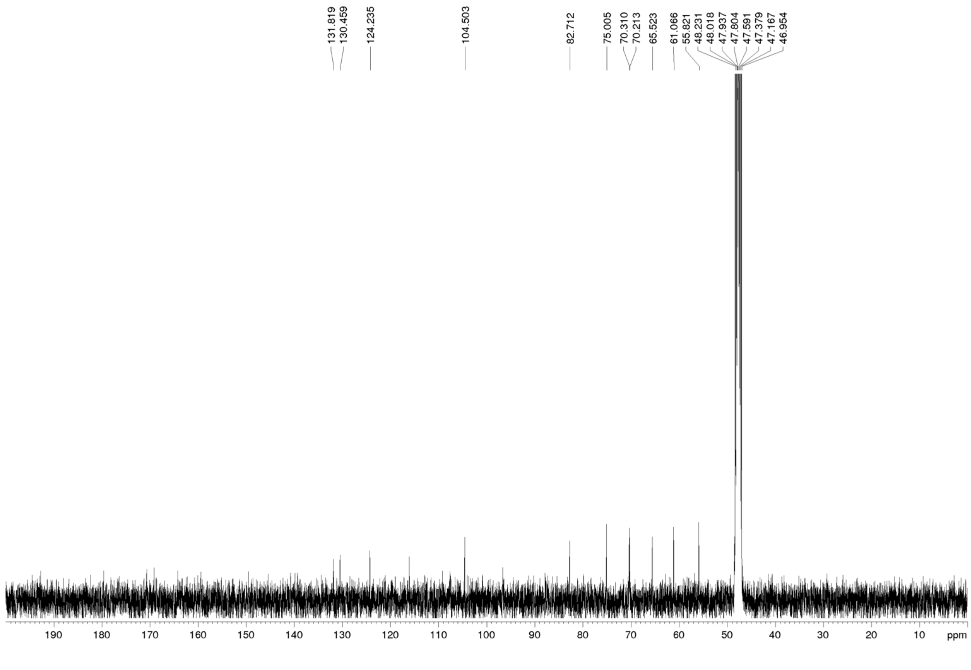

3a:

^{1}H-NMR:

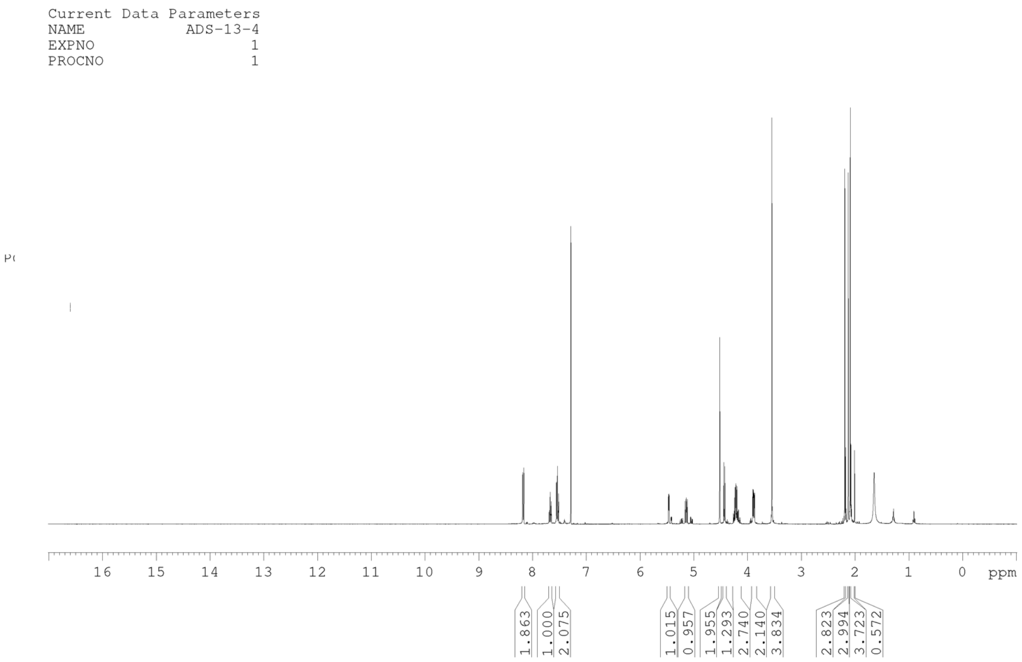

^{13}C-NMR:

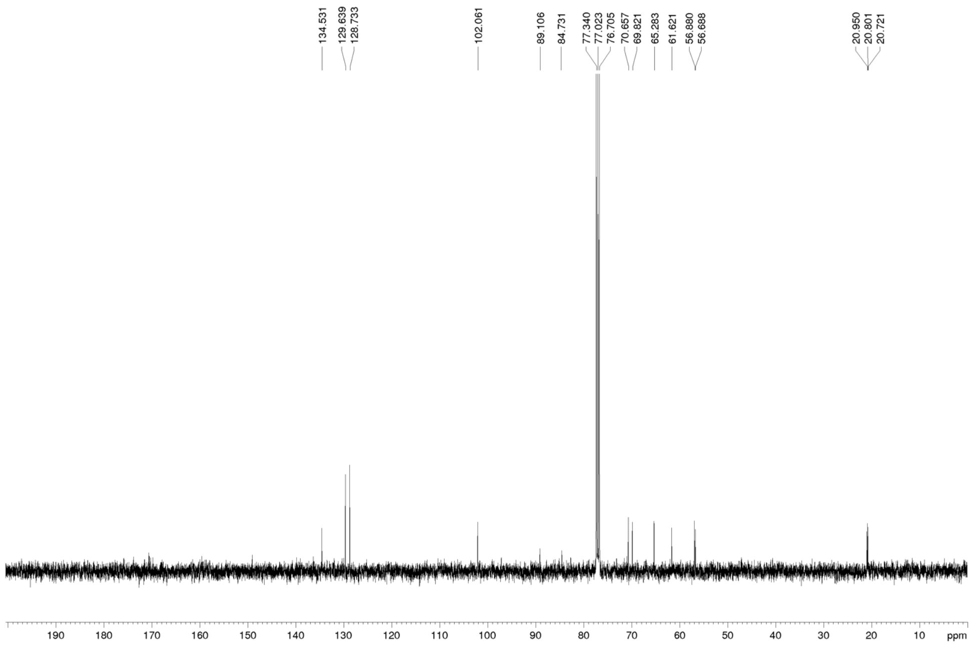

3b:

^{1}H-NMR:

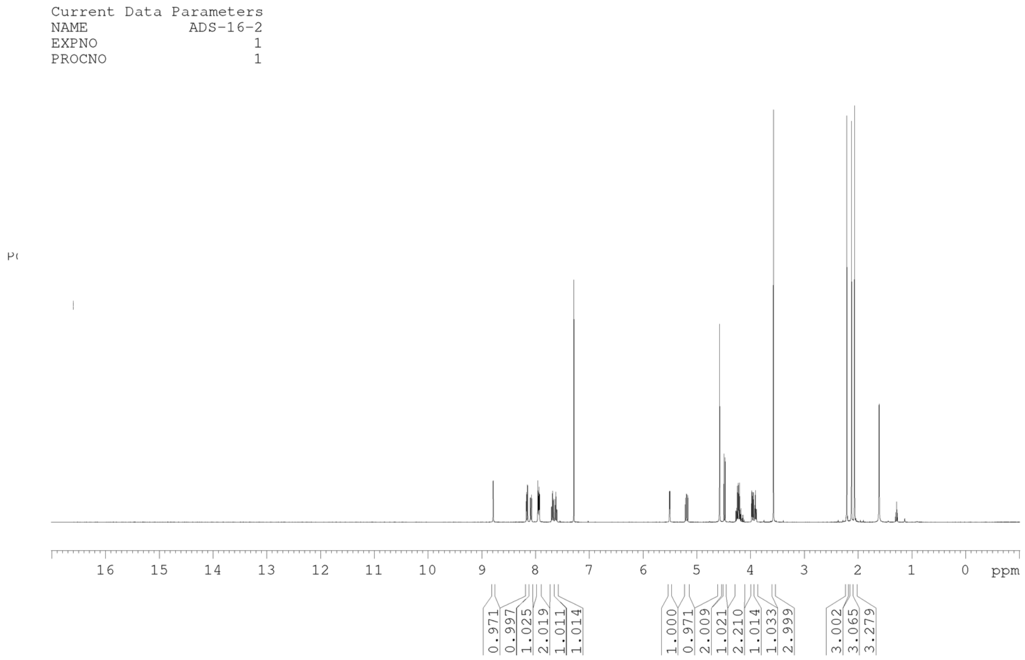

^{13}C-NMR:

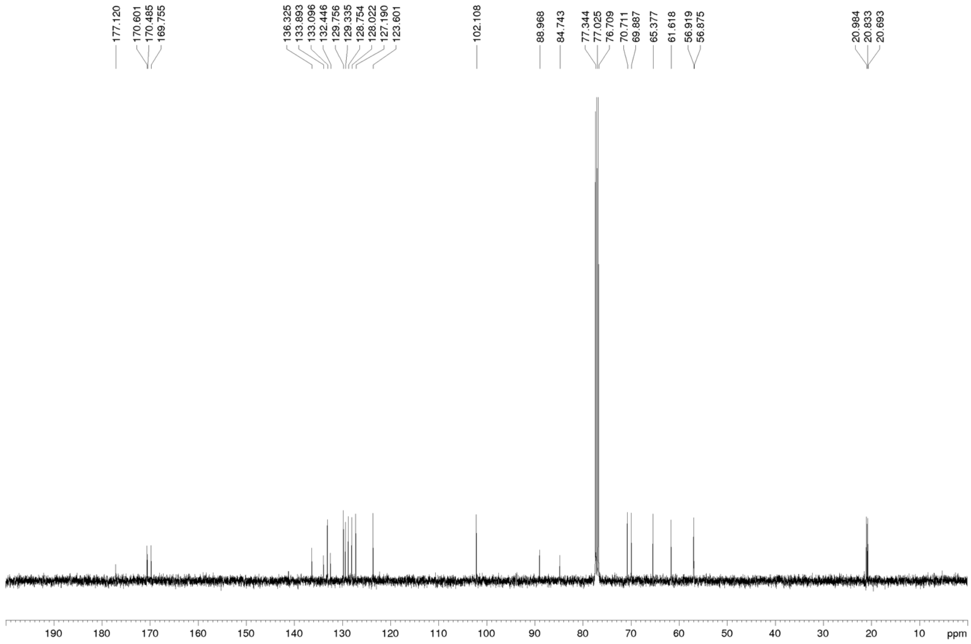

3c:

^{1}H-NMR:

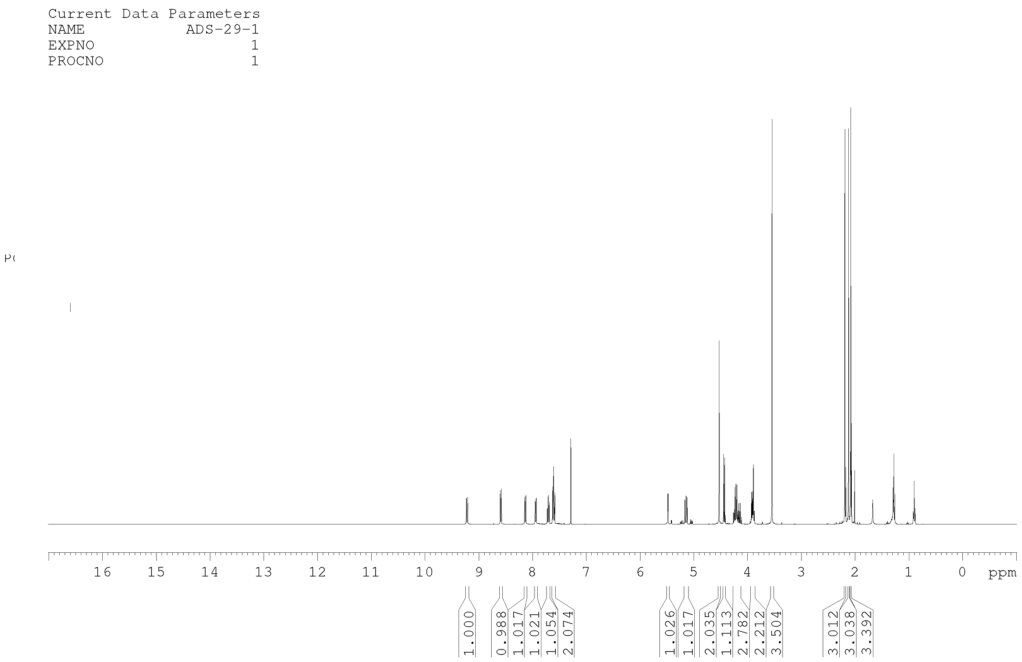

^{13}C-NMR:

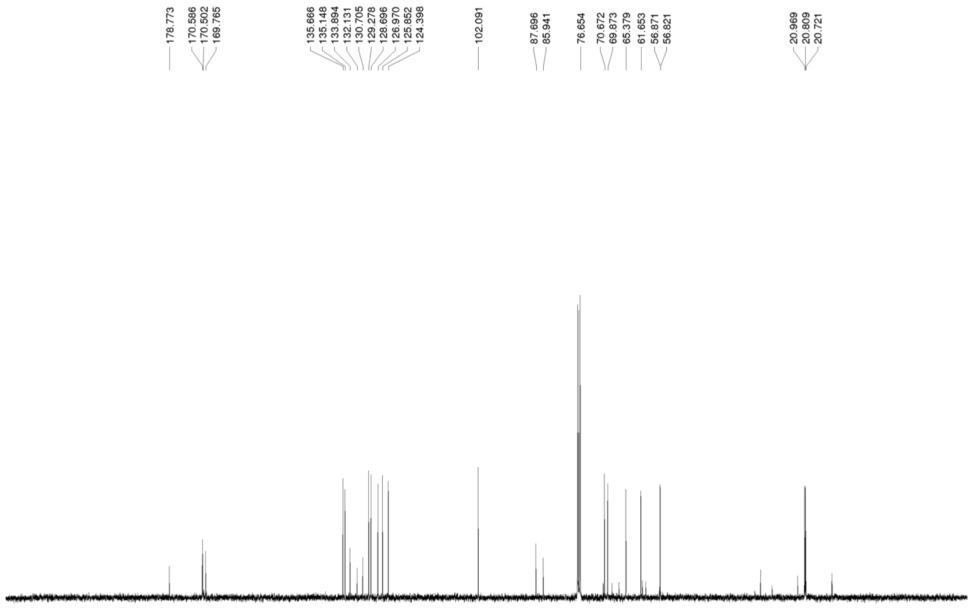

3d:

^{1}H-NMR:

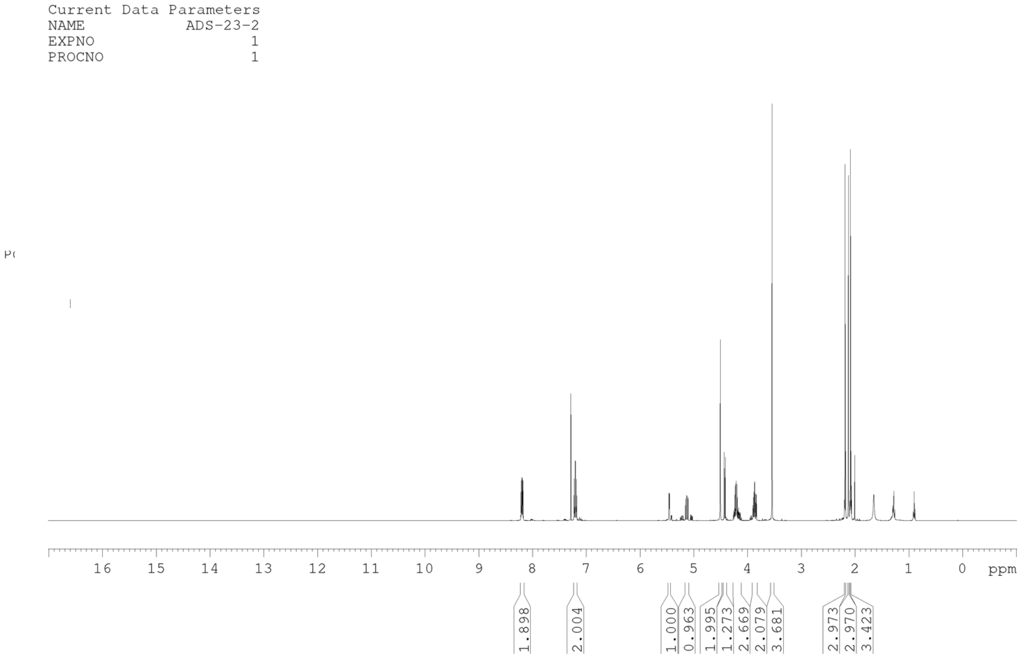

^{13}C-NMR:

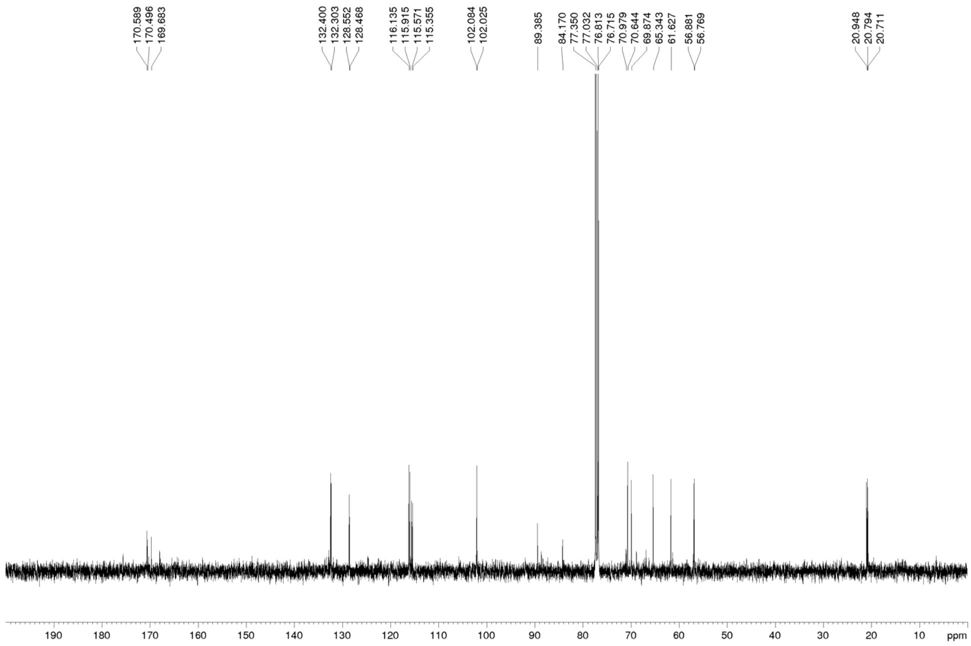

3e:

^{1}H-NMR:

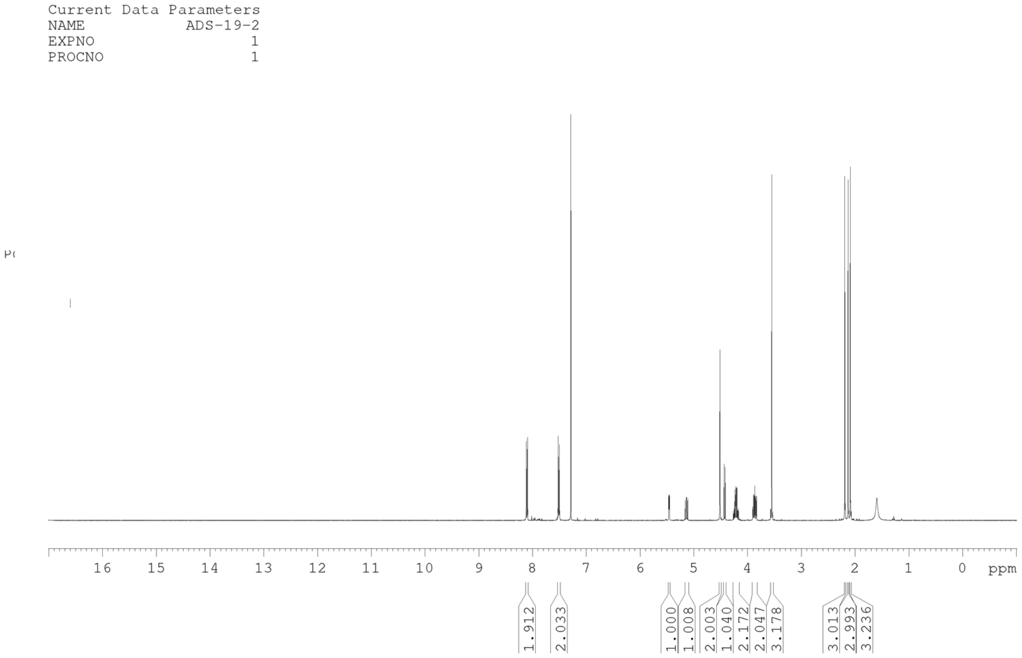

^{13}C-NMR:

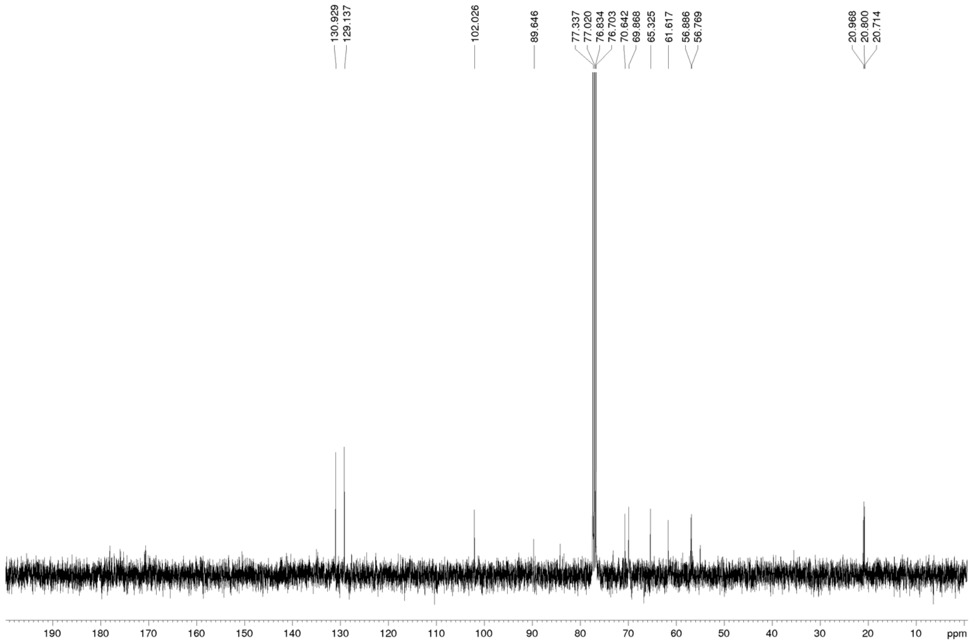

3f:

^{1}H-NMR:

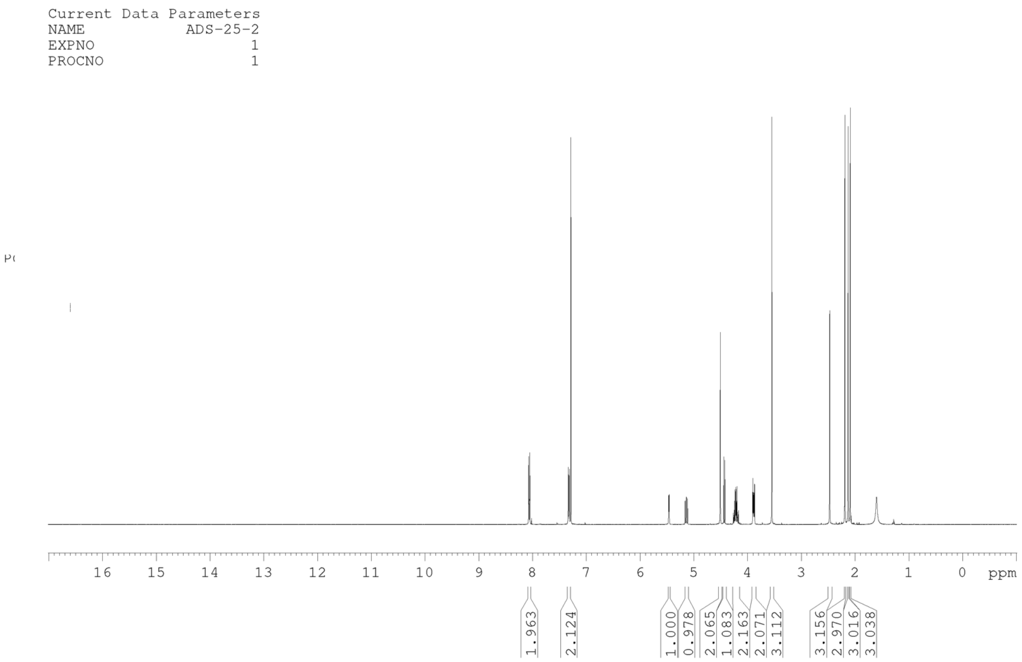

^{13}C-NMR:

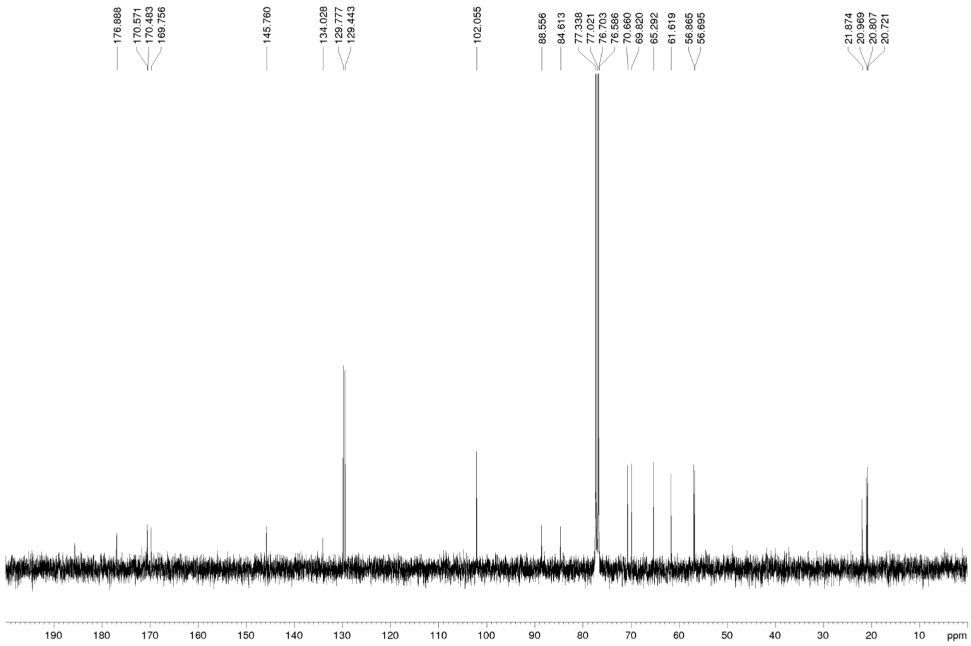

3g:

^{1}H-NMR:

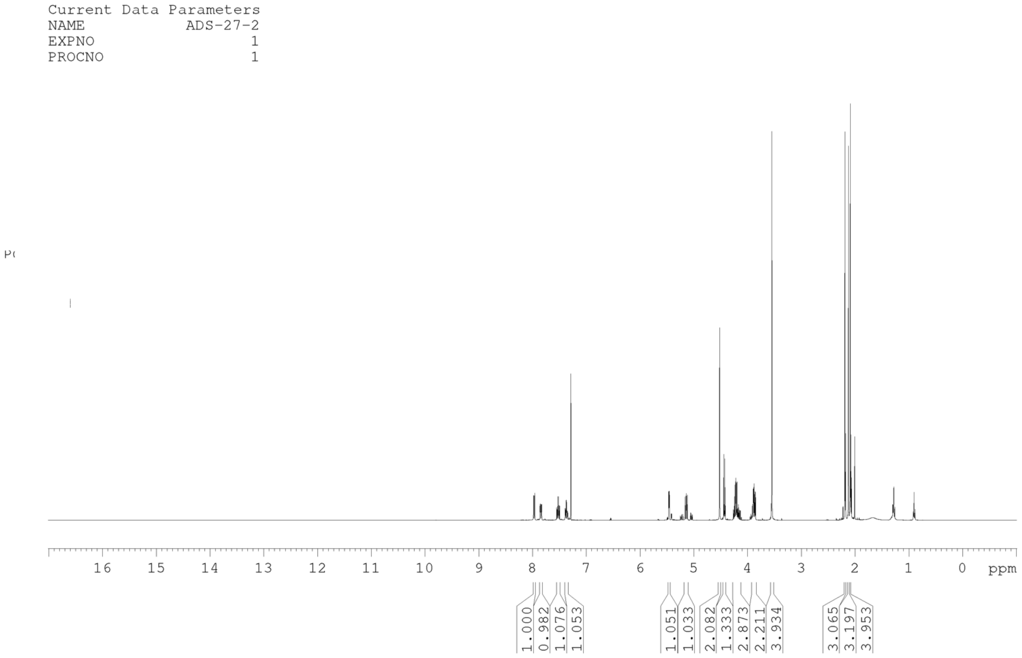

^{13}C-NMR:

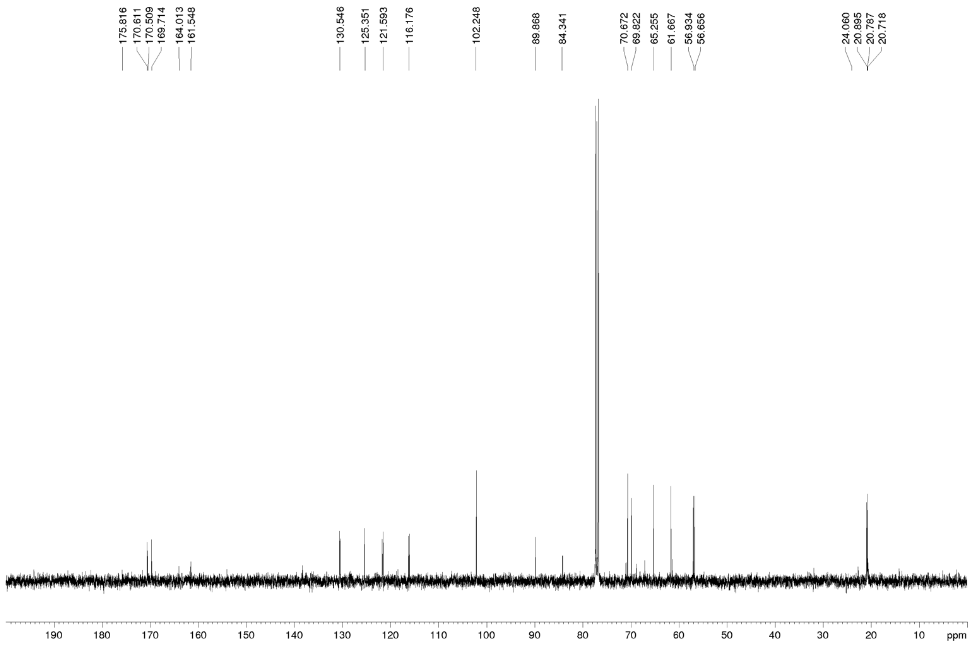

3h:

^{1}H-NMR:

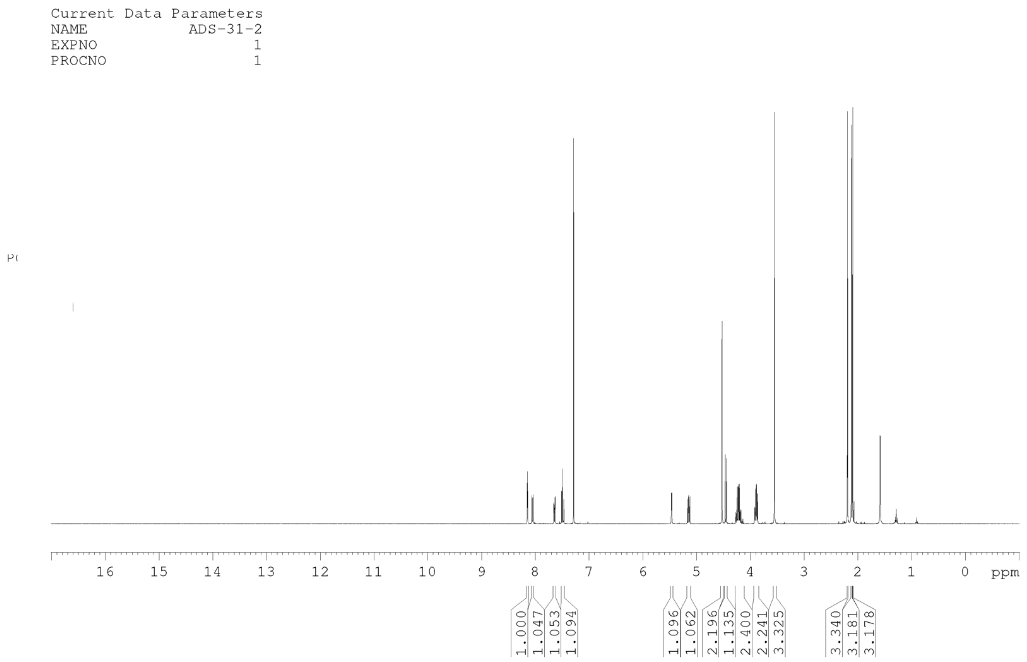

^{13}C-NMR:

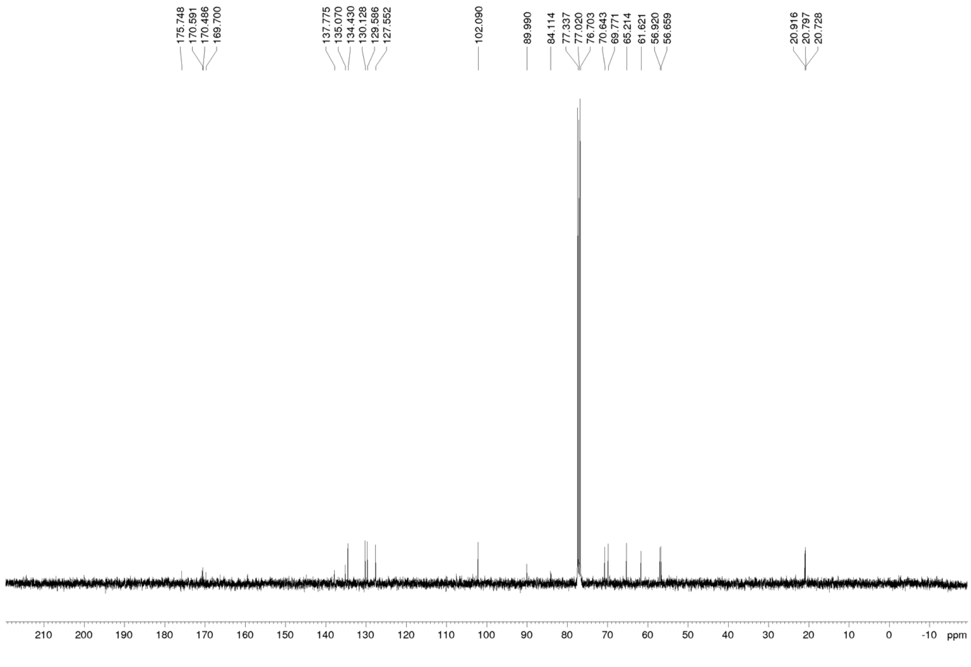

3i:

^{1}H-NMR:

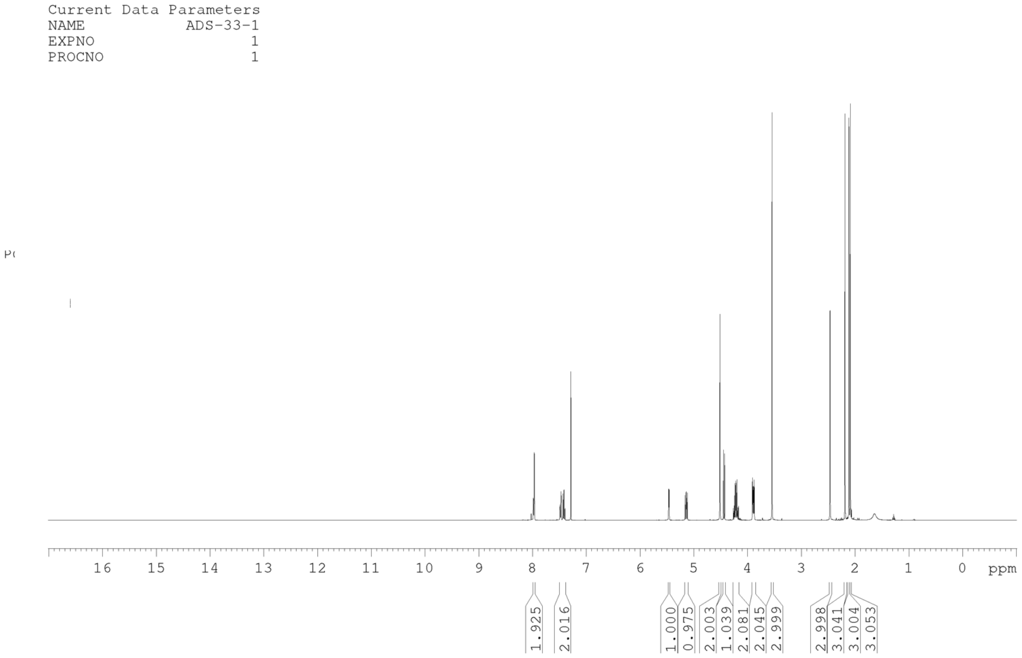

^{13}C-NMR:

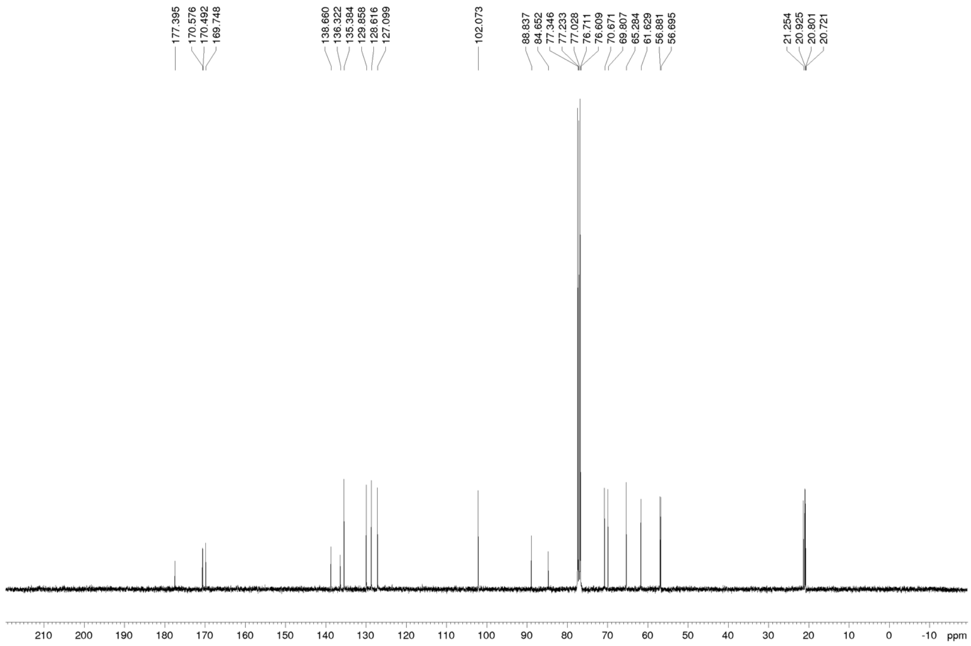

3j:

^{1}H-NMR:

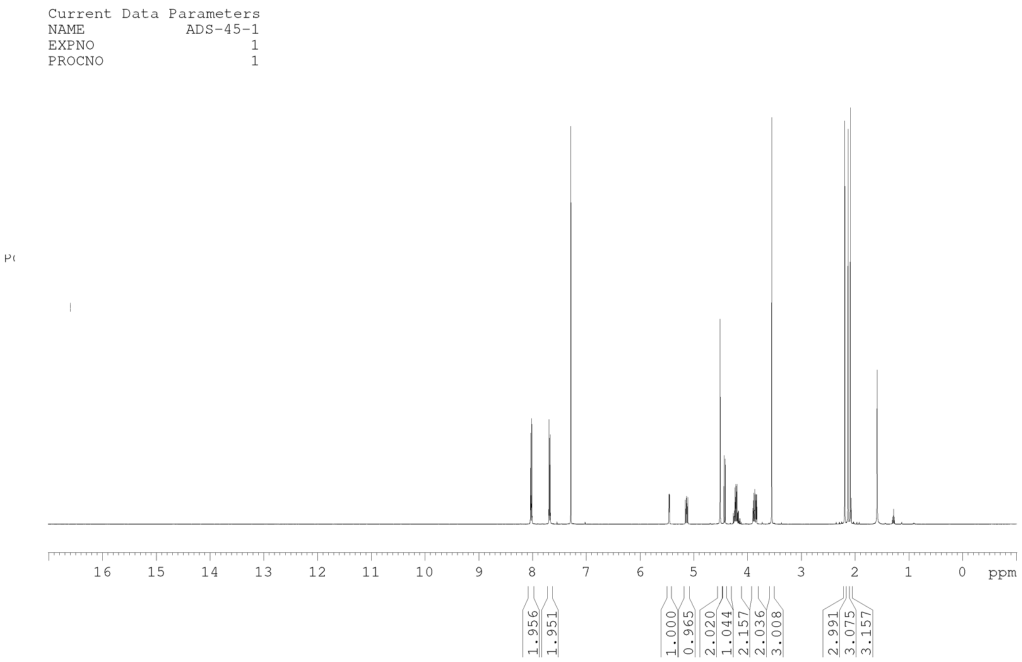

^{13}C-NMR:

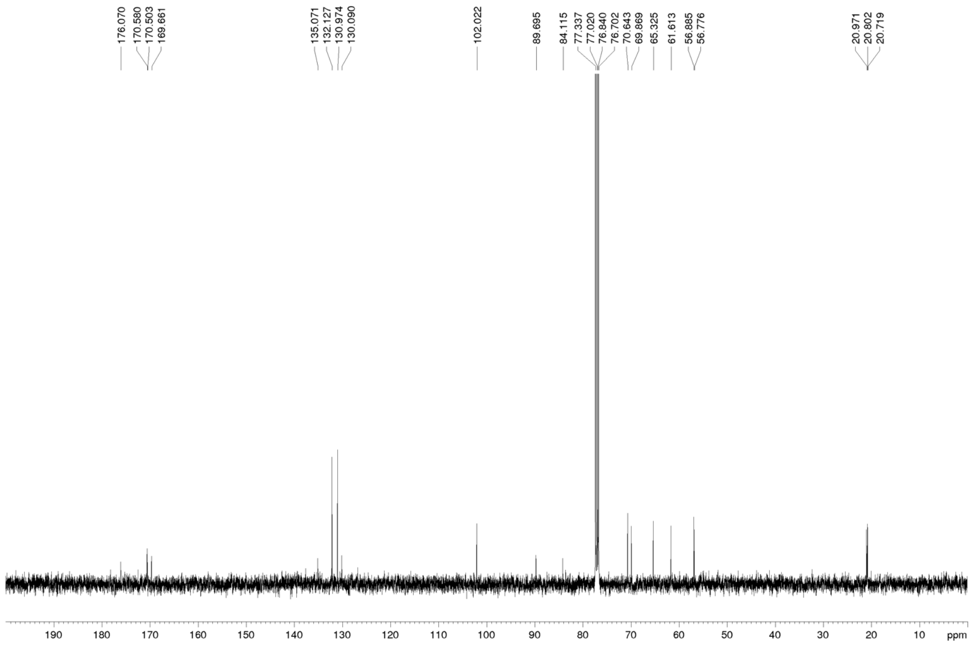

3k:

^{1}H-NMR:

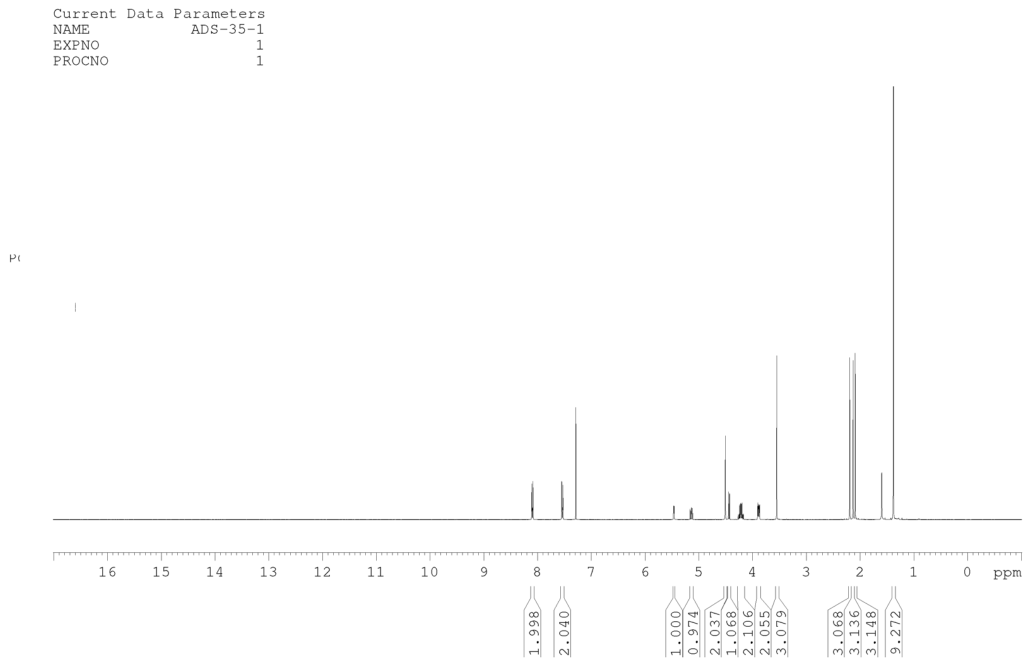

^{13}C-NMR:

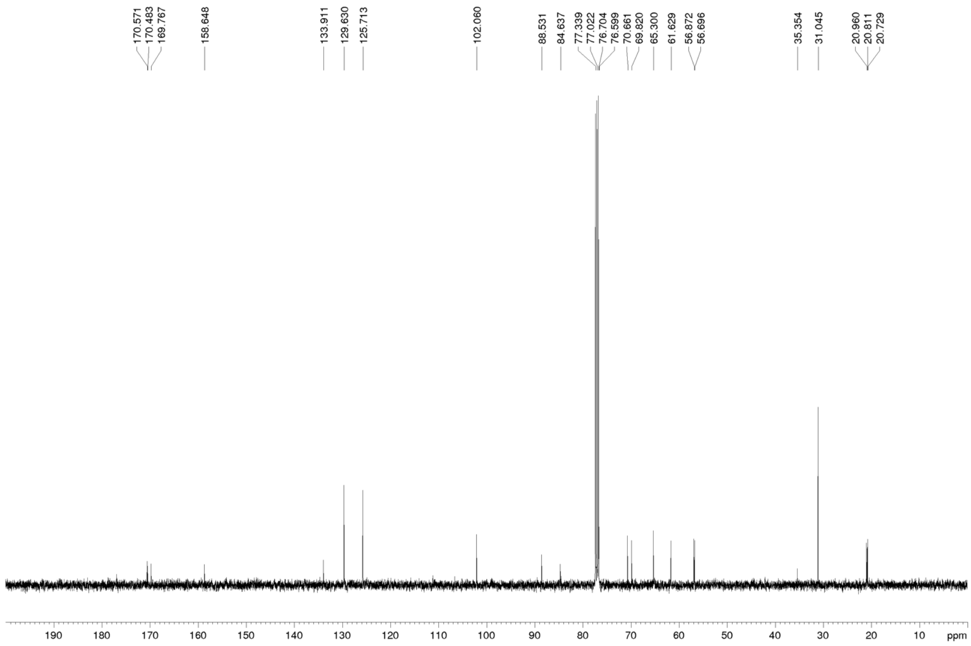

3l:

^{1}H-NMR:

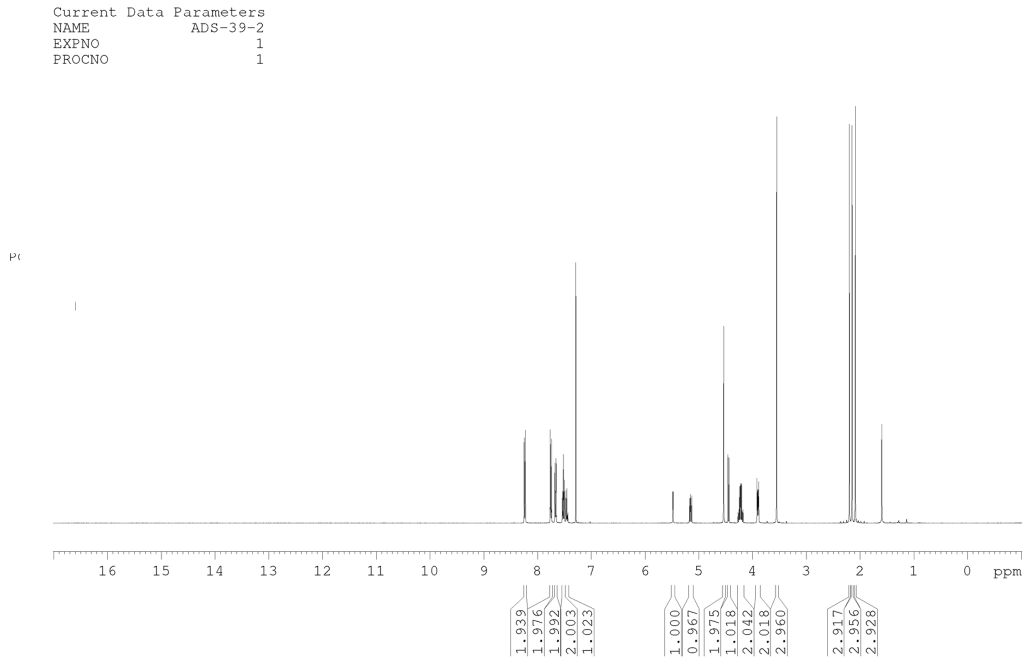

^{13}C-NMR:

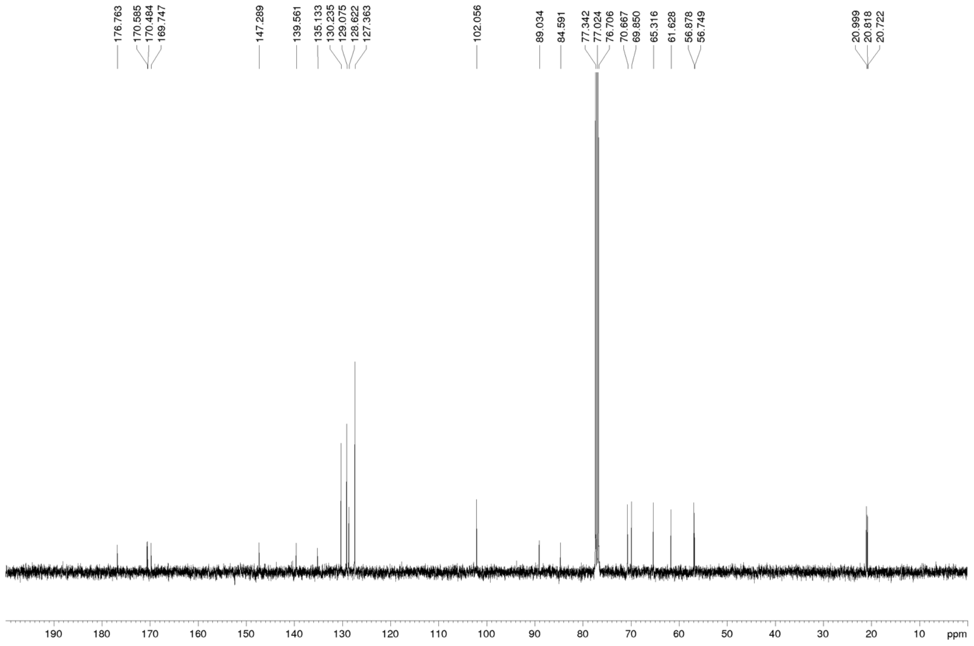

3m:

^{1}H-NMR:

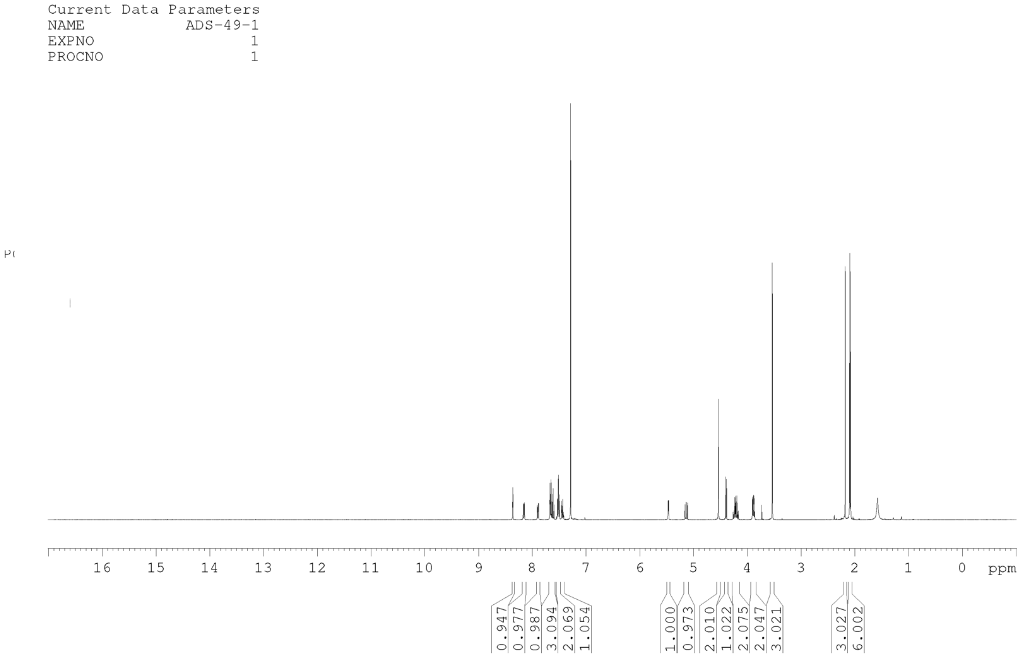

^{13}C-NMR:

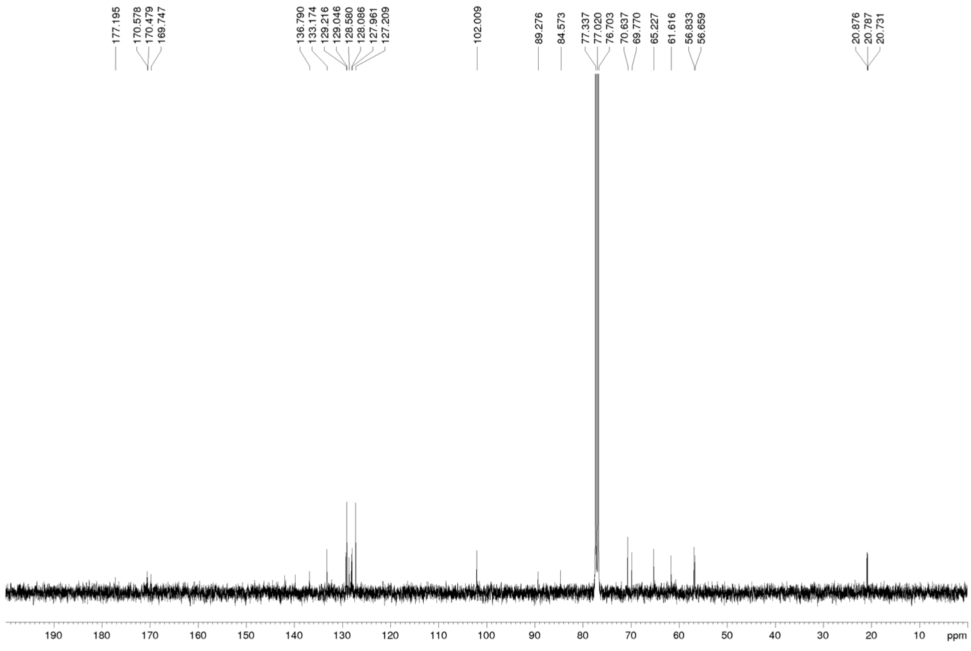

3n:

^{1}H-NMR:

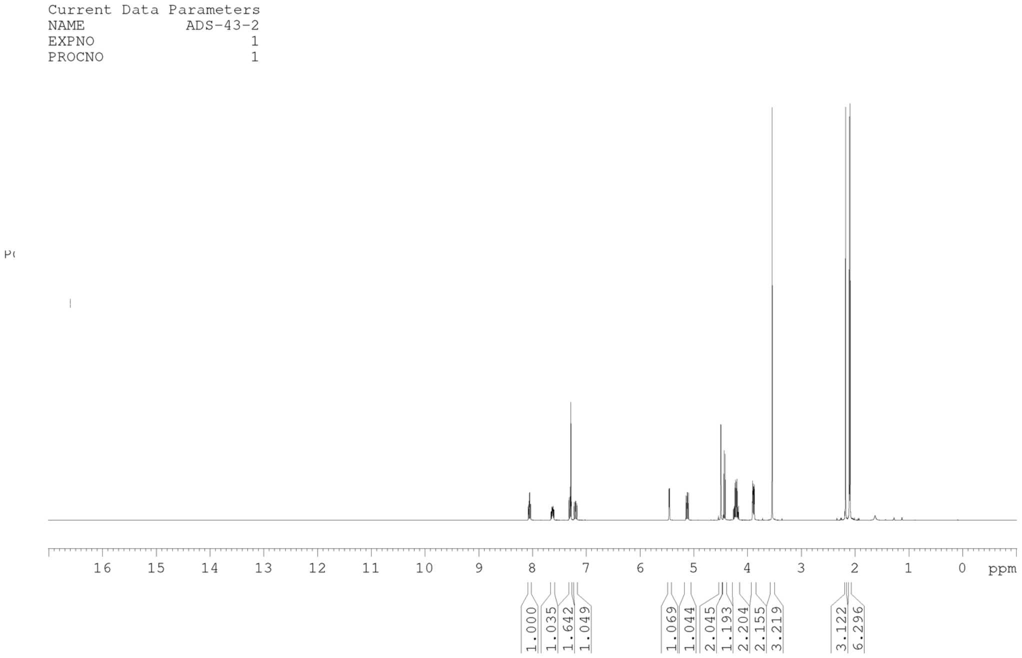

^{13}C-NMR:

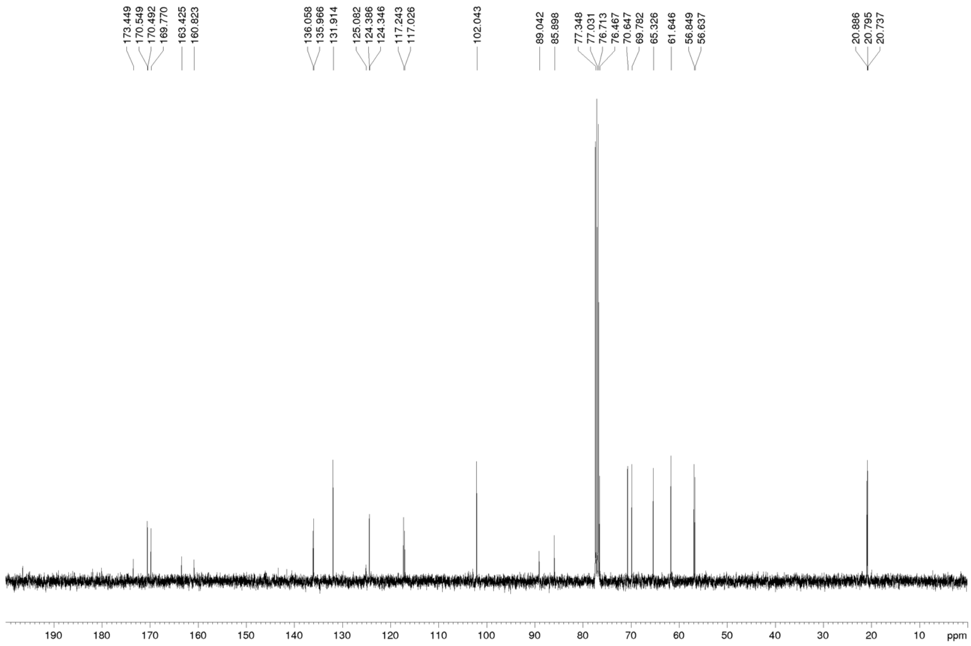

5:

^{1}H-NMR:

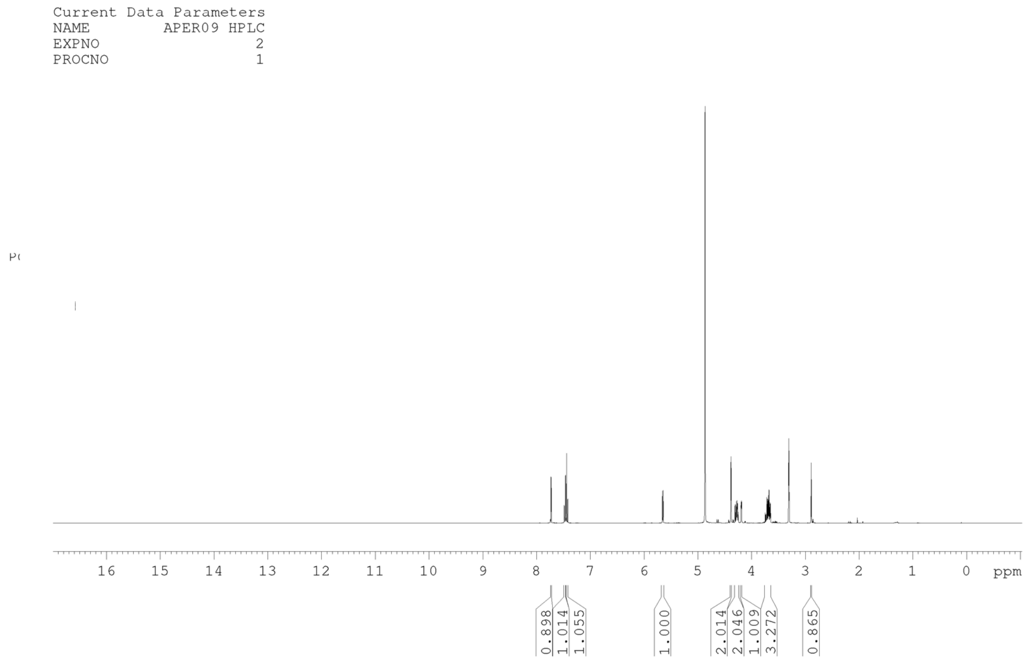

^{13}C-NMR:

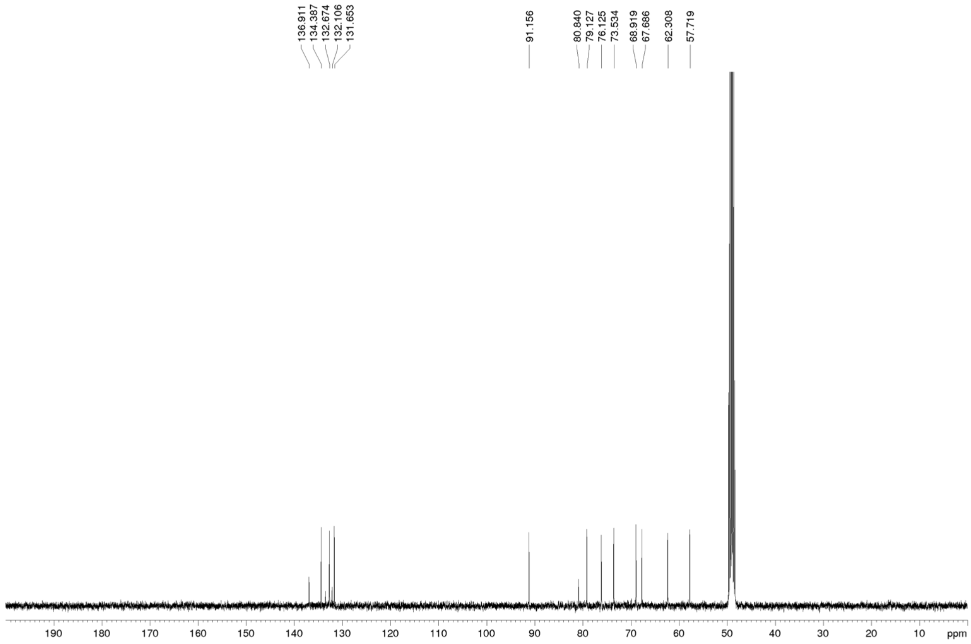

7:

^{1}H-NMR:

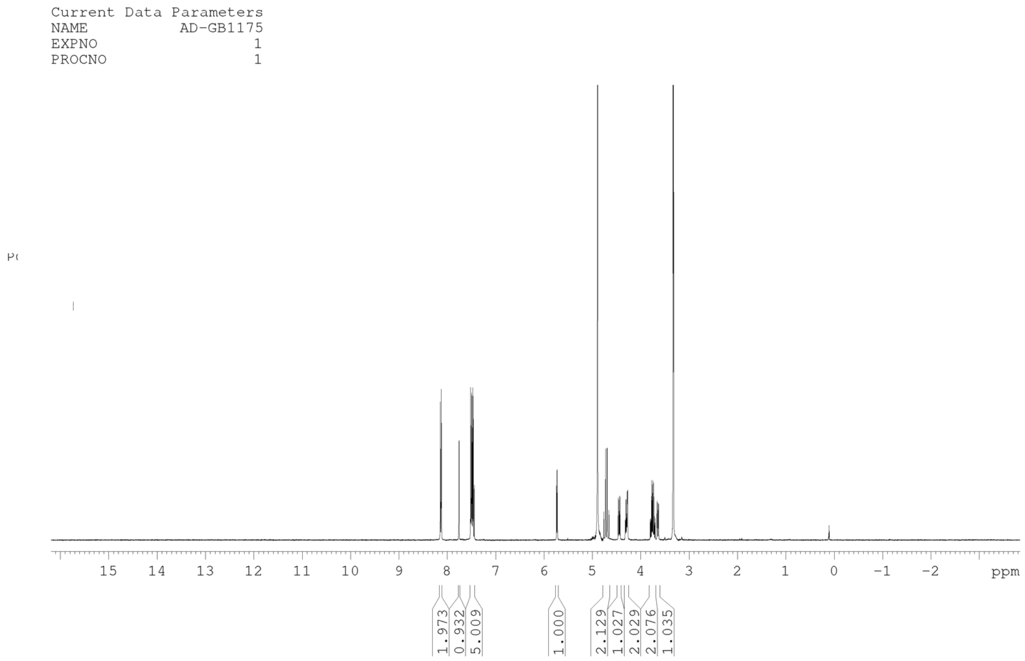

^{13}C-NMR:

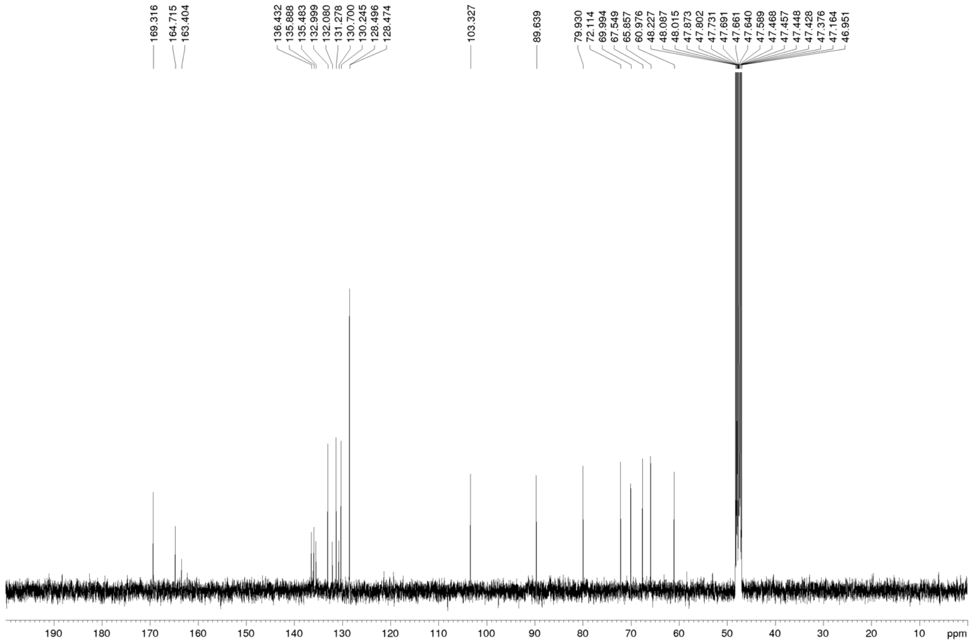

Supplement: Supplementary file 1 [file MD-010-C9MD00183B-s001.pdf]
